# Water beetle networks differences and migration between natural lakes and post-exploitation water bodies

**DOI:** 10.1038/s41598-025-00525-1

**Published:** 2025-05-07

**Authors:** Joanna Pakulnicka, Marek Kruk

**Affiliations:** 1 Department of Zoology, University of Warmia and Mazury in Olsztyn, Lodzki sq. 3, 10-727 Olsztyn, Poland; 2 Department of Applied Informatics and Mathematical Modelling, University of Warmia and Mazury in Olsztyn, Sloneczna 54, 10-719 Olsztyn, Poland

**Keywords:** Water beetles, Clay pits, Gravel pits, Migrations, Graph meta-network, Explainable machine learning, Computational models, Functional clustering, Machine learning, Statistical methods, Climate-change ecology, Community ecology, Conservation biology, Ecological modelling, Ecological networks, Ecosystem ecology, Freshwater ecology, Computational biology and bioinformatics, Ecology

## Abstract

Water deficits are a serious problem around the world, which also affects young landscapes, where lakes are most abundant. This poses a threat to many habitats and biological diversity found here. The relationships between species in the ecological networks of lakes at different stages of development and in nearby post-exploitation water bodies remain poorly understood. To better understand the functioning of beetle communities in different ecosystems, we created five network models that we subjected to graph analysis. By analysing the general attributes of the network (number of neighbours, shortest path, characteristic path length, clustering coefficient, network centralisation, network density and network heterogeneity) and those related to the nodes (NCC—Node Closeness Centrality, NBC—Node Betweenness Centrality, NDC—Node Degree Centrality) and to the edges (EBC—Edge Betweenness Centrality and correlations between the biomass of species as nodes), we were able to determine the role of each species in the networks and the relationships between the species. We then used the machine learning ensemble modelling XGBoost—SHAP to identify species that are particularly important in migrations between water bodies and to assess the direction and strength of migrations using Shapley values. Our analyses are based on faunal material from 25 lakes (mesotrophic, eutrophic, dystrophic) and 31—post-exploitation water bodies (clay pits and gravel pits) in northern Poland, in the Masurian Lake District. We found a total of 169 species representing different ecological and functional components. We have shown that the structures of the network between the biomass of species in the analysed five water types differ significantly. The highest value for network density was recorded in eutrophic lakes and clay ponds, the lowest in dystrophic lakes. In eutrophic lakes these are mainly eurybionts, in clay pits–rheophiles and in gravel pits–argilophiles and tyrphophiles. The relationship between the species with the highest NBC and EBC values is particularly important in order to maintain the stability of the network. The periphery of the network usually consists of larger predators that do not compete with each other. By analysing the migration directions of beetles between different ecosystems, we were able to demonstrate a greater affinity of the beetle fauna, especially the argilophiles (e.g. *Scarodytes halensis* and *Laccobius minutus*) inhabiting gravel pits, to dystrophic lakes. The beetles in clay pits originate mainly from mesotrophic lakes. These are mainly rheophiles, mostly weakly flying species, such as: *Haliplus fluviatilis*, *Haliplus fulvus*, *Ilybius fenestratus*, *Hygrotus vericolor* and *Haliplus flavicollis*. These species are important for the stability of ecological networks in the studied lake types. Their movements between the ecosystems studied in turn contribute to the functional connectivity between the individual lakes, which ensures the stabilisation of biotic relationships at the landscape level. At the same time, they generally also indicate the optimisation of environmental conditions in post-exploitation water bodies, which makes them potential substitute habitats for natural lakes.

**Supplementary Information:**

The online version contains supplementary material available at 10.1038/s41598-025-00525-1.

## Introduction

One of the goals of biocoenosis ecology is to identify the mechanisms that determine patterns of species richness^1–4^. Many ecologists point to the dynamic nature of biocenoses based on dispersal, i.e. the movement of organisms between available habitats, which is the result of both natural changes and anthropogenic pressures across entire landscapes^3–7^. This is in line with metapopulation theory, which has been intensively developed in recent years^8–12^. According to this theory, the structure and functioning of biocenoses are based on complex interactions between organisms that form ecological networks^10,15–18^. However, the problem of insufficient recognition of the detailed structure and functioning of networks (their attributes) between different species affects many environments, including particularly sensitive ecosystems such as freshwaters, especially lakes^5,6^.

Huge water deficits are a serious problem around the world, which also affect young post–glacial landscapes, typical of northern and central Europe, including Poland. The ecological landscapes are higher-level ecological systems composed of many ecosystems that are interconnected through the interaction of abiotic factors and biotic relationships between species^19,20^. One of the potential biotic interactions among species is competition, which in the course of evolution shapes the structure of biotic networks and trophic relationships typical of a given system – a network of trophic chains that ensures the transfer of energy through subsequent trophic levels^21^. Ecosystems are open systems, as food chains extend beyond their boundaries thanks to the migration of organisms. One of the biocenotic principles is the fact that all species enter into biocenotic relationships and are therefore all important for the stabilisation of the ecosystem. Migrations between ecosystems enable the maintenance of biodiversity and thus guarantee the stabilisation of the ecological landscape^19,20^. This is particularly important because ecological systems are dynamic and subject to changes that accelerate anthropogenic activities^22^.

Natural lakes are an essential element of the young glacial landscapes of north–central Europe, which were formed during the recession of the last, fourth ice age, the so-called Vistula Glaciation^23^. In Europe, the total number of natural lakes with an area of more than 1 ha is over 500,000. Poland has around 7000 lakes over 1 ha in area each, which corresponds to 1.42% of all lakes in Europe. They are mainly located in the Pomeranian and Masurian Lake District^24^. Despite their common history of formation, they are in different stages of ecological succession, at the end of which they will disappear completely. According to Choiński et al.^24^, only 40% of all formed lakes are still preserved, and their average age (around 2,000 years) proves their episodic character at the geological scale. The evolution of lakes essentially follows two different directions: according to the harmonic series, which is linked to the successive increase in the fertility of lakes from oligo-, meso-^25^, and according to the disharmonic series, which is linked to the evolution from dystrophic lakes in the forest to polyhumic lakes and then to peat bogs^25,26^. Although the final fate of lakes is fixed and the pace of their evolution depends on many natural factors such as climate, location and morphometry, this process is additionally accelerated by human pressures, e.g. agriculture, livestock, forestry, hydro-engineering practises, deforestation and peat mining^27–32^. For this reason, the Water Framework Directive (WFD), which constitutes the basis for developing a protection and restoration system aquatic environment, requires that EU Member States should aim to achieve good status for all surface waters and groundwaters^33^.

A side effect of anthropogenic pressure is the appearance of artificial water bodies in the landscape, which are created by filling pits with water after excavation^34–37^. They complement the natural hydrographic network and form extensive anthropogenic lake areas in many areas^38^. In the lowlands of Poland, there are a particularly large number of water bodies created by the exploitation of mineral resources such as sand, gravel or clay, which are needed for the construction of infrastructure^34,35^.

The deterioration of lake water quality, the disappearance of some habitats and, over time, of entire lakes, leads to a deterioration of fauna, a decline in local biodiversity and, in particular, the disappearance of the most sensitive species^27,31,39–41^. The most endangered part of the lake, which remains under pressure from strong external factors, is the littoral zone^31,42^. This is where the greatest species richness of macroinvertebrates is recorded, especially specialised species that are more sensitive to changes in the environment. This is a prerequisite for the use of macroinvertebrates in the monitoring of aquatic ecosystems, which is in line with the recommendations of the Water Framework Directive^43–46^. Taking into account all the consequences resulting from the transformation and disappearance of lake ecosystems, anthropogenic water bodies are becoming an important element of the landscape^34,35,37^. They take over the function of ecologically young lake ecosystems and become a substitute habitat, a kind of refuge into which the lake fauna invades, especially the most sensitive and endangered species^21,47–49^.

In the extensive literature on the entomofauna of aquatic habitats, there are many papers on aquatic beetles inhabiting lakes, but knowledge of this group of insects in anthropogenic water bodies is very scarce^35,50–53^. Therefore, little is known about the structure and functioning of ecological networks in them, which are characterised by trophic interactions (food chain) between species, which in turn are fragments of complex food webs^5,6,54,55^. Little is known about the mechanisms of migration (intensity and direction) of aquatic organisms between different ecosystems that shape the structures of fauna in supra–ecosystemic systems at the landscape scale^21,56–59^. Most of the available studies concern river valleys^60–66^. Only a few of these studies concern particularly well-migrating organisms, i.e. water beetles^67–72^. Many hydrobiologists now refer to water beetles as bioindicators of both the ecological status of water bodies^45,46,57,73–75^ and the biodiversity of the entire macrobenthos of freshwater ecosystems^46,73,76^. Beetles are usually very abundant in various aquatic environments. At the same time, they exhibit great species, ecological and functional diversity. As adults, they show high mobility, which allows them to respond quickly to environmental stress and even migrate over long distances^36,56,57–59^. Migrations between ecosystems are important for maintaining regional biodiversity, which influences the maintenance of dynamic equilibrium in the ecological landscape^22,41^.

In this context, it is important to recognise the mechanisms of beetle migration, especially between disappearing lakes and relatively young anthropogenic water bodies. These are complex problems that require the collection of many field observations and tools that allow a difficult interpretation of the complex relationships between species that occur in multidimensional ecological systems, as emphasised by, among others^55,77–81^. In recent years, modelling based on machine learning algorithms has become a useful research tool in the ecology of aquatic ecosystems. Høye et al.^82^ pointed out the need for a broader application of machine learning methods in the study of insect communities. The use of modelling based on boosting and SHAP algorithms to analyse the interactions of aquatic species has been applied in the study of their properties to assess water quality in microbiological studies in coastal waters^83,84^, in the analysis of the response of lagoon water properties to weather dynamics^81^ or in the analysis of the biocenotic effects of climate warming on the zooplankton community^85^. In the last two articles, the applied methods of the explainable machine learning of XGBoost–SHAP and their effectiveness in ecological research are discussed in detail. Their evaluation enables a more thorough analysis of the structures of various biocenoses, their formation and functioning under changing environmental conditions, as well as the assessment of the role of individual species in the cohesion, i.e. the durability of the analysed ecological networks^55,77–81^. Machine learning is a method to support ecosystem management^85,86^. This includes predicting the direction of adaptation of biocenotic systems to changing environmental conditions, e.g. climatic or anthropogenic^78,87,88^ and can also help in finding ways to restore already degraded habitats^5,6^.

The aim of our research was: (1) to compare the characteristics of the networks of species in beetle communities in mesotrophic, eutrophic and dystrophic lakes and anthropogenic water bodies—clay pits and gravel pits, (2) to determine the importance of individual species and the mutual connections between them in these networks, (3) to determine the role of distinctive and functional elements in the structure of the network in specific types of water bodies, (4) to identify species that show significant migration tendencies from specific water body types to clay and gravel pits and from anthropogenic water bodies to specific water body types, and to determine the species range and intensity of these migrations, (5) to specify species that are important for functional uniformity in landscapes.

## Results

### General characteristics of the collected material

In total, we collected 19,923 beetles representing 167 species with a total wet biomass of 367,119.81 mg (Tables 1, S1). The species diversity in the samples collected in in different types of water bodies studied was 72–114, with only 25 species occurring in all types of water bodies. We found the largest number of species in eutrophic lakes, where we also collected the most beetles. The least species-rich material came from dystrophic lakes. The fewest beetles were found in mesotrophic lakes.

**Table 1:** General characteristics of the material. *N* abundance, *S* number of species, $\:\stackrel{-}{\text{x}}$mean, *SD* standard deviation, *n* number of samples.

Parameters	Clay pits (*n* = 229)	Gravel pits (*n* = 243)	Mesotrophic lakes (*n* = 264)	Eutrophic lakes (*n* = 260)	Dystrophic lakes (*n* = 100)	Total (1096)
Abundance	2974	5150	1301	8514	1984	19,923
Min–max	4–641	13–1087	59–402	140–5838	5–724	
$\:\stackrel{-}{\text{x}}\:$± SD	198.3 ± 178.1	171.7 ± 221.1	260.2 ± 154.9	1419 ± 2236.7	132.3 ± 188.8	
Number of species	100	106	86	114	72	167
Min–max	2–53	2–42	14–48	22–70	3–34	3–114
Total biomass (mg)	64,196.31	76,989.65	49,873.85	116,510.26	59,549.74	367,119.81
Ecological groups—N, (S)						
Eurybionts (%)	1927 (54) (64.79)	833 (52) (16.17)	575 (46) (44.20)	5119 (63) (60.12)	1020 (40) (51.41)	9474 (89)
Tyrphophiles (%)	282 (23) (9.48)	170 (23) (3.30)	105 (16) (8.07)	162 (22) (1.90)	884 (22) (44.56)	1603 (37)
Argilophiles (%)	343 (13) (11.53)	4080 (19) (79.22)	145 (9) (11.14)	628 (14) (7.38)	33 (8) (1.66)	5229 (25)
Rheophiles (%)	406 (8) (13.65)	66 (10) (1.28)	473 (13) (36.36)	2597 (12) (30.50)	47 (4) (2.37)	3589 (16)
Rheobionts (%)	16 (1) (0.54)	1 (1) (0.02)	3 (1) (0.23)	8 (3) (0.09)	0 (0) (0.0)	28 (5)

The species richness found in the analysed water body types differed significantly (Kruskal–Wallis test: H(4,*N* = 71) = 11.34, *p* = 0.02 (Table 2). We also found significant differences in the number of beetles collected (Kruskal–Wallis test: H(4,*N* = 71) = 19.49, *p* = 0.0006). Statistically significant differences between the types of water bodies tested (p-value for multiple comparisons) are shown in Table 2.

**Table 2:** Differences between parameters in the analyzed types of water bodies. Results of ANOVA Kruskal – Wallis test: H – (statistics), df – degree of freedom, p – p value, N – abundance, S – number of species.

Parameter	H	df	*p*	Post–hoc (*p*)
Abundance (N)	11.34	4, *N* = 71	0.02	Dystrophic–eutrophic (0.03)
Number of species (S)	19.49	4, *N* = 71	0.0006	Dystrophic–eutrophic (0.02) Eutrophic–gravel pits (0.009)
Biomass	15.67	4, *N* = 71	0.005	Dystrophic–eutrophic (0.03) Eutrophic–gravel pits (0.001)
Ecological groups (N, S)				
Eurybionts	22.28	4, *N* = 71	0.0002	Clay pits–gravel pits (0.02) Eutrophic–gravel pits (0.001)
	21.25	4, *N* = 71	0.0003	Clay pits–gravel pits (0.02)
Tyrphophiles	16.13	4, *N* = 71	0.02	Dystrophic–gravel pits (0.02)
	14.04	4, *N* = 71	0.007	Eutrophic–gravel pits (0.046)
Argilophiles	29.98	4, *N* = 71	0.00001	Dystrophic–gravel pits (0.0001)
	19.26	4, *N* = 71	0,0007	Fystrophic–gravel pits (0.0002)
Rheophiles	30.11	4, *N* = 71	0.00001	Dystrophic–eutrophic (0.0001) Dystrophic–mesotrophic (0.01) Eutrophic–gravel pits (0.004) Gravel pits–mesotrophic (0.005)
	30.57	4, *N* = 71	0.0001	Dystrophic–eutrophic (0.0006) Dystrophic–mesotrophic (0.005) Eutrophic–gravel pits (0.0007) Gravel pits–mesotrophic (0.007)
Rheobionts	0.0	4, *N* = 71	1.0	–

The most numerous species in the collected material were: *Noterus crassicornis* (16.31%), *Laccobius minutus* (11.47%), *Anacaena lutescens* (17.1%), *Scarodytes halensis* (10.11%), followed by *Haliplus immaculatus* (8.04%), *Haliplus flavicollis* (7.95%), *Hygrotus versicolor* (3.82%) and *A. lutescens* (3.21%). The species with the highest individual biomass were: *Cybister lateralimarginalis*, *Dytiscus dimidiatus*, *D. marginalis*, *D. circumcinctus* and *Hydrophilus aterrimus*. The individual water types were characterised by different numbers of the most numerous species but were always dominated by *Noterus crassicornis* and *L. minutus* (Table S1). The core of the fauna was formed by eurybionts (39.52%), which also exhibited the greatest species diversity in the entire material (86 species). A significantly lower species richness was observed in the tyrphophiles (37) and argilophiles (25). Argilophiles (24.2% of the total material) and rheophiles (18.01%) were of great quantitative importance (Table 1). The distinguished ecological groups contributed differently to the fauna in the analysed types of water bodies. Eurybionts were of greatest quantitative and qualitative importance in eutrophic lakes and clay pits. Rheophiles, on the other hand, were most numerous in eutrophic, mesotrophic lakes and clay pits, while argilophiles were found in gravel pits and tyrphophiles in dystrophic lakes. (Table 1). Significant statistical differences between the analysed environments (p-value for multiple comparisons) are listed in Table 2.

### Network structure

**Fig. 1:**
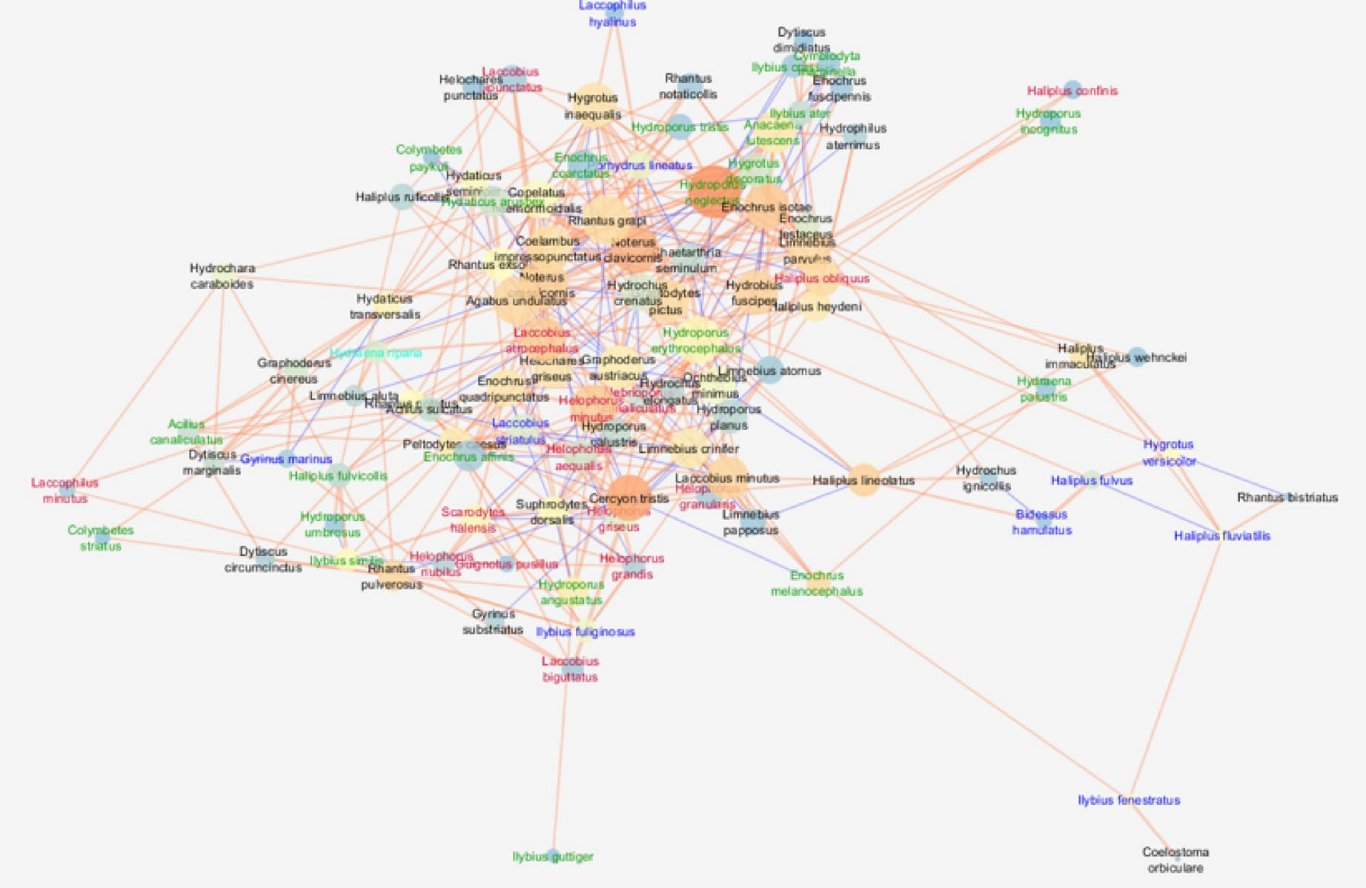
Network of correlations between beetle species in clay pits with node closeness centrality (NCC), node betweenness centrality (NBC) and correlation coefficient (R). Node size is proportional to the NCC measure; node colour from blue (dark) to orange (light) is proportional to the NBC measure; edge thickness is proportional to the correlation coefficient R. Sign of the relationship: a light orange edge represents positive relationships between nodes, while a dark blue edge represents negative relationships. The font colour means: red—argilophiles, blue—rheophiles, light green—rheobionts, dark green—tyrphophiles, black—eurybionts. The graph was generated using the Metscape application in the Cytoscape 3.7.2 package, https://cytoscape.org.

The analysed network in clay pits was characterised by a moderate cohesion metric (clustering coefficient = 0.349), a density metric (0.079) and a centrality coefficient (centralisation) (0.113). At the same time, the highest values are for the number of nodes (species) (101), the average number of neighbours (7.901) per species (node), i.e. the number of interspecific interactions, as well as the values of the parameters describing the communication pathways between the taxa. This means the highest number of direct and indirect connections between species. In these waters, the shortest paths (10100) were found, indicating the highest number of highest correlations between species in the network, as well as a relatively high value of the so–called characteristic path length (3.718), indicating the presence of taxa communicating with the highest number of species (Fig. 1; Table 3).

**Table 3:** General attributes of the water beetles network in compared regions.

Atribute	Habitats				
	Clay pits	Gravel pits	Mesotropic lakes	Eutrophic lakes	Dystrophic lakes
Clustering coefcient	0.349	0.408	0.365	0.334	0.294
Network centralization	0.113	0.101	0.220	0.193	0.076
Shortest paths	10,100	8556	5402	10,302	4556
Characteristic path length	3.718	2.965	2.670	2.687	3.109
Average number of neighbours	7.901	6.882	6.378	8.843	5.029
Number of nodes	101	93	74	102	68
Network density	0.079	0.075	0.087	0.080	0.075
Network heterogeneity	0.594	0.558	0.727	0.643	0.499

**Fig. 2:**
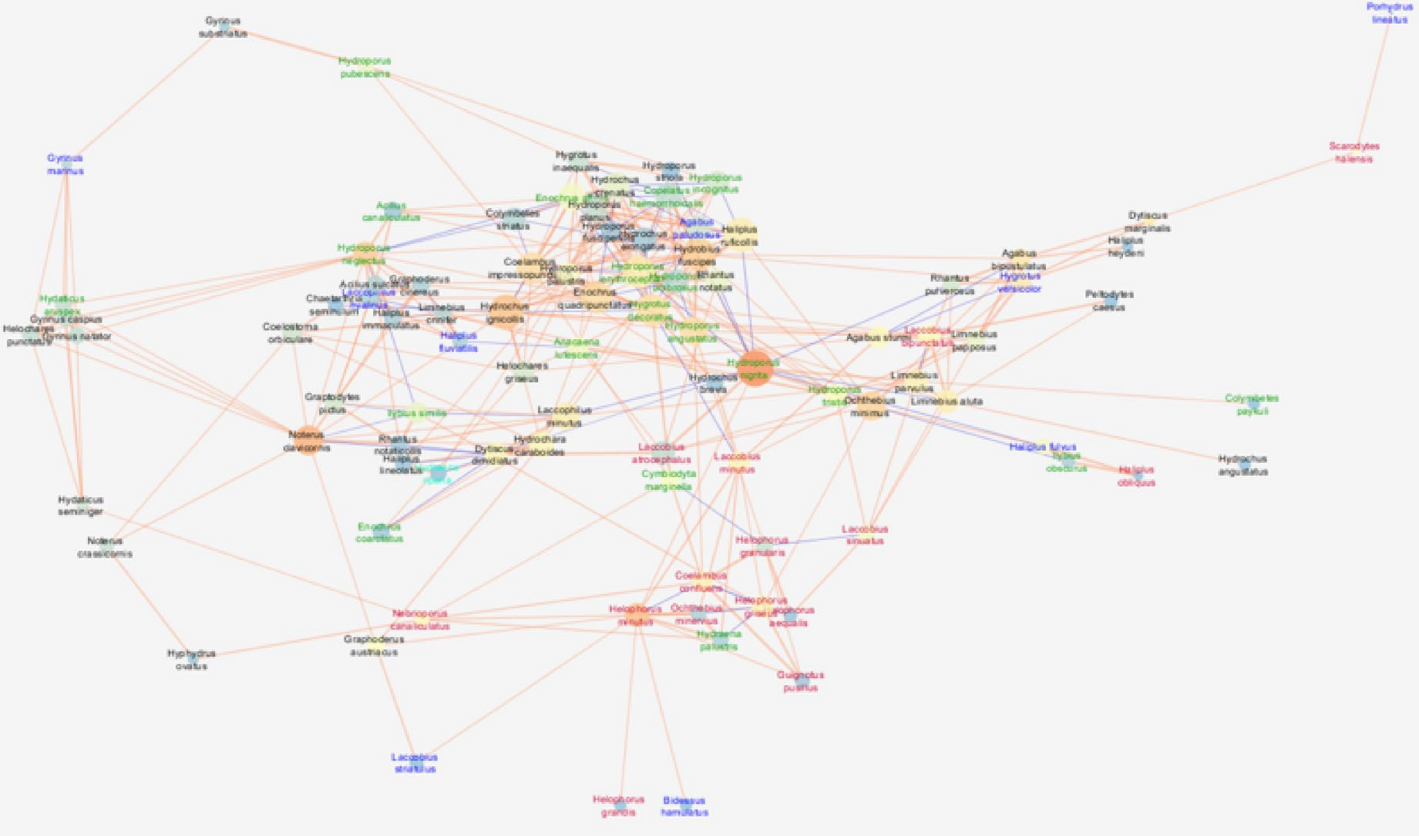
Network of correlations between beetle species in gravel pits with node closeness centrality (NCC), node betweenness centrality (NBC) and correlation coefficient (R). See the legend and explanations in Fig. 1 (clay pits). The graph was generated using the Metscape application in the Cytoscape 3.7.2 package, https://cytoscape.org.

The network of interspecific interactions observed in the gravel pits was characterised by the highest cohesion metric (clustering coefficient = 0.408), while the network density was the lowest (0.075). The other attributes describing the networks had moderate values (Fig. 2).

**Fig. 3:**
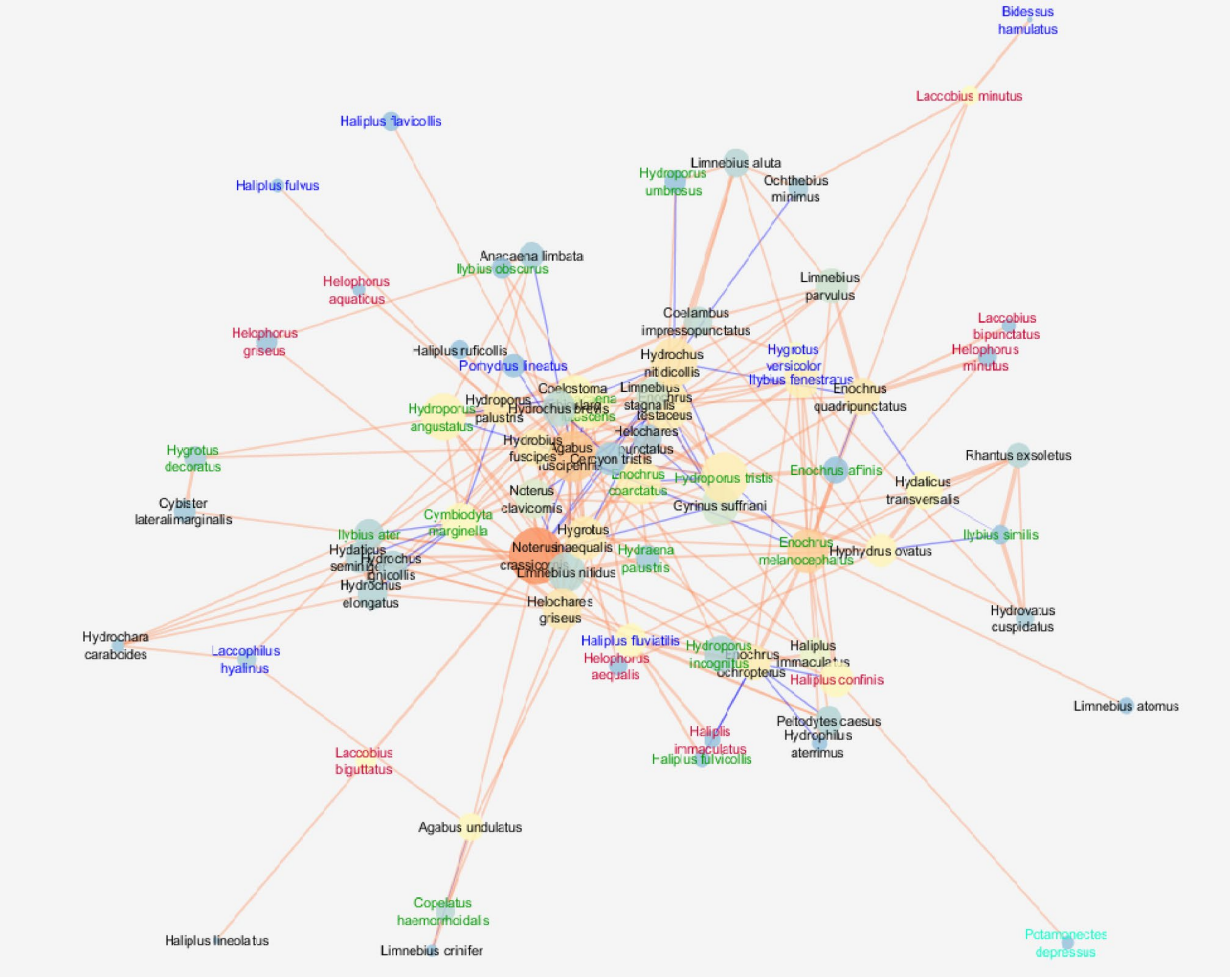
Network of correlations between beetle species in mesotrophic lakes with node closeness centrality (NCC), node betweenness centrality (NBC) and correlation coefficient (R). See the legend and explanations in Fig. 1 (clay pits). The graph was generated using the Metscape application in the Cytoscape 3.7.2 package, https://cytoscape.org.

The network observed in mesotrophic lakes was characterised by a relatively high cohesion metric (clustering coefficient = 0.365), while the centrality coefficient (centralisation) (0.220) and density metric (0.087) were the highest. It was also characterised by the highest fragmentation, as indicated by the highest value of the network heterogeneity parameter (0.727) (Fig. 3; Table 3).

**Fig. 4:**
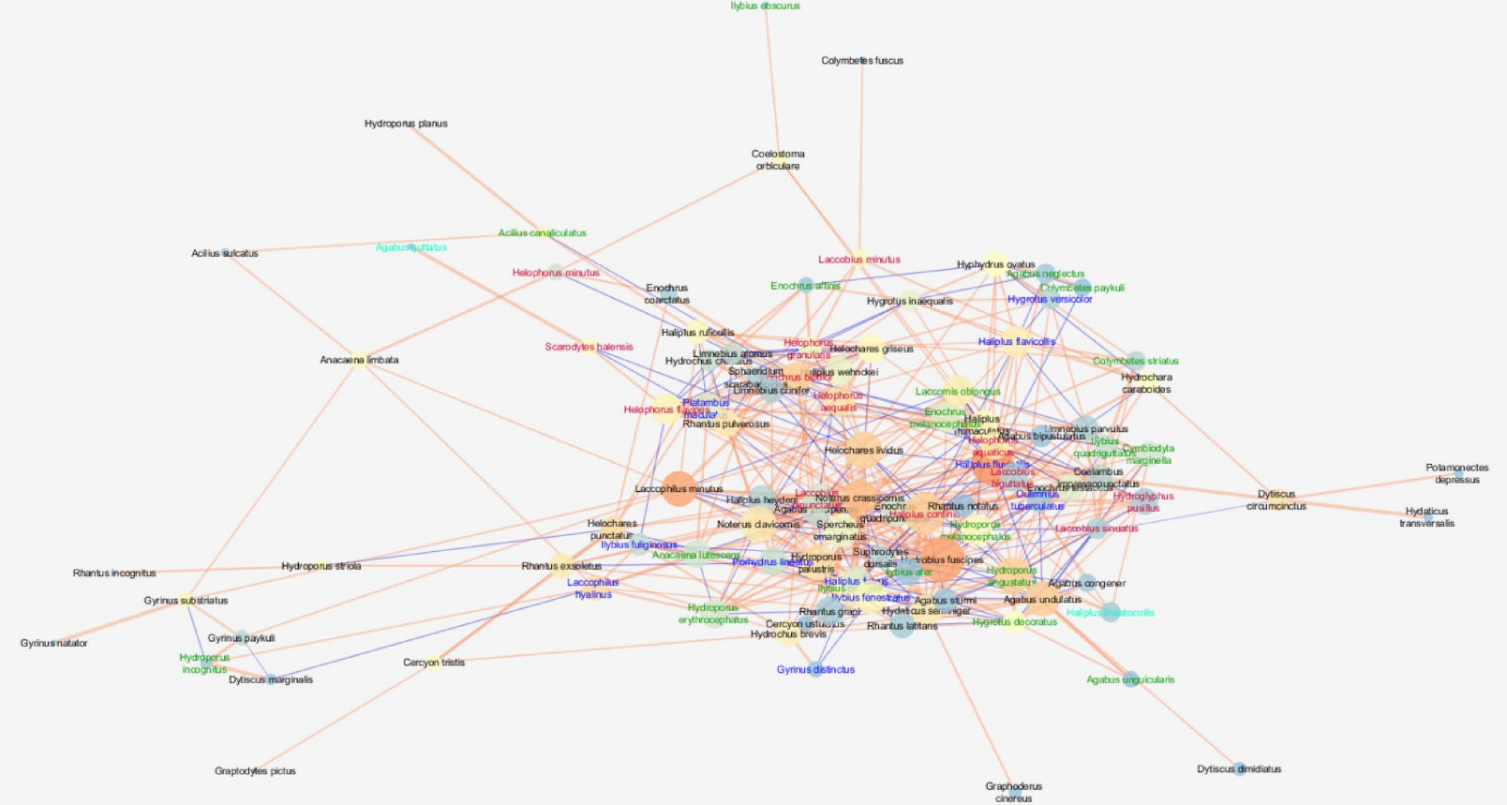
Network of correlations between beetle species in eutrophic lakes with node closeness centrality (NCC), node betweenness centrality (NBC) and correlation coefficient (R). See the legend and explanations in Fig. 1 (clay pits). The graph was generated using the Metscape application in the Cytoscape 3.7.2 package, https://cytoscape.org.

The analysed network in eutrophic lakes was characterised by a moderate cohesion metric (clustering coefficient = 0.334) and density metric (0.079) as well as a relatively high centrality coefficient (centralisation) (0.193). At the same time, the highest values are for the number of nodes (species) (102), the average number of neighbours (8,843) per species (node), i.e. the number of interspecific interactions, and the values of the parameters describing the communication pathways between the taxa. In these lakes, the shortest paths (10302) were found, indicating the highest number of the highest correlations between species in the network, as well as a relatively high value of the so–called characteristic path length (2.687), indicating the presence of taxa communicating with the highest number of species (Fig. 4; Table 3).

**Fig. 5:**
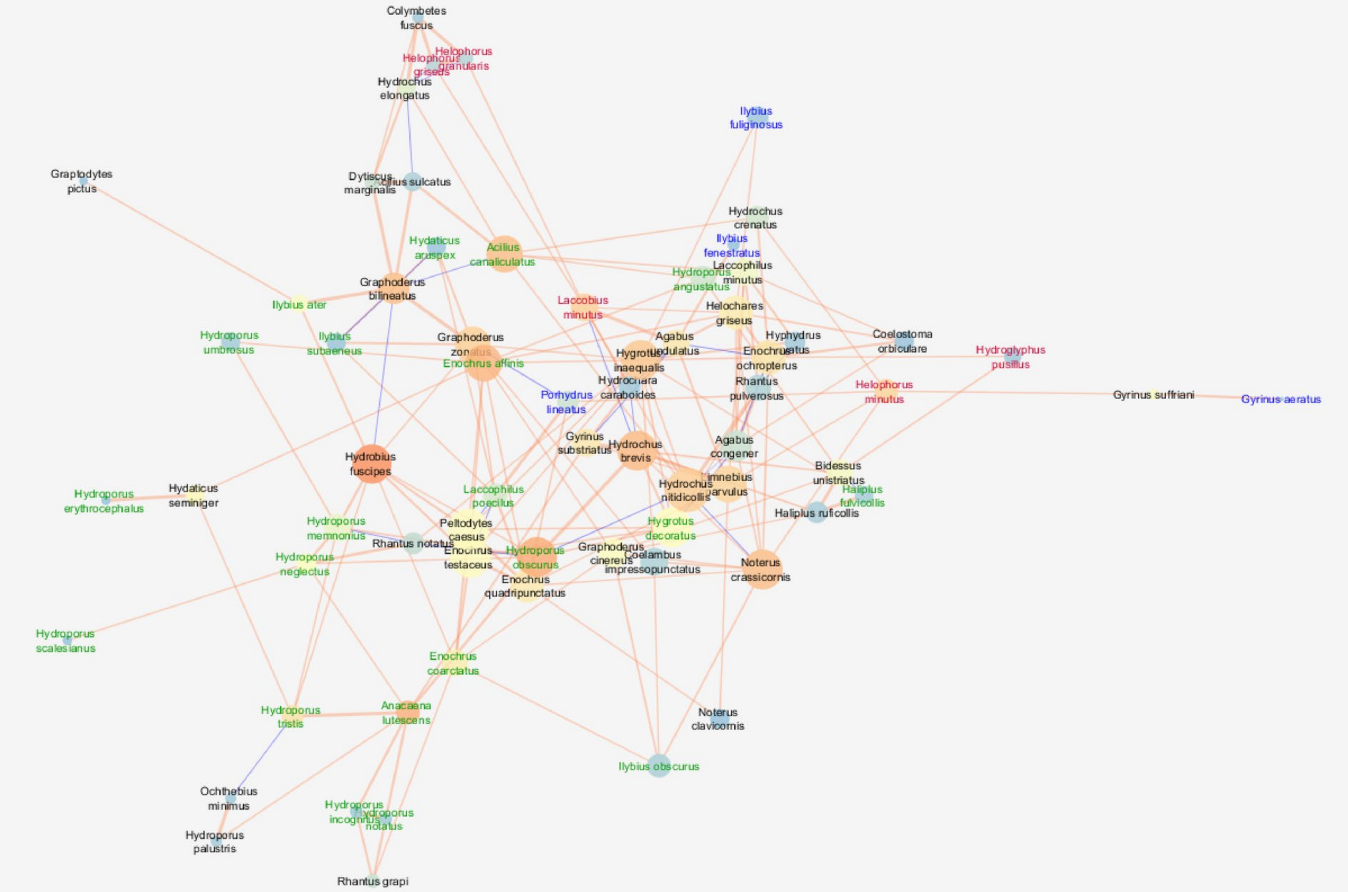
Network of correlations between beetle species in dystrophic lakes with node closeness centrality (NCC), node betweenness centrality (NBC) and correlation coefficient (R). See the legend and explanations in Fig. 1 (clay pits). The graph was generated using the Metscape application in the Cytoscape 3.7.2 package, https://cytoscape.org.

The lowest cohesion and the lowest density characterise the network in dystrophic lakes. This is confirmed by the values for clustering (0.294) and density (0.075) as well as centralisation (0.076). The network also had the lowest values for the average number of neighbours (5.029) per species (node) and the number of shortest paths (4556). At the same time, the presence of taxa communicating with the largest number of species was the highest among the five analysed networks, as shown by the value of the characteristic path length (5.029) (Fig. 5; Table 3).

### Interspecific relationships in water beetles networks

An important measure of interspecific relationships is node degree centrality (NDC), which describes the number of direct connections with a particular taxon (node) (Table 4). The highest NDC values were found in eutrophic lakes for *Hydrobius fuscipes* (28), *Haliplus confinis* (26), *H. fluviatilis* (19) and *Agabus undulatus* (10). The maximum NDC values were lower in the other habitats. In mesotrophic lakes they were observed for *Noterus crassicornis* (22) as well as *Agabus fuscipennis* (16), *Hydroporus tristis* (15) and *Enochrus melanocephalus* (15), in clay pits – for *H. neglectus* (19), *Noterus crassicornis* (18), *N. clavicornis* (18), *Enochrus isotae* (18) and *Helophorus minutus* (16), in gravel pits – for *Agabus undulatus*, *Hygrotus decoratus* and *Hydroporus nigrita* (16), as well as for *Hygrotus inaequalis* and *H. neglectus* (15). The lowest NDC values were determined in dystrophic lakes for *N. crassicornis* (10), as well as for *H. fuscipes* (9), *Enochrus quadripunctatus*, *E. affinis*, *Hygrotus decoratus* (8) and *A. lutescens* (7) (Table 4).

**Table 4:** Water beetle species with the highest net attribute. NCC node closeness centrality, NBC node betweenness centrality, NDC node degree centrality. *The most numerous species.

Species	Clay pits			Gravel pits			Mesotrophic lakes			Eutrophic lakes			Dystrophic lakes		
	NBC 0–0.08	NCC 0.22–0.48	NDC 1–19	NBC 0–0.14	NCC 0.17–0.45	NDC 1–161	NBC 0–0.22	NCC 0.25–0.52	NDC 1–22	NBC 0–0.09	NCC 0.22–0.51	NDC 1–28	NBC 0–0.12	NCC 0.20–0.41	NDC 1–10
*Hydroporus neglectus*	0.08	0.48	19												
Noterus crassicornis*	0.05	0.46	19				0.22	0.52	22	0.06	0.46	20	0.09	0.40	10
*Rhantus grapi*	0.04	0.46	17												
*Agabus undulatus*	0.04	0.46	16							0.06	0.45	20			
*Noterus clavicornis*	0.06	0.46	18	0.10	0.41	15									
*Helophorus minutus*	0.05	0.46	16												
*Hydroporus erythrocephalus*	0.02	0.46	13	0.04	0.43	15									
*Coelambus impressopunctatus*	0.04	0.45	16	0.06	0.42	16									
*Cercyon tristis*	0.07	0.45	13												
*Enochrus isotae*	0.05	0.45	18												
*Laccobius atrocephalus*	0.05	0.45	12												
*Hygrotus inaequalis*	0.03	0.45	16				0.05	0.45	10				0.08	0.40	10
*Graptodytes pictus*	0.04	0.44	11												
*Scarodytes halensis*	0,02	0.40	11												
*Hydrobius fuscipes*	0.04	0.44	14	0.07	0.43	14				0.09	0.51	28	0.12	0.40	9
Laccobius minutus*	0.04	0.46	13				0.03	0.33	4				0.07	0.36	7
Anacaena lutescens*	0.04	0.44	16	0.01	0.36	6	0.05	0.46	11				0.09	0.36	7
*Hydroporus nigrita*				0.14	0.45	15									
*Hydrochus ignicollis*				0.09	0.44	15									
*Haliplus ruficollis*				0.04	0.44	13									
*Hygrotus decoratus*				0.06	0.42	16							0.03	0.40	8
*Enochrus quadripunctatus*				0.06	0.42	13									
*Ochthebius minimus*				0.06	0.41	10									
*Hydroporus angustatus*				0.06	0.41	12	0.05	0.47	12						
*Enochrus affinis*				0.02	0.40	12							0.07	0.41	8
*Agabus fuscipennis*							0.12	0.49	16						
*Hydroporus tristis*							0.05	0.49	15						
*Enochrus testaceus*							0.05	0.49	14						
*Enochrus melanocephalus*							0.11	0.45	15						
*Enochrus coarctatus*							0.04	0.47	13						
*Hydrochus nitidicollis*							0.07	0.48	16				0.07	0.41	8
*Coelostoma orbiculare*							0.03	0.45	14						
Haliplus flavicollis*							0	0.33	1	0.03	0.44	15			
*Haliplus fluviatilis*	0.01	0.30	5				0.03	0.41	8	0.10	0,5	19			
*Haliplus fulvus*	0.01	0.32	4				0	0.30	1						
*Haliplus confinis*							0.04	0.42	5	0.05	0.49	26			
*Haliplus immaculatus*							0.02	0.40	6	0.01	0.43	14			
Hygrotus versicolor*							0.02	0.40	6	0.005	0.37	7			
*Ilybius fenestratus*	0.02	0.28	3				0.05	0.43	8	0.02	0.47	13	0	0.26	1
*Bidessus hamulatus*	0.001	0.30	3				0	0.25	1						
*Potamonectes depressus*							0	0.30	1						
*Platambus maculatus*										0.01	0.44	11			
*Laccophilus minutus*										0.08	0.46	15			
*Laccophilus hyalinus*	0	0.32	2				0.004	0.33	3	0.04	0.40	8			
*Oulimnius tuberculatus*										0.0002	0.40	8			
*Helochares lividus*										0.05	0.47	19			
*Peltodytes caesus*													0.03	0.40	8
*Hydrochus brevis*													0.09	0.40	10
*Hydroporus obscurus*													0.09	0.40	9
*Acilius canaliculatus*	0.001	0.34	6										0.10	0.40	8

The parameter that indicates the importance of species in a network in relation to their influence on other species is the node closeness centrality (NCC) (Table 4). The highest NCC values (from median to maximum) were found for the network in mesotrophic lakes (0.25–0.52). The most important species was eurybiont—*Noterus crassicornis* (0.52), followed by tyrphophiles—*Hydroporus tristis*, *Enochrus testaceus* (0.49), *Hydroporus angustatus* (0.47) and *Anacaena lutescens* (0.46). Rheophiles, such as *Ilybius fenestratus* (0.43), *Haliplus fluviatilis* (0.41) and *Hygrotus versicolor* (0.40) and argilophilous species—*Haliplus confinis* (0.42)—were also of great importance in this network. Similarly high NCC values were found in eutrophic lakes (0.22–0.51). The most important species were: *Hydrobius fuscipes* (0.51), *H. fluviatilis* (0.5), *H. confinis* (0.49) and *I. fenestratus* (0.47), *Bidessus hamulatus* (0.25). Rheobionts, such as *Potamonectes depressus* (0.30), were of great importance in this environment. (0.30). The range of NCC values for the clay pit network was narrower (0.22–0.48). The most important species were *Hydroporus neglectus* (0.48), *N. crassicornis* (0.46). Argilophiles—*Helophorus minutus* (0.46), *Laccobius minutus* (0.46), and rheophiles—*Ilybius fuliginosus* (0.36), *Porhydrus lineatus* (0.38), *Laccophilus hyalinus* (0.32) and *H. fluviatilis*, *H. fulvus*, *I. fenestratus* and *B. hamulatus* were also clearly important in this network.

Lower NCC values were found in gravel pits (0.17–0.45). The most important species were: *Hydroporus nigrita* (0.45), *Haliplus ruficollis* (0.44), *Coelambus impressopunctatus* (0.43). The lowest value for this centrality characteristic (0.20–0.40) was determined for dystrophic lakes. The most important species were eurybionts—*Agabus fuscipennis* and *Peltodytes caesus*, and tyrphophiles—*Hygrotus decoratus*, *Hydroporus obscurus* (0.40), *Acilius canaliculatus* and *Enochrus affinis* (0.39).

The contribution of individual species to the cohesion of an entire network can be measured with the metric of node betweenness centrality (NBC). The highest value (0.22) was found in the mesotrophic lake network for *Noterus crassicornis*, *Agabus fuscipennis* (0.12) and *Enochrus melanocephalus* (0.11). They made the greatest contribution to the cohesion of the network. This attribute favours the species (nodes) that connect to clusters (sub-networks) composed of other species. As a result, the network is less coherent and more fragmented. Species (nodes) that communicate with other clusters of the network play a more important role than those that are located within the sub–networks.

In the network of interspecific interactions developed for gravel pits, the highest NBC values were found for *Hydroporus nigrita* (0.14) and *Noterus clavicornis* (0.10). In the network of dystrophic lakes, the highest NBC values were found for *Hydrobius fuscipes* (0.12), *Hydroporus obscurus* (0.10), *Enochrus affinis* and *Anacaena lutescens* (0.09). Lower NBC values were found in other waters. The highest value (0.09) was found in eutrophic lakes network for *Haliplus fluviatilis* (0.10), *Hydrobius fuscipes* (0.09), in clay pits network—for *Hydroporus neglectus* (0.08), *Cercyon tristis* (0.07) and *Noterus clavicornis* (0.06). These networks also had the lowest number of species that are unimportant for the cohesion of the network (with the lowest NBC values) (Table 4).

An important indicator that describes the relationship between individual nodes (species) is the correlation coefficient (r) and the edge betweenness centrality (EBC) (Table 5). The EBC describes the number of shortest paths that lead through an edge of the graph. In our study, EBC means the importance of interactions between two taxa for the coherence of the entire biocoenosis network, i.e. the lower the value, the higher the correlated connections, high values (long edges mean interactions with lower correlations).

**Table 5:** Highest values of edge (relationships) betweenness centrality (EBC) and correlation (r) between beetle species in the studied water bodies.

Pair of species	*r*	EBC
Clay pits		
*Enochrus fuscipennis* and *Cymbiodyta marginella*	1.0	2.0
*Helophorus aequalis* and *Helophorus griseus*	0.88	7.75
*Enochrus isotae* and *Anacaena lutescens*	1.0	13.61
*Hydroporus planus* and *Hydroporus erythrocephalus*	1.0	15.73
*Scarodytes halensis* and *Helophorus nubilus*	1.0	20.11
*Haliplus fulvus* and *Bidessus hamulatus*	1.0	19.81
*Ilybius fenestratus* and *Enochrus melanocephalus*	0.19	335.36
*Graptodytes pictus* and *Haliplus fulvus*	0.21	249.5
*Laccobius atrocephalus* and *Hydroporus neglectus*	– 0.17	208.6
*Limnebius papposus* and *Graphoderus austriacus*	– 0.78	69.22
Gravel pits		
*Haliplus immaculatus* and *Laccophilus hyalinus*	1.0	2.0
*Coelambus confluens* and *Guignotus pusillus*	0.96	35.46
*Coelambus confluens* and *Helophorus granularis*	0.96	31.18
*Helophorus aequalis* and *Helophorus granularis*	0.95	31.40
*Agabus bipustulatus* and *Dytiscus marginalis*	0.57	270.0
*Dytiscus marginalis* and *Hygrotus versicolor*	0.57	270.0
*Scarodytes halensis* and *Dytiscus marginalis*	0.22	364.0
*Hydroporus fuscipennis* and *Hydroporus umbrosus*	– 0.83	21.47
*Haliplus ruficollis* and *Copelatus haemorrhoidalis*	– 0.62	22.29
Mesotrophic lakes		
*Hydaticus seminiger* and *Ilybius ater*	1.0	2.0
*Hydrochus ignicollis* and *Ilybius ater*	1.0	2.0
*Hydrochus elongatus* and *Ilybius ater*	1.0	2.0
*Laccobius biguttatus* and *Noterus crassicornis*	0.48	288
*Agabus undulatus* and *Noterus crassicornis*	0.19	190.7
*Hydaticus transversalis* and *Ilybius similis*	– 1.0	69.72
*Limnebius crinifer* and *Agabus undulatus*	– 0.65	108.04
Eutrophic lakes		
*Limnebius atomus* and *Hydrochus crenatus*	1.0	2.0
*Hydroglyphus pusillus* and *Oulimnius tuberculatus*	1.0	3.83
*Dytiscus marginalis* and *Hydroporus incognitus*	1.0	4
*Gyrinus substriatus* and *Laccophilus minutus*	0.24	364.1
*Limnebius crinifer* and *Helochares lividus*	– 0.74	38.13
*Helochares lividus* and *Ilybius ater*	– 0.66	43.49
*Haliplus confinis* and *Ilybius crassus*	– 0.62	67.25
*Dytiscus marginalis* and *Laccophilus hyalinus*	– 0.15	164.6
*Dytiscus circumcinctus* and *Hydrobius fuscipes*	– 0.53	314.8
Dystrophic lakes		
*Helophorus granularis* and *Helophorus griseus*	1.0	2.0
*Peltodytes caesus* and *Enochrus testaceus*	1.0	2.0
*Hydroporus incognitus* and *Hydroporus notatus*	1.0	2.0
*Haliplus ruficollis* and *Haliplus fulvicollis*	1.0	27.19
*Agabus congener* and *Rhantus pulverosus*	1.0	9.85
*Porhydrus lineatus* and *Laccophilus poecilus*	0.88	26.17
*Hydroporus tristis* and *Anacaena lutescens*	0.98	126.7
*Hydroporus obscurus* and *Anacaena lutescens*	0.72	190.06
*Gyrinus substriatus* and *Anacaena lutescens*	0.45	248.9
*Helophorus minutus* and *Gyrinus suffriani*	0.32	264.0
*Agabus congener* and *Hydrochus nitidicollis*	– 0.81	44.21

The largest ranges of EBC values were found in gravel pits and eutrophic lakes (Table 5). The highest number of lowest EBC values (2), illustrating the species pairs linked by the strongest relationships to maintain the network, were found in gravel pits (12). In the other environments, the number of such pairs was similar (4–6 species pairs). In most cases, correlations between species were very high (*r* = 1). In turn, the highest number of weakest correlations between species was found in gravel pits (10) and eutrophic lakes (9). Negative correlations were most frequent in eutrophic lakes (52), clay pits (52) and mesotrophic lakes (48). Selected species pairs and the highest values of betweenness centrality (EBC) and correlation (r) between these species in the analysed water bodies are listed in Table 5.

In the analysed networks, it is possible to indicate species with the highest NBC value that were also one of the “nodes” in the pair of species with the highest EBC. These were the pairs: *Hydroporus neglectus* (NBC = 0.08) with *Laccobius atrocephalus* (EBC = 208.6 in clay pits), *Laccobius minutus* (0.08) with *Gyrinus substriatus* (364.1 in eutrophic lakes), *Noterus crassicornis* (0.22) with *Laccobius biguttatus* (288) and *Agabus neglectus* (190.0) in mesotrophic lake and *Anacaena lutescens* with *Gyrinus substriatus* (248) and *Hydroporus tristis* (126.7) in dystrophic lakes. They can lead to the analysed network being split into two separate networks. This species cannot be identified in gravel pits (Tables 4 and 5).

### Migrations of beetles between different types of water bodies

Among the beetles collected, 25 species were found to occur in all habitats (Table 1). More species were found in eutrophic lakes and clay pits (72) and gravel pits (69), while the fewest species were found in dystrophic lakes and clay pits (51) and gravel pits (50). Common species were present in all the ecological elements analysed (Table 6).

**Table 6:** A general list of beetles that potentially migrate between the analysed aquatic body types (common for all water body types). *TCM* Total common species, *N* number of species, *L* rheophiles, *A* argilophiles, *T* tyrphophiles, *SW* mean Shapley values, *SW (sum)* sum of mean Shapley values, *SW (mean of sum)* Mean of sum of mean Shapley values.

Habitats	TCS (*N*)	L (*N*)	A (*N*)	T (*N*)	SW	SW (sum)	SW (mean of sum)
Clay pits–mesotrophic lakes	63	9	7	13	0.19–0.000	0.388	0.494
Mesotrophic lakes–clay pits					0.276–0.000	0.6	
Clay pits–eutrophic lakes	72	8	9	16	0.114–0.002	0.216	0.536
Eutrophic lakes–clay pits					0.216–0.008	0.856	
Clay pits–dystrophic lakes	51	2	4	15	0.300–0.000	0.952	0.539
Dystrophic lakes–clay pits					0.046–0.004	0.126	
Gravel pits–mesotrophic lakes	57	7	6	15	0.098–0.002	0.144	0.492
Mesotrophic lakes–gravel pits					0.646–0.002	0.84	
Gravel pits–eutrophic lakes	69	6	8	15	0.142– 0.002	0.178	0.601
Eutrophic lakes–gravel pits					0.566–0.006	1.024	
Gravel pits–dystrophic lakes	50	1	5	15	0.596–0.002	0.906	0.657
Dystrophic lakes–gravel pits					0.394–0.002	0.408	

**Fig. 6:**
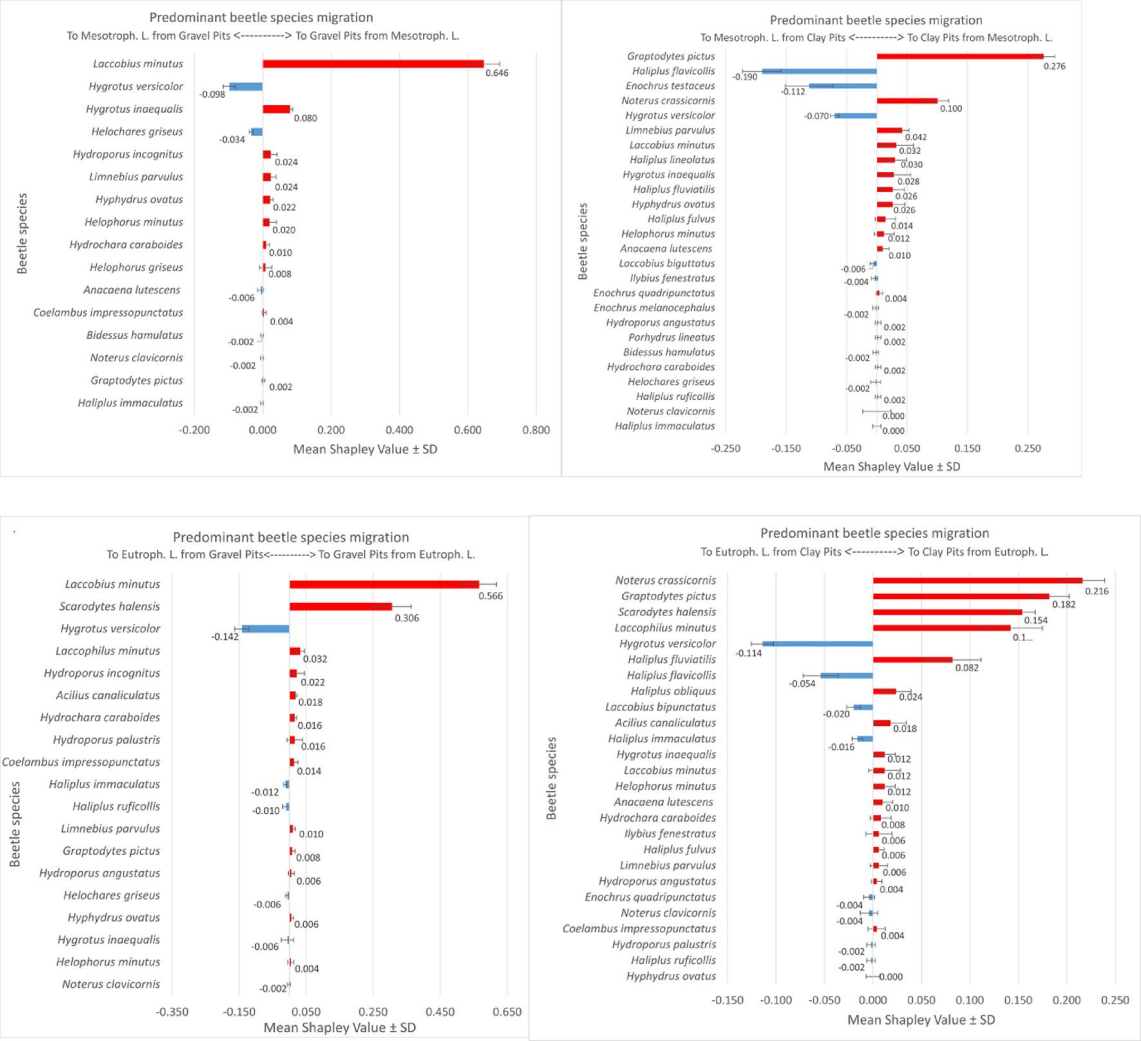

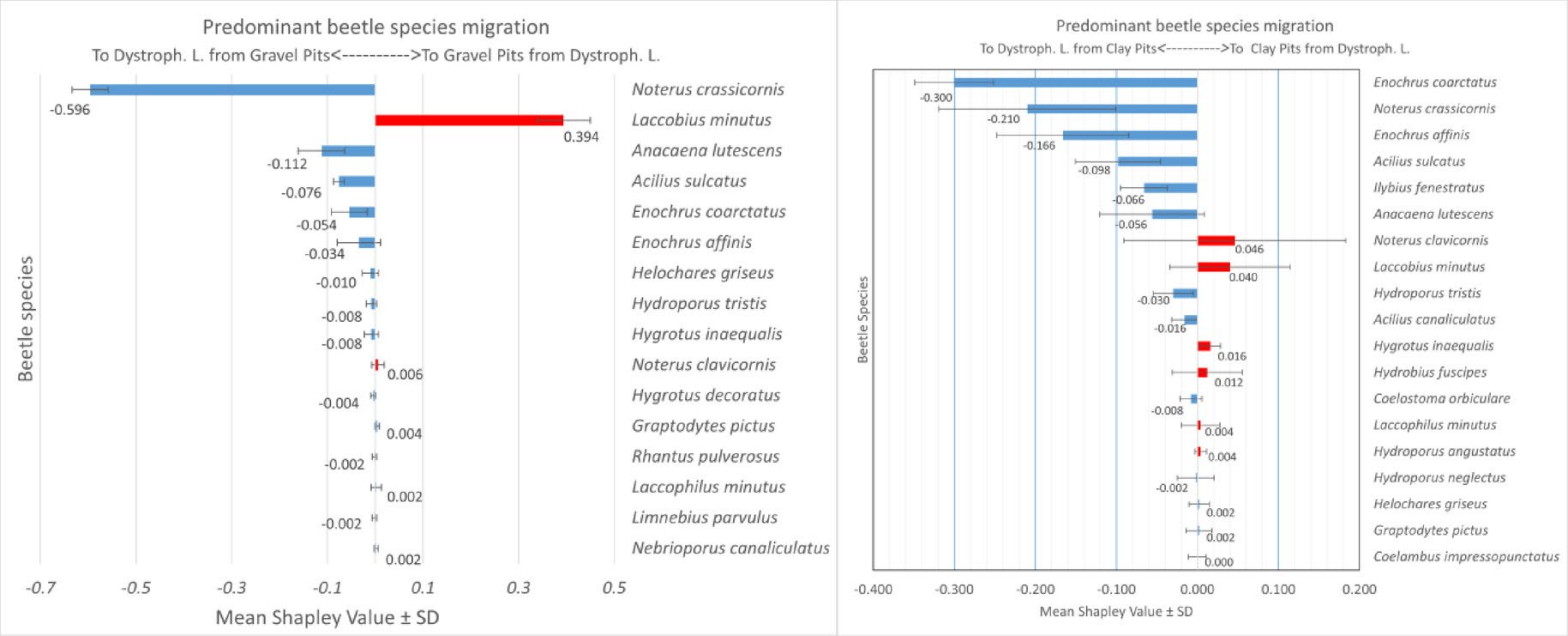
Predominant species migration between the analysed water bodies. Each of the six SHAP models shows the mean and standard deviation of five randomly selected individual SHAP modelling results. The values of the observations are shown: red bars and a positive Shapley value indicate a higher migration to pits from lakes than vice versa, blue bars and a negative Shapley value indicate a higher migration to lakes from pits than vice versa. The average accuracy of the XGBoost models was 78% for the training dataset and 70.1% for the test dataset. We obtained the Python code from https://github.com/dataman–git/codes_for_articles/blob/master/Explain%20your%20model%20with%20the%20SHAP%20values%20for%20article.ipynb. However, for basic SHAP modelling, we used the code template from the Towards Data Science portal (Prakhar Rathi, https://towardsdatascience.com/a–novel–approach–to–feature–importance–shapley–additive–explanations–d18af30fc21b) and the SHAP documentation (https://shap.readthedocs.io/en/latest/index.html.

Machine learning modelling results indicate different mechanisms of beetle migration between different types of aquatic environments. Shapley values different from “0” were most often achieved by species migrating between clay pits and mesotrophic and eutrophic lakes (26 species each). The fewest of these species (16) migrated between mesotrophic lakes and gravel pits (Fig. 6, Fig. S1–S6). We found the lowest Shapley value for all species in dystrophic lakes (graph–blue diagrams) and the highest Shapley value (graph–red diagrams)—in harmonic lakes, indicating an increase in biomass in certain lake types depending on the direction of migration. The sums of the mean Shapley values and the average of these values for different lakes/ponds combinations are shown in Table 6. They show the highest flow of beetle biomass between dystrophic lakes and gravel pits (0.657).

The species that migrates most frequently from all three lake types into the gravel pits is the argilophilous species *Laccobius minutus*, whose mean Shapley values are: 0.646 (for mesotrophic lakes), 0.566 (for eutrophic lakes) and 0.394 (for dystrophic lakes) (Fig. 6, Fig. S1–S6). This indicates a meaningful increase in the biomass of these species in gravel pits compared to the biomass reported from lakes. An important species migrating into gravel pits from eutrophic lakes is the argilophile—*Scarodytes halensis*, whose high mean Shapley values (0.306) indicate a higher biomass in gravel pits than in eutrophic lakes. The remaining species are characterised by a significantly lower mean Shapley value (0.002–0.08), with a higher biomass of eurytopic species, e.g. *Laccophilus minutus* (0.032) or *Hygrotus inaequalis*, which are less accompanied by tyrphophiles and argilophiles species compared to mesotrophic lakes (*Hydroporus incognitus*, *Helophorus griseus* and *H. minutus*) and eutrophic lakes (*Hydroporus incognitus*, *H. angustatus*, *Acilius canaliculatus* and *Helophorus minutus*).

A species with a different tendency, i.e. a clear migration from gravel pits to dystrophic lakes, is *Noterus crassicornis* (mean Shapley values = – 0.596). Tyrphophiles (mainly *Anacaena lutescens* (0.112), *Enochrus coarctatus* and *E. affinis*) also showed the same direction of migration. However, a rheophilous species—*Hygrotus versicolor*—migrates from gravel pits into eutrophic lakes.

The species that migrate from both eutrophic and mesotrophic lakes into clay pits are mainly eurybionts—*Noterus crassicornis* (mean Shapley’s values = 0.216 and 0.10 respectively) and *Graptodytes pictus* (0.18, 0.276). In addition to these two species, argilophiles from mesotrophic lakes also migrate to clay pits, in particular *Scarodytes halensis* (0.15) and *Laccobius minutus* (0.01 and 0.032), which reach a higher biomass in clay pits. Among the rheophiles species: *Haliplus flavicollis*, *H. versicolor*, *Ilybius fenestratus* and *Bidessus hamulatus* migrated from these water bodies to the lakes, while *Haliplus fluviatilis* and *H. fulvus* migrated from the lakes to the clay pits. Tyrphophiles migrate mainly from dystrophic lakes to clay pits, such as: *Enochrus coarctatus* (mean Shapley Valus = 0.300), *E. affinis*, *Acilius canaliculcatus* and *A. lutescens*. They are accompanied by species such as *N. crassicornis* and *I. fenestratus*, which represent different ecological groups. Migration from clay pits into dystrophic lakes is less pronounced and mainly comprises the species *N. clavicornis* and *L. minutus* (Fig. 6, Figs. S1–S6).

## Discussion

### The structure and functioning of the relationship network of beetle communities in the aquatic habitats studied

Many hydrobiologists point out that the ecological networks of macroinvertebrates, including beetles, inhabiting different water bodies are poorly understood so far^5,6,54–55^. The role of organisms influencing the extension of the network to the ecological landscape level is also unknown^21,36,56–59^. For this reason, we analyse rich material collected over a long research period (18 years) in the young glacial landscape (Masurian Lake District), including both lakes representing different stages of development and newly created post-exploitation water bodies. We were looking for confirmation that artificial water bodies are a potential substitute habitat for species threatened with extinction as a result of lake degradation and disappearance, and whether their presence actually has a positive impact on the conservation of biodiversity that guarantees the stability of the ecological landscape? We have found that the species richness of beetles in the selected habitats (169 species) accounts for almost half of the species reported for Poland, of which over 30 in artificial water bodies alone^89^. In addition, the beetle communities in certain types of water bodies differ both in terms of species and quantity (number and biomass). The ecological and functional structures of the analysed communities also vary. Additional analyses we conducted using an innovative tool^55,60−78^ in the form of graphs for structure analysis enabled a thorough assessment of the interactions between species in the relationship networks of the analysed beetle communities. They confirm clear differences in the structure of the beetle fauna of the studied environmental types, but above all they allow us to assess the role and importance of individual species in the structure, functioning and stabilisation of ecological networks.

We found the largest number of species (nodes) in relationship networks in eutrophic lakes, which is confirmed by other studies^39,57^ explaining the general eurytopic nature of beetles. However, we recorded slightly lower species richness in clay pits, confirming that smaller and shallower water bodies are particularly favoured and inhabited by beetles^24,35,44,45,58,90^. The high species richness in these environments leads to the largest number of interactions between species among all analysed networks and to a high value of network density. Both networks are characterised by the largest number of very high correlations between species and the highest number of shortest paths (Table 3) compared to other types of lakes or gravel pits, although the species important in the networks (forming their own sub-networks, clusters) are less correlated with each other, causing their moderate centralisation and considerable network heterogeneity. Among the species important to the eutrophic lake network (the highest NCC), only the pair *Haliplus fluviatilis* and *Hydrobius fuscipes* showed significant correlations. This co-occurrence may be due to different feeding preferences^21,55^. The periphery of the network is usually formed by large, predatory beetles, mainly eurybionts, sometimes accompanied by larger tyrphophiles, e.g. *Acilus canaliculatus*, *Colymbetes* sp., *Ilybius* sp. or *Agabus* sp. In the centre of the network there are a large number of coherent clusters (sub-networks) composed of ecologically more homogeneous groups of species, which clearly indicates the affinity of the different ecological groups to a particular habitat type: rheophiles prefer the presence of loose macrophytes (blue font) in the environment, argilophiles (red)—sandy bottom habitat, without macrophytes or tyrphophiles (green)—in an environment with a large amount of organic matter^57,91^ (see Fig. 4). For the cohesion of networks in eutrophic lakes, only smaller eurybionts seem to be important, which are the most numerous in the network, and to a lesser extent argilophiles (e.g. *Helophorus* sp., *Laccobius* sp. or *Scarodytes halenisis* and rheophile—*H. fluviatilis*. The central importance of organisms with low biomass in networks of trophic relationships is also confirmed by studies of the zooplankton of water bodies^81^. The interpenetration of clusters in the network and its low homogeneity are the result of the mosaic created by bare habitats with varying degrees of vegetation that coexist in the littoral lakes^57^.

A large number of weak negative correlations are based on predation in pairs of species that differ in body size: *Dytiscus marginalis* and *Laccophilus hyalinus*, *Agabus neglectus* and *Hygrotus inaequalis*, and positive relationships between large predatory species, e.g. tyrphophile—*Acilus canaliculatus* and eurybiont—*A. sulcatus*, which do not pose a threat to themselves, confirm that different environmental requirements, different microhabitats in which they occur and body size are of crucial importance for the co–occurrence of species^50^, ,^58,90–93^. In contrast, the network of clay pits is more homogeneous compared to eutrophic lakes. There are clear groups of species that represent the same ecological elements. These are mainly argilophiles species (*Coelembus confluens*, *Helophorus* sp, *Laccobius minutus*, *Nebrioporus canaliculatus*, *Scarodytes halensis*), whose biomass increases strongly in shallow, rapidly warming habitats on sandy bottoms. These are thermophilic alien species that can fly well and are the first colonisers of fresh water bodies after dredging^34,35,44,45^. For them, such water bodies are ecological corridors that allow them to expand their original range^21,34,35^. Separate groups are also formed by rheophilic species (*Hygrotus versicolor*, *Ilybius fenestratus*, *Ilybius fuliginosus*, *Porhydrus lineatus*, *Laccophilus hyalinus*, *Bidessus hamulatus* and *Haliplus fluviatilis*), which inhabit deeper habitats. Due to the numerous interactions between the species, they are an important part of the network. Some of them (*H. versicolor*, *I. fenestratus* and *H. fluviatilis*) are important for maintaining its consistency. According to Galewski and Tranda^94^, all these species are non-flying or poorly flying (e.g. *Haliplus fluviatilis*), so their presence in deep clay pits indicates stable conditions in these water bodies, which is also emphasised by Hansen and Kreiling^58^. Among the eurytopes, there are species associated with cleaner waters, e.g. *Noterus clavicornis*^94,95^. Therefore, despite a similar species richness, the fauna in clay pits is more specific, and their nature indicates significantly better water quality than in eutrophic lakes^34^.

Slightly fewer species create a network of relationships in gravel pits. Although there are the most direct and indirect connections between them (clustering coefficient), these are generally weak correlations compared to eutrophic lakes or clay pits. This leads to a thinning of the network. The most compact clusters are formed by argilophilous species, usually small, poorly swimming detritivores, e.g. *Laccobius*, *Helophorus*, *Enochrus*, with the exception of the deeper, well-swimming *Scarodytes halensis*, which occupies the peripheral part of the network. There are also clearly demarcated groups of small tyrphophiles, such as: *Hydroporus nigrita* and *Hygrotus decoratus*, which play the largest role in this network. This reconfirms the observations on the key role of low biomass organisms in interactive multidimensional ecological networks^81^. In contrast to the clay pits, the rheophiles organisms do not play a major role in the stability of the network. There is also little interaction between them.

In the network of relationships of mesotrophic lakes, we found the highest value of network density and the highest centralisation despite a significantly lower number of species, which means that the most important species for the network (the highest NCC) and those that play the most important role in its cohesion (NBC) are located in the centre of the network. In addition to the small eurytopic species, the tyrphophiles species are also of great importance here: *Enochrus coarctatus*, *E. melanocephalus* and *Hydroporus tristis*, as well as rheophiles species, in particular *Hygrotus vericolor*, *Ilybius fenestratus* and *Haliplus fluviatilis*. The latter species, which are considered flightless (a significant proportion of the population with severely reduced wings, deprived of the ability to fly), are particularly threatened by deteriorating conditions in natural lakes^40,57,58,93,94^. The remaining rheophiles and argilophiles are located at the periphery of the network and are generally not important for the coherence of the network. The relatively low number of very high correlations between species and low number of shortest paths (Table 3), especially those communicating with the largest number of species (characteristic path length), led to the greatest dilution of the network (higher network heterogeneity value) compared to the previous waterbody types. A larger psammolitoral zone, less dense vegetation creating intermittent, sometimes localised patches, and greater pressure from predatory fish are the reasons for the lower density and species richness of beetles. At the same time, the great importance of certain elements, especially in lake and rivers, emphasises the good quality of the water^40^.

Very few species form a network of relationships in dystrophic lakes, which is consistent with the common opinion that species diversity is low here^27–31^. However, the effect of frequent interactions between them is the lowest dilution of the network (lowest value of network heterogeneity) compared to the previous water types. A clear, homogeneous cluster is formed by groups of small tyrphophiles, of which the most important for maintaining the cohesion of the network (*Enochrus affinis*, *Hydroporus obscurus*) occur in its central part. These species are usually found in the hydrated *Sphagnum* mat that overgrows the littoral of the lakes. This co–occurrence results not only from habitat preferences, but also from a rich food base, a dense spatial structure of the microhabitat (high fractal dimension) that guarantees protection, limits competition and the absence of fish in the lake^27,41,58^. In the analysed networks, the species important for their stabilisation most often show positive relationships with each other, which prevents the fragmentation of the network and may indicate the maturity of the ecosystems^79^.

### Migrations of beetles between different types of water bodies

Trophic relationships between species, which take place in food chains that extend far beyond the boundaries of individual ecosystems thanks to the migration of organisms, contribute to functional integration at the level of the ecological landscape. Among the species that are most important in this respect are those that exhibit large dispersal. To identify them, we used ensemble XGBoost–SHAP modelling, an innovative tool that is increasingly used in ecological research to predict many complex natural phenomena^81,86^. The interpretation of the direction and intensity of insect migration based on Shapley values^87^ included in this work is, to our knowledge, the first such attempt in the world literature. This gave us the opportunity to perform a more in-depth analysis and assessment of the strength and direction of beetle migration between lakes and water bodies after excavation.

A general characteristic of aquatic beetles is their high migratory ability, especially in the eurybionts, including *Noterus crassicornic*, which dominates in our material. Therefore, they tend to be most numerous in all types of water bodies, travelling a migratory route of up to 20 km^56,94^. In our study, we found the most abundant species in eutrophic lakes and in gravel and clay pits, where the overall species diversity is the highest, which is consistent with previous studies^35,44,45,57,58,90^. However, not all common species undertake migrations, as evidenced by a much smaller (three times) number of species for which the Shapley values were different from zero. According to Barnes^50^ and Matsushima and Yokoi^56^, smaller water bodies are favoured by species that can fly well and find food and a place to lay eggs here, but in winter they certainly return to deeper lakes.

In order to maintain the functional integration that ensures ecological stabilisation across the landscape, the most important species are those with the highest dispersal, which also play an important role in shaping the structure and stability of the network of relationships in the analysed types of water bodies (high NCC, NDC and NBC values). In our study, these were different species representing different ecological groups.

Analysing the migration directions of the beetle between the water types reveals a greater affinity of the beetle fauna living in gravel pits to dystrophic lakes. It is noticeable that migrations from gravel pits to dystrophic lake are significantly more frequent than vice versa, which is indicated by the highest Shapley values. The nature of organisms should be demonstrated by the most specialised ecological groups^40,91^. Among the specialised migrants that more readily invade dystrophic lakes, the most numerous are tyrphophiles (*Anacaena lutescens*, *Enochrus affinis*, *E. coarctatus*), which play an important role in the relational networks of dystrophic lakes, as shown by the high attribute values of the nodes.

In the opposite direction, an argilophilous species—*Laccobius minutus*—migrates into gravel pits and reaches the highest numbers here. This species is also the most numerous immigrant from mesotrophic and eutrophic lakes, where it inhabits shallow, rapidly warming psammolitoral habitats. The second important specialised species is *Scarodytes halensis*, which clearly migrates from harmonic lakes towards anthropogenic water bodies, both gravel pits and clay pits. They are particularly abundant in these environments, especially in newly created ones. The species mentioned are thermophiles associated with waters of increased mineralisation linked to the Mediterranean region^35,94^. At the same time, many argilophilous species have an affinity for acidic waters (acidophiles), which are numerous in the hydrated *Sphagnum* mats surrounding dystrophic lakes^32,41^. According to Barnes^50^, these species are the first colonisers of newly created water bodies, which is undoubtedly related to their high mobility.

The proportion of the element most specific to lakes – rheophilous species that favour clean lakes—is also of great importance. There is a clear tendency to migrate into harmonious lakes (higher mean Shapley values) from two directions: *Hygrotus versicolor* migrates from gravel pits, and both *Hygrotus verisolor* and *Haliplus flavicollis* migrate from clay pits. *Haliplus flavicollis* tends to occur in mesotrophic lakes, while *H. verisolor* is found in eutrophic lakes. Other rheophiles: *H. fluviatilis*, *H.* and *Ilybius fenestratus*—migrate in the opposite direction—to clay pits. The species mentioned also have high attributes of nodes in the networks of ecological relationships, both in harmonic lakes and in clay pits.

According to Hansen and Kreiling^58^, Haliplidae, with the exception of *H. fulvus*, have low flight and dispersal ability and prefer larger, more stable aquatic environments, in contrast to most Dytiscidae (e.g. *H. versicolor* or *I. fenestratus*). Therefore, we can confirm that the flow of beetle biomass between harmonic lakes and deep anthropogenic water bodies may indicate that the inability to fly affects only a proportion of individuals in the population and that the environmental conditions in these water bodies are optimal for life and most likely also for overwintering^96^. Undoubtedly, the occurrence of such specialised species in anthropogenic water bodies (which are important for the stabilisation of the network) indicates not only good water quality, but also the stabilisation of environmental conditions here, which may be affected by climate warming^81^. Therefore, clay pits, especially large and deeper ones, can be a substitute habitat for typical lake species from harmonic lakes, while gravel pits – for species from smaller dystrophic lakes. At the same time, they are ecological corridors for alien, thermophilic species that extend the boundaries of their original range. Therefore, these water bodies are extremely important from an ecological point of view, as they stabilise the dynamic balance in ecological landscapes and serve as sites of occurrence for species that stabilise ecological networks.

## Conclusions

In our study, we analysed the relationships between the species of beetle communities inhabiting lakes and post-exploitation water bodies and determined the importance of individual species in these networks. The measurement parameter in our analyses was biomass. In addition, we used machine learning algorithms the Extreme Gradient Boosting (XGBoost) and the SHapley Additive exPlaination (SHAP) to identify species that are particularly important in migrations between water bodies and to assess the direction and strength of migrations based on the Shapley value. We have shown that the studied networks of connections between species in different ecosystems differ significantly in terms of cohesion, density, network centralisation and heterogeneity, which may be related to their spatial structure. All networks are dominated by positive mutual relationships between beetle species. We identified species that are particularly important for the stability of the networks. Such networks can be divided into clusters (subnetworks) consisting of species with similar habitat and food preferences, which usually occupy a central place in the networks, and the species with the lowest biomass are the most important. Innovative methods - graph network analysis and explainable machine learning models – have significantly expanded existing knowledge on the relationships between species in beetle communities in the context of natural and anthropogenic changes to ecological landscapes. Species that migrate most frequently and simultaneously have the highest values of network attributes (e.g. *Scarodytes hanensis*, *Laccobius minutus*, *Hygrotus versicolor*, *Ilybius fenestratus*, *Haliplus fluviatilis*) are most important as stabilisers of the network of relationships in the ecological landscape.

## Materials and methods

### Study area

**Fig. 7:**
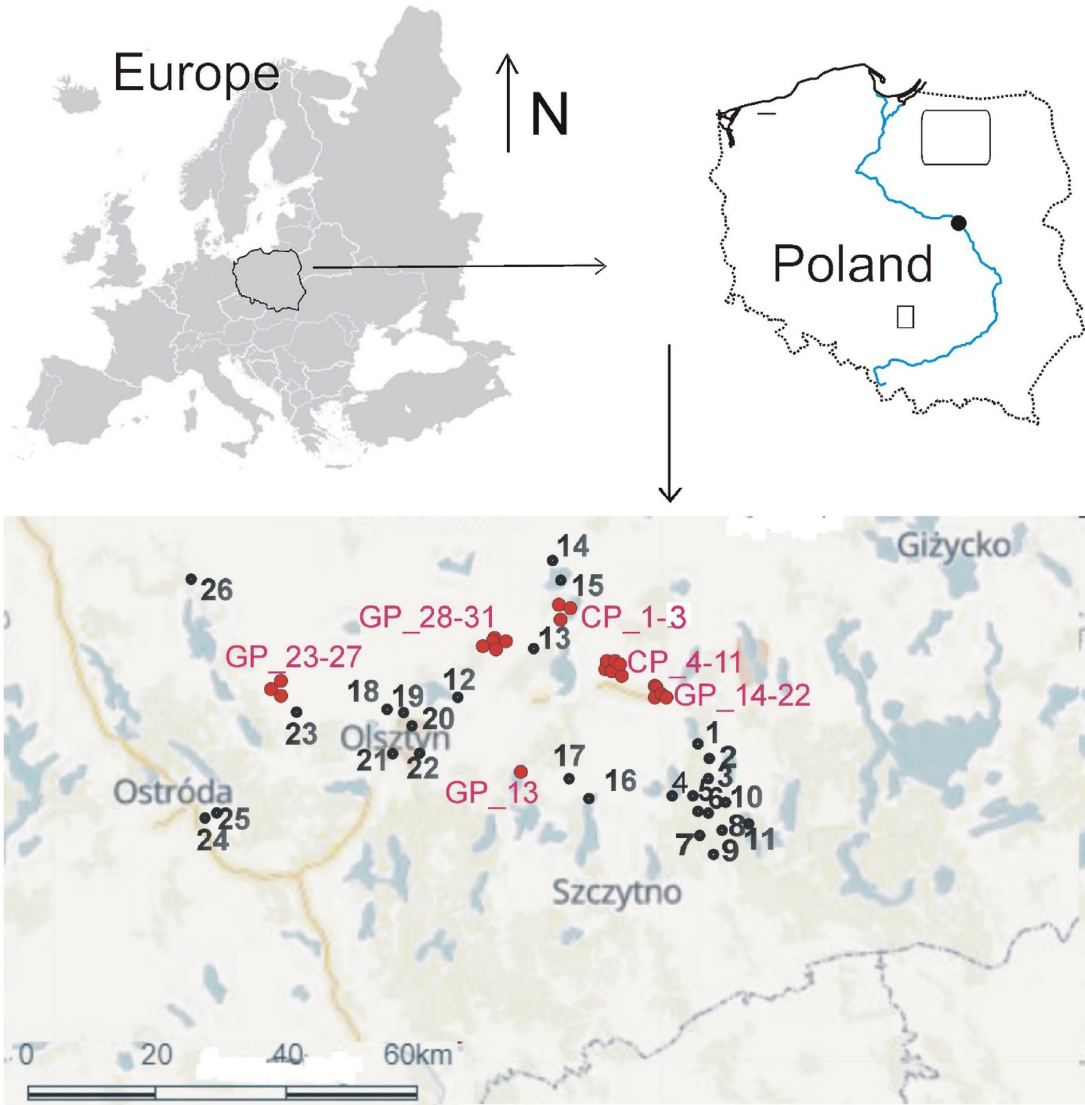
Study area. Location of sampling sites in lakes (1–26 - black fonts), 1—Babięty, 2—Majcz Wielki, 3—Bobrówko, 4—Borkowskie, 5—Gryżewskie, 6—Krucze Oko 7—Kruczek Duży, 8—Kruczek Mały, 9—Kruczy Staw, 10—Skarp, 11 - Klimunt, 12 - Dobrąg, 13—Białe, 14—Luterskie, 15—Luterskie 1, 16—Kociołek, 17—Kośno, 18—Redykajny, 19—Tyrsko, 20—Długie, 21—Kortowskie, 22—Skanda, 23—Jonkowo, 24—Motylek, 25 Żabie, 26—Wukśniki; and clay pits (CP_1–11) and gravel pits (GP_12–31) (red fonts).

We studied 56 water bodies – 25 lakes and 31 anthropogenic water bodies – in northern Poland, in the Masurian Lake District (Fig. 7, Table S2). The studied lakes differed in their surface area, depth, degree of development and differentiation of the littoral zone, physical and chemical parameters of the water and characteristics of the catchment area (forest, open). They represent different stages of lake evolution, both harmonic (from oligotrophic ones, low in fertility, through mesotrophic ones, to highly eutrophic lakes) and disharmonic (from oligohumic to polyhumic lakes).

**Fig. 8:**
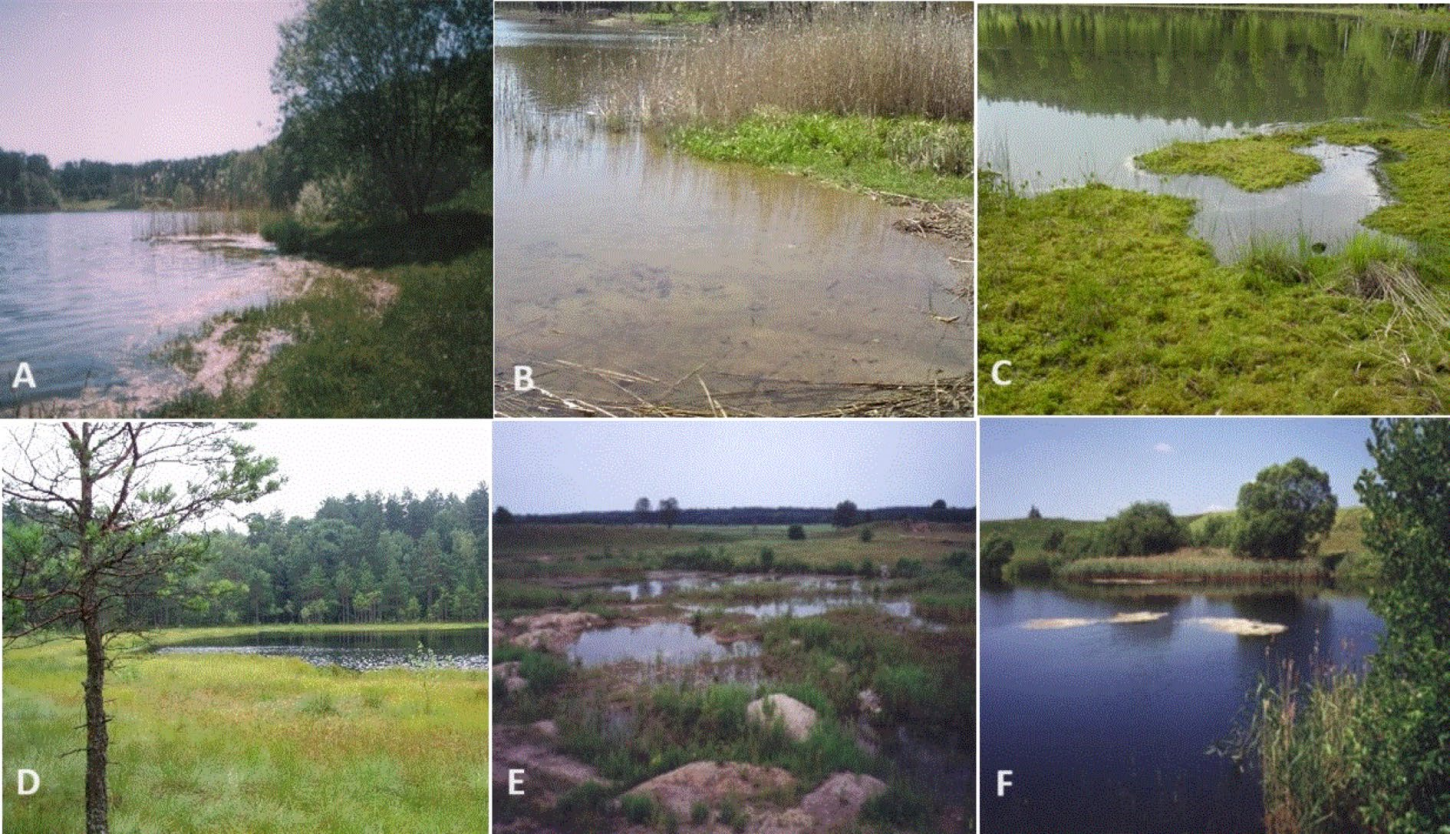
Study area: A—mesotrophic lake; B—eutrophic lake; C—*Sphagnum* mat in dystrophic lake; D—dystrophic lake; E—gravel pits, F—clay pit.

Therefore, we have applied the a priori typology proposed for the classical lake to three groups: mesotrophic lakes (5 lakes), eutrophic lakes (6) and dystrophic lakes (10)^25^ (Fig. 8).

Mesotrophic lakes with low fertility are characterised by blue–green water and a narrow, underdeveloped belt of phytolittoral vegetation consisting mainly of *Juncus bulbosus*, *Eleocharis palustris*, *Phragmites australis*, *Typha angustifolia*, *Typha latifolia* and *Isoëtes lacustris*. Eutrophic, more fertile lakes are characterised by a yellow–green colour and a well–developed littoral zone dominated by reeds, with accompanying communities of *Acorus calamus*, *Sparganium ramosum* and *Glyceria maxima*. These lakes represent different stages of harmonic development, and a clear determinant of the degree of succession is the degree of overgrowth of *Phragmites australis* on the littoral zone^97^. A separate group are dystrophic, humic lakes, represent different stages of disharmonic development, which are usually located in the forest and are characterised by reduced surface area and depth, low pH, low primary production and trophic activity, and a high content of humic acids, which give the water a brownish colour that limits the access of light^30^. According to common opinion, they are usually characterised by low species diversity^98–100^. The presence of *Sphagnum* mat in the littoral zone, which are characteristic of these lakes (their width, length and compactness), can be a determinant of succession and indicate the ecological age of the lake^26^.

The post-exploitation water bodies were in turn divided a priori into two groups: clay pits (10) and gravel pits (21), based on the mineral nature of their substrate^35^. Compared to lakes, they have a small surface area (from 30 m^2^ to 1 ha) and depth (0.5 to 10 m). They are also characterised by a different degree of development of aquatic vegetation (density and species diversity), which indicates different stages of their succession (from young water bodies without macrophytes to older ones gradually covered by rush plants such as *Carex* sp., *Juncus* sp., *Heleocharis* sp., *Glyceria maxima*, *Scirpus silvaticus*, *Hydrocharis morsus*–*ranae*, *Alisma plantago–aquatica* and others, scattered bulrushes, mostly *Typha latifolia*, nympheids: *Lemna minor*, *L. trisulca*, *Potamogeton natans* and elodeids: *Elodea canadensis*, *Ceratophyllum demersum*, *P. perfoliatum*, *P. obtusifolius*, *P. lucens*; to old—densely overgrown with *Phragmites australis*.

### Field studies and collecting samples

The studies were carried out from 1998 to 2014, in spring, summer and autumn. The faunal samples were collected with a dip net on an area of about 1 m^2^. In the pressed *Sphagnum* mat of the dystrophic lakes, 10 subsamples were taken with a 0.1 m^2^ sieve. The sampling sites were chosen to represent the greatest possible diversity of littoral habitats and areas of the individual water bodies. Four different littoral components (habitats) were thus identified: (1) arena zone (sandy bottom habitats), (2) diffuse macrophyte zone, (3) dense macrophyte zone and (4) Sphagnum mat and ecotone zones between land and water—in dystrophic lakes). Vegetation cover was assessed using the phytosociological records of Braun–Blanquet^101^. We calculated the area of *Sphagnum* mat in a lake in GIS using ArcGIS software (for Desktop 9.3.1., ESRI, Poland). The sites were mapped using the data available in WMS format in Geoportal 2.

All water bodies were characterised in terms of area and percentage of individual habitats in the littoral zone (1–4) (Table S2). Faunal samples were taken from three depths: (1) shallow, in the ecotone zone—5–10 cm, (2) deeper 10–30 cm and 3) > 40 cm deep. A total of 1096 samples were taken and subsequently described on the basis of selected environmental parameters (Table 1). The wet biomass of all collected individuals was measured using a Radwag AS 160.X2 PLUS analytical balance. The characteristics of a particular parameter were described using qualitative values, where a rank corresponds to the strength of a particular value. The water parameters, i.e. temperature, pH, electrolytic conductivity and saturation content, were measured using an Elmetron CX–401 multiparametric sampling probe (Elmetron, Poland). The values for the analysed variables have already been presented in previous publications^35,39–41^.

### Ecological and statistical analyses

Species diversity was calculated as follows: S—number of species, N—number of individuals and D—percentage. Five ecological groups were distinguished to determine the holistic character of the fauna^91^. These were: eurybionts (species that prefer small and highly eutrophic waters), rheophiles (lake and river species—species typical of less eutrophic waters; found mainly in clean lakes and river ponds, rheobionts (species commonly found in river courses), psammophiles (associated with water bodies with increased mineralisation and showing a higher preference for unvegetated environments with sandy bottoms) and tyrphophiles (species characteristic of different small, polyhumic waters).

We used the non–parametric Kruskal–Wallis test to detect significant statistical differences in species diversity, abundance and biomass of lake beetles in the different water types. We used the same test to indicate significant statistical differences in species diversity, abundance and biomass of the different ecological groups in the habitats analysed. Significant results were tested for pairwise comparisons with a post–hoc test for multiple comparisons of mean ranks for all samples. We performed the Kruskal–Wallis test in Statistica, ver. 13.3 (StatSoft, Tulsa, USA).

### Graph network analysis

One of the features of graph theory is the ability to evaluate the properties of the whole network as well as the attributes of nodes and edges in terms of their centrality measure in the whole network^102^. In our work, we analysed the properties of the water beetle community network in the studied habitats: gravel and clay pits and mesotrophic, eutrophic and dystrophic lakes. Graph theory was also used to analyse the role of individual species in these networks and the relationships between them.

The five graphs created for the above habitats were analysed using the Cytoscape package (http://www.cytoscape.org/) with the MetScape and NetworkAnalyzer applications for network metrics calculation, where the networks were based on partial correlations between beetle species wet biomass. In the databases, the taxonomic units of the beetles are listed in the columns and their biomass measurements in mg in the rows. The biomass values were normalised by autoscaling. The partial correlation matrix was calculated using Correlation Calculator 1.01 (University of Michigan). Networks were created based on partial correlations between nodes that had a statistical significance coefficient of *p* ≤ 0.05 for the sample sizes in each habitat. As the algorithm for visualising the graph, i.e. the arrangement of nodes and edges on the graph plan, we used the edge-weighted, spring-embedded layout^103^ with Pearson correlations as weights and a heuristic interpretation of the weight values. The absolute values of the correlation coefficients between the nodes were used as weights.

Beetle networks in five types of water bodies were characterised using basic network attributes: number of neighbours, shortest path, characteristic path length, clustering coefficient, network centralisation, network density and network heterogeneity^79,102^. We used three primary node centrality attributes: Node Degree Centrality (NDC)^103^, Node Closeness Centrality (NCC)^105^ and Node Betweenness Centrality (NBC)^84^. The sign and strength of the correlation between nodes and Edge Betweenness Centrality (EBC) were also analysed^106^. The papers cited above contain mathematical definitions of the attributes mentioned. For the purposes of this study, however, they were interpreted ecologically. Accordingly, NDC indicates the number of connections of a species with other species, while NCC is a measure of the speed of information dissemination from a particular taxon to other (also indirectly) connected species in the network. The role in maintaining the unity of the whole network is indicated by NBC, which measures the importance of a particular taxon for the cohesion of the network, and EBC, which refers to the importance of the connections between taxa for the integrity of the beetle species network. The highest values of NBC and ECB refer to the situation where the analysed network can be split into two separate networks^79^.

### Machine learning modelling

Our basic assumption is that the modelling is based on the mutual influence of the beetle species of the studied types of water bodies and the relationship between the variability of their biomass. To predict multiple combinations of taxon systems in mutual relationships, we used the boosting technique of the eXtreme Gradient Boosting algorithm (XGBoost). The XGBoost models were then used to analyse the migration of beetle species between different types of water bodies, with the aim of evaluating the advantage of migrating from one type of water bodies to another. For this purpose, the SHapley Additive exPlanations (SHAP) algorithm was used, which belongs to the group of machine learning explanatory algorithms^107^ and originates from the mathematical theory of corporate games^108^. The SHAP^107^ algorithm is based on Shapley’s concept of value as part of mathematical game theory and its branch describing co-operative games^63^. This modelling can be used to predict the local importance of variables^108^. If we consider the interactions between species in a bioceonosis as a game for resources and, more broadly, for adaptation and survival^109^, modelling with SHAP can be successfully used as a tool to predict and determine trends in changes in the living components of an ecosystem under the influence of variations in environmental factors^81^. In the present study, the Shapley value was used to predict migration trends of aquatic beetle species from lakes to pits and vice versa based on the collected material (Table 1).

We created six migration paths with pairs of aquatic habitat types: gravel pits—mesotrophic lakes, clay pits—mesotrophic lakes, gravel pits—eutrophic lakes, clay pits—eutrophic lakes, gravel pits—dystriophic lakes and clay pits—dystrophic lakes. Two classes were formed for each pair: Class “0” for lakes and Class “1” for pits. The six migration paths studied were based on six databases containing the beetle species occurring in the corresponding habitat type in the columns and the measurements of their biomass (in mg) in the rows.

We assumed that in the case of a lake-type beetle community, the stake in the game (“gain”) is migration to the pit-type reservoir (Class “1”) and the “loss” is migration in the opposite direction (Class “0”). The response of a particular taxon to a specific direction of migration, from lake to pit or from pit to lake, may take the form of a response by a tendency to:

- increase the biomass of the species migrating from the lake to the pit to different degrees relative to the biomass of the species migration from the pit to the lake (mean Shapley value is positive),
- increase the biomass of the species migrating from the pit to the lake to varying degrees in relation to the biomass of the species migration from the lake to the pit (mean Shapley value is negative),
- does not respond to migration in both directions (mean Shapley value is 0).

In order to predict the migration of beetle species between lakes and pits, we created a sequence diagram to prepare the data for modelling and the creation of subsequent models. Converting each of the six output data tables and fitting them to the modelling with machine learning tools consisted of changing the Excel file to a text file with a csv extension. After splitting the data into train and test subsamples in a 70 to 30% ratio, we submitted the data for modelling using the XGBoost and SHAP algorithms. To increase confidence in the modelling results, 5 XGBoost and SHAP models were randomly run and the mean and standard deviation of the predictions of these five models were reported as the final modelling results. The XGBoost models were checked for accuracy and overfitting. All data mining and modelling elements were performed in Python 3.8 using the Jupiter Notebook programming environment.

We used the Extreme Gradient Boosting algorithm as an introductory model for SHAP modelling to evaluate the prediction of migration of beetle species from lakes to pit water bodies and vice versa. The hyperparemeters of the model were as follows: n_estimators = 1000, max_depth = 10, learning_rate = 0.001. We extracted a code from the Kaggle notebook “Ensembles and Model Stacking” (Eshaan Kirpal (2019): https://www.kaggle.com/eshaan90/ensembles–and–model–stacking). The SHAP algorithm has the property that the Shapley value plot for features in the model contains the individual location of these values for each observation. To synthesise the predominant positive (lake-pit migration) or negative (pit-lake migration) change in a feature, i.e. the biomass of a beetle taxon, we used the ABS_SHAP function. We obtained the Python code from https://github.com/dataman-git/codes_for_articles/blob/master/ExplainyourmodelwiththeSHAPvaluesforarticle.ipynb. However, for basic SHAP modelling, we used the code template from the Towards Data Science portal (Prakhar Rathi, https://towardsdatascience.com/a–novel–approach–to–feature–importance–shapley–additive–explanations–d18af30fc21b) and the SHAP documentation (https://shap.readthedocs.io/en/latest/index.html).

## Electronic supplementary material

Below is the link to the electronic supplementary material.

Supplementary Material 1

Supplementary Material 2

Supplementary Material 3

## Data Availability

All data generated or analysed during this study are included in this published article [and its supplementary information files].
