# Supplementary material for: Water beetle networks differences and migration between natural lakes and post-exploitation water bodies
Source: Sci Rep. 2025 May 7;15:15898. doi: 10.1038/s41598-025-00525-1 (PMC12059192; doi:10.1038/s41598-025-00525-1)
Supplement: Supplementary file 1 — Supplementary Material 1 [file 41598_2025_525_MOESM1_ESM.docx]

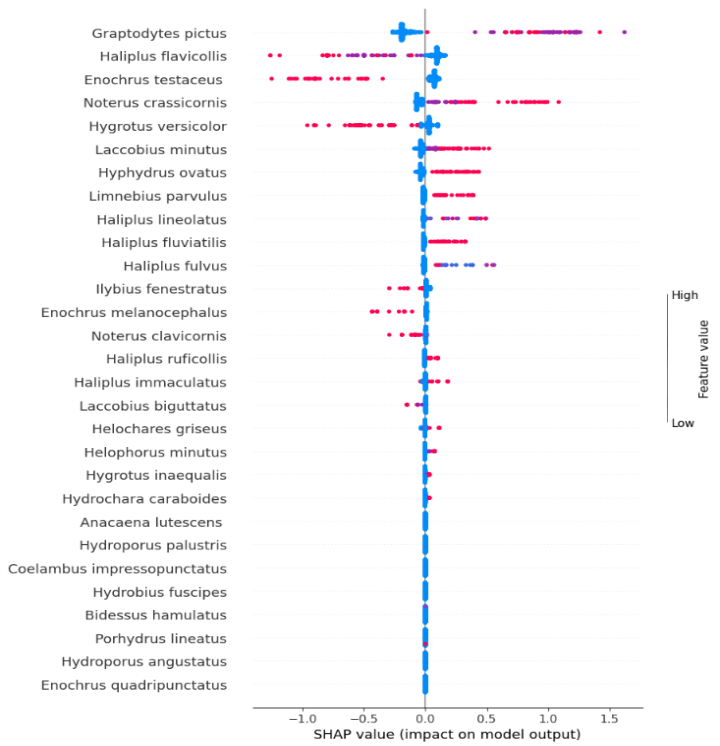

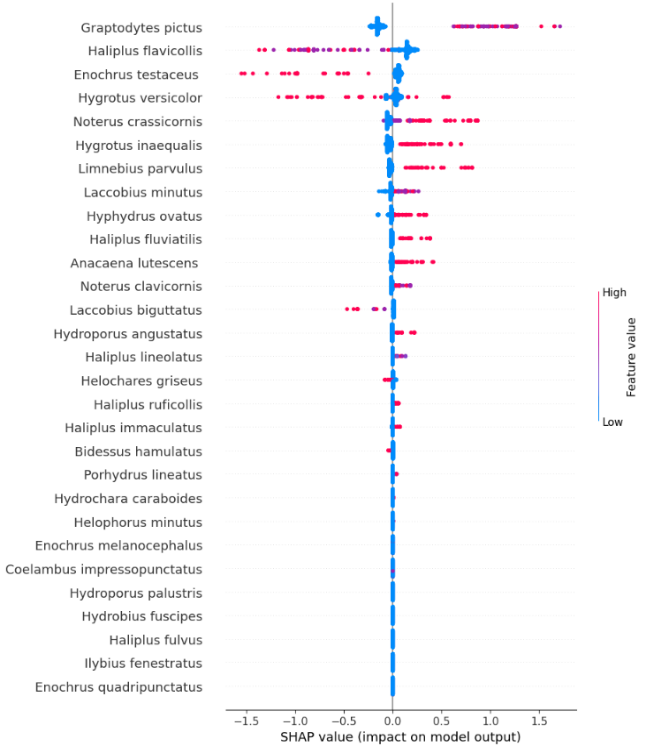

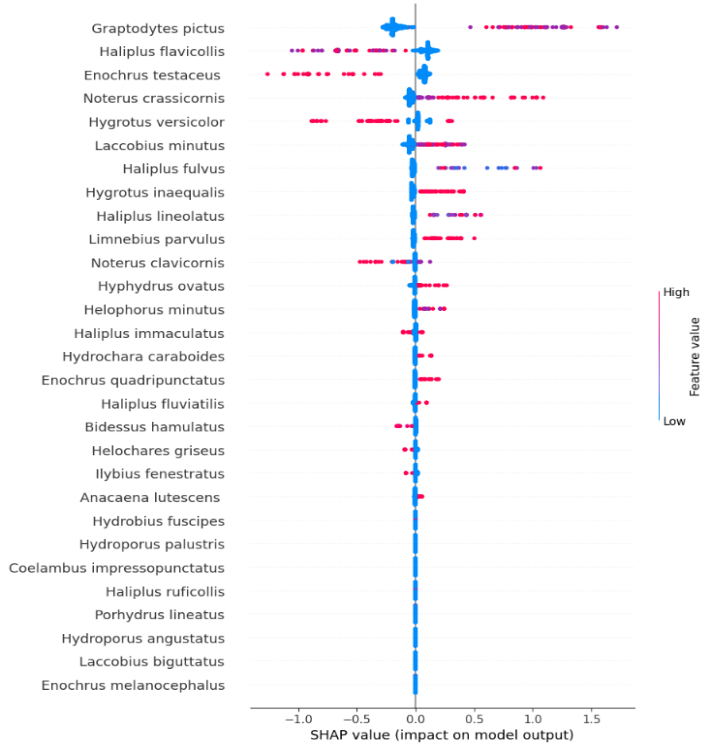

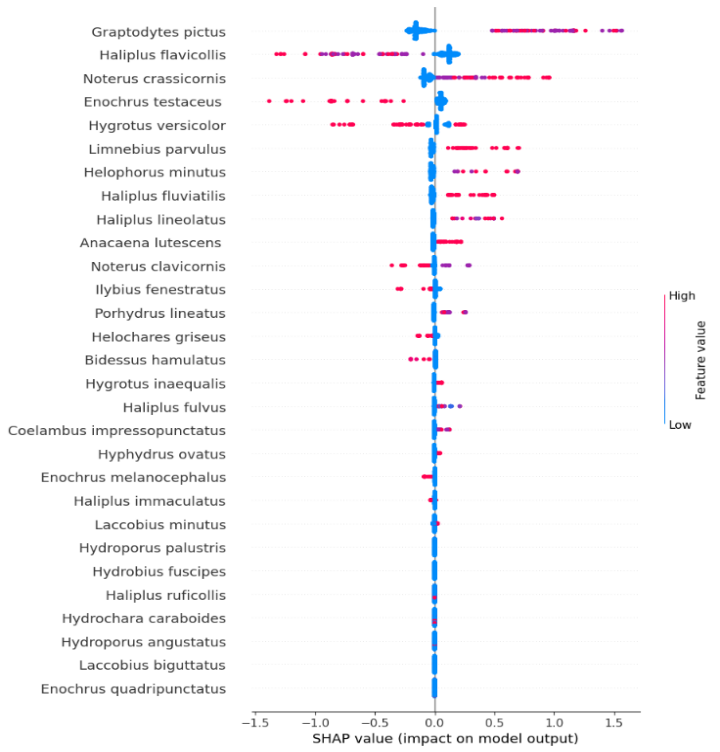


**3**

**4**

**2**

**1**


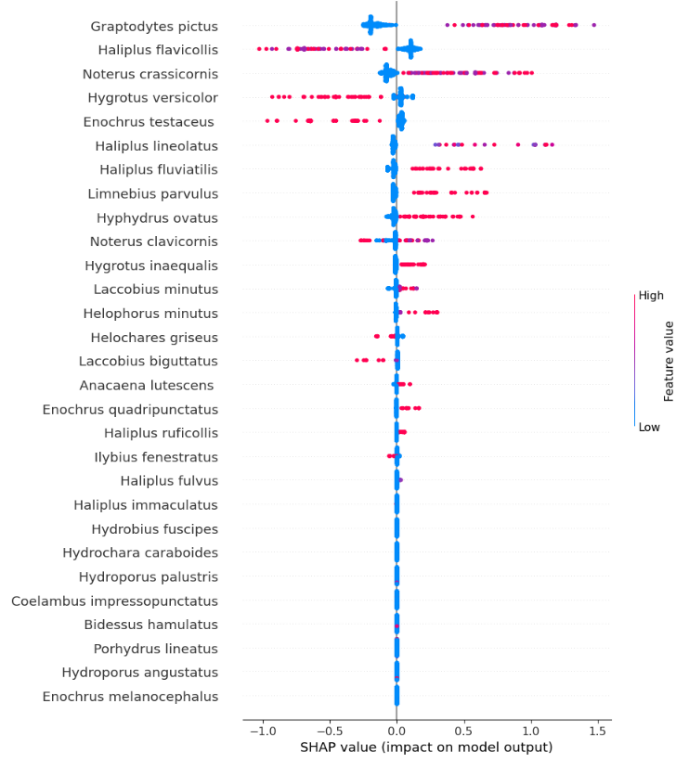


**5**


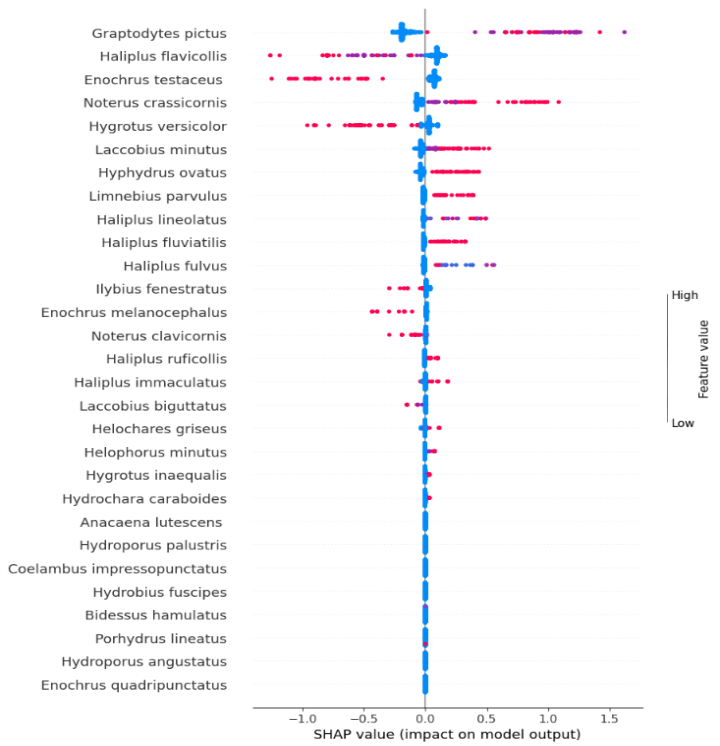

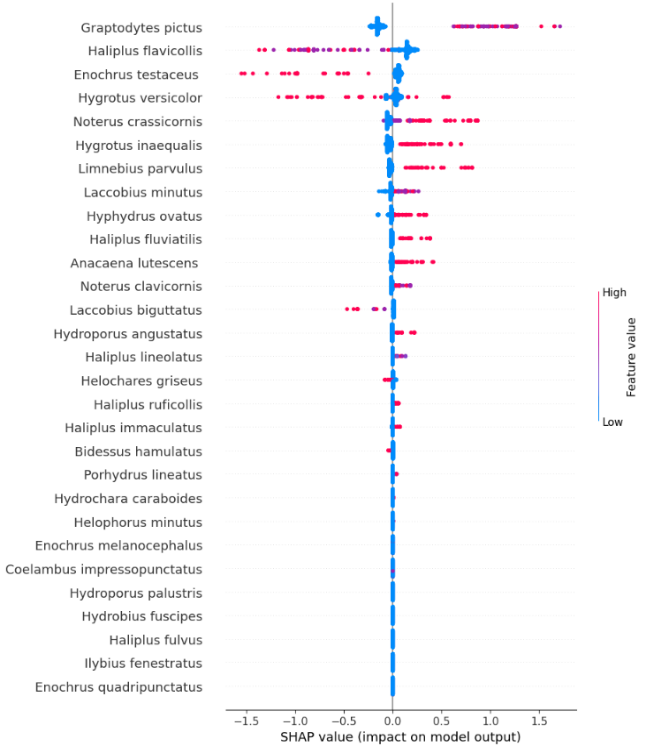

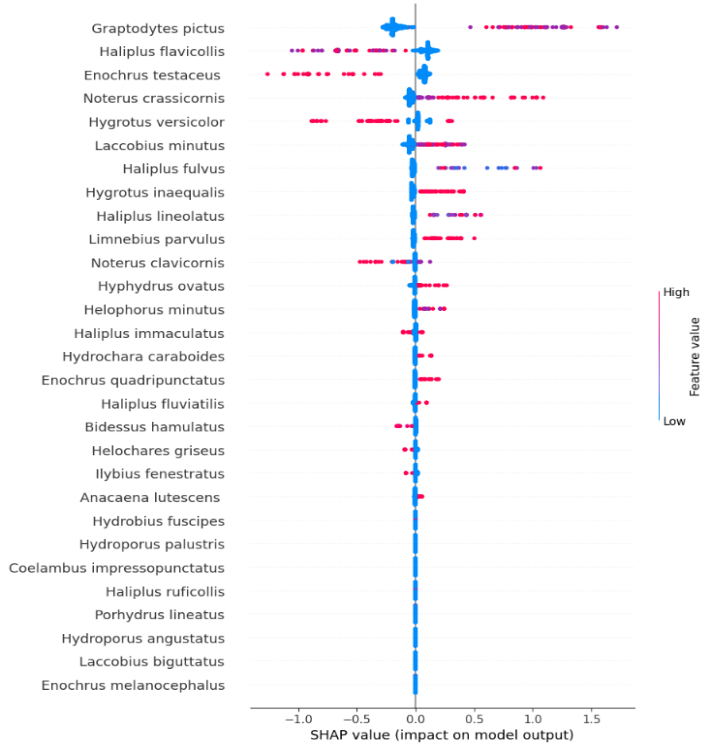

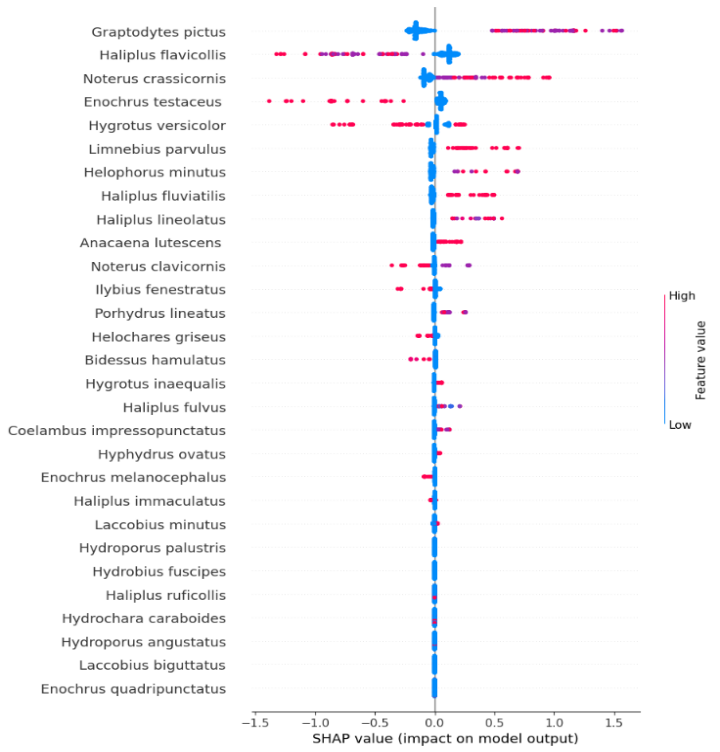


**3**

**4**

**2**

**1**


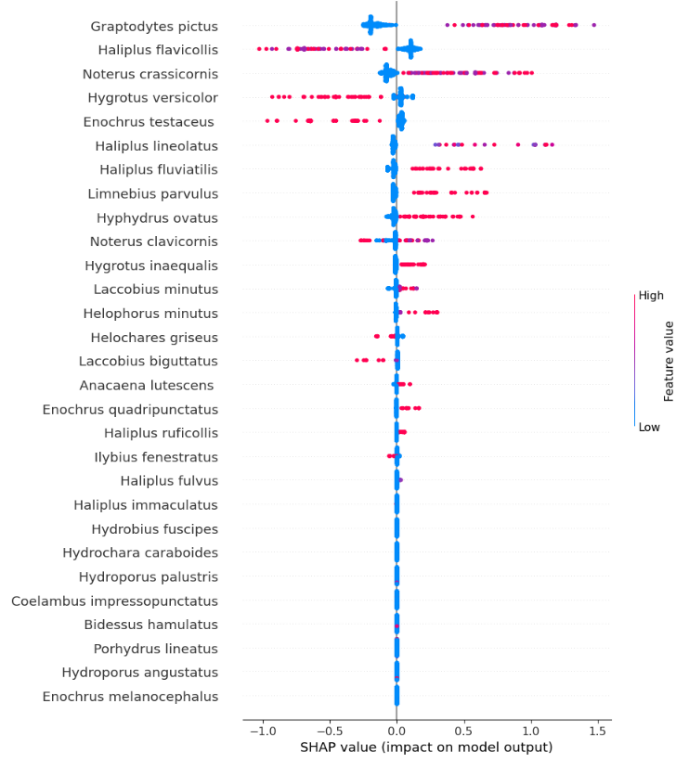


**5**

Fig. S1. Distribution of species (variables at y axis) migration between the mesotrophic lakes and clay pits. Each of the five SHAP models (1 – 5) shows randomly selected individual SHAP modelling results. The values of the observations are shown: red dots indicates higher value of variable and blue dots lower value. Positive Shapley value indicate migration to pits from lakes and negative Shapley value indicate migration to lakes from pits.


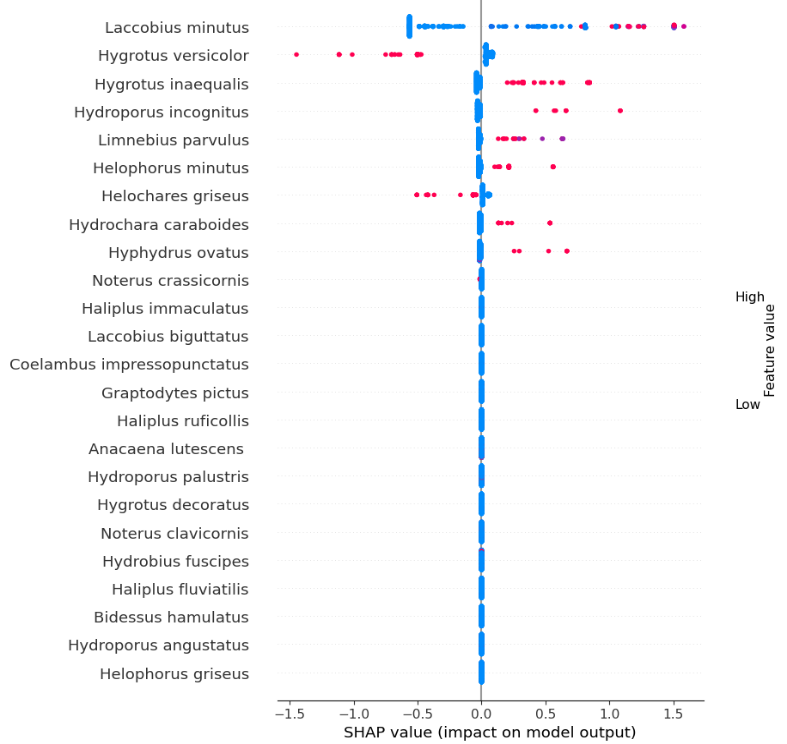

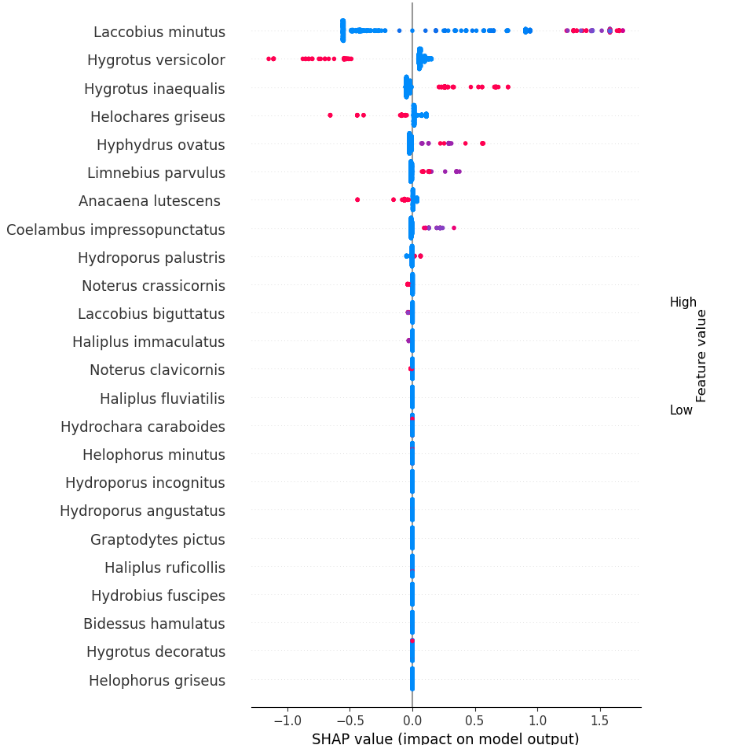

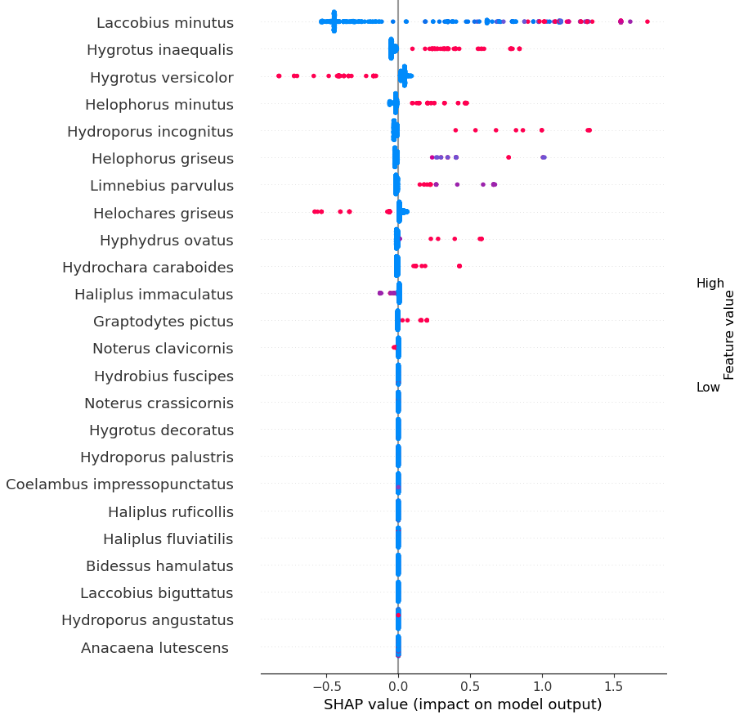

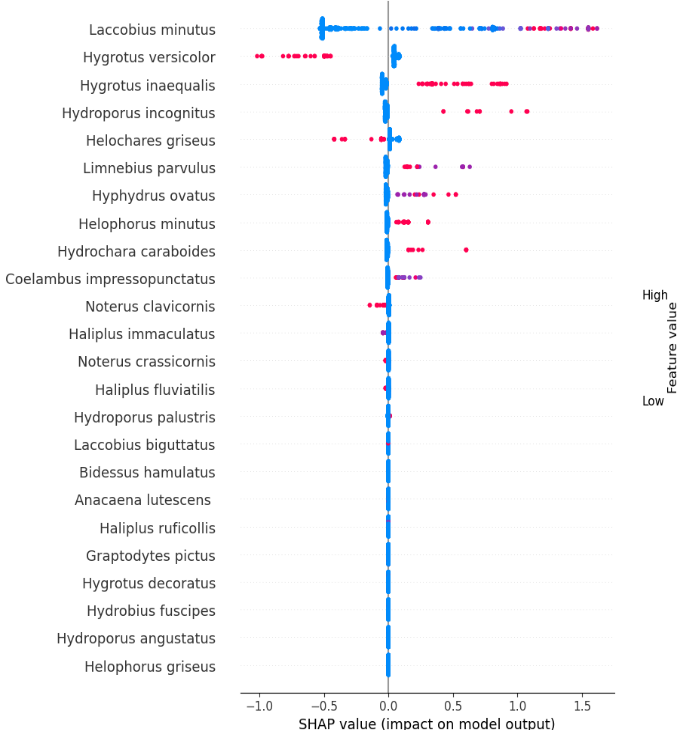


**5**

**4**

**3**

**2**

**1**


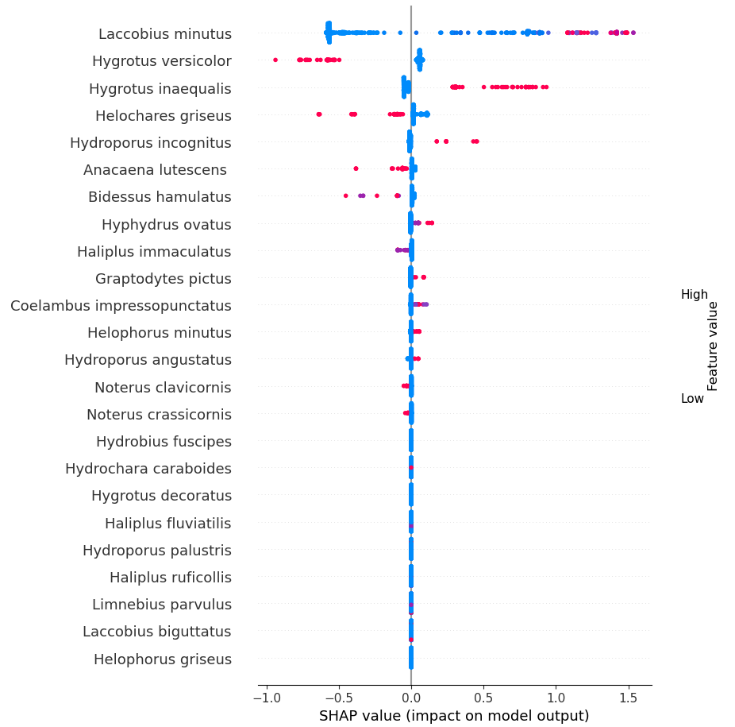


Fig. S2. Distribution of species (variables at y axis) migration between the mesotrophic lakes and gravel pits. Each of the five SHAP models (1 – 5) shows randomly selected individual SHAP modelling results. The values of the observations are shown: red dots indicates higher value of variable and blue dots lower value. Positive Shapley value indicate migration to pits from lakes and negative Shapley value indicate migration to lakes from pits.


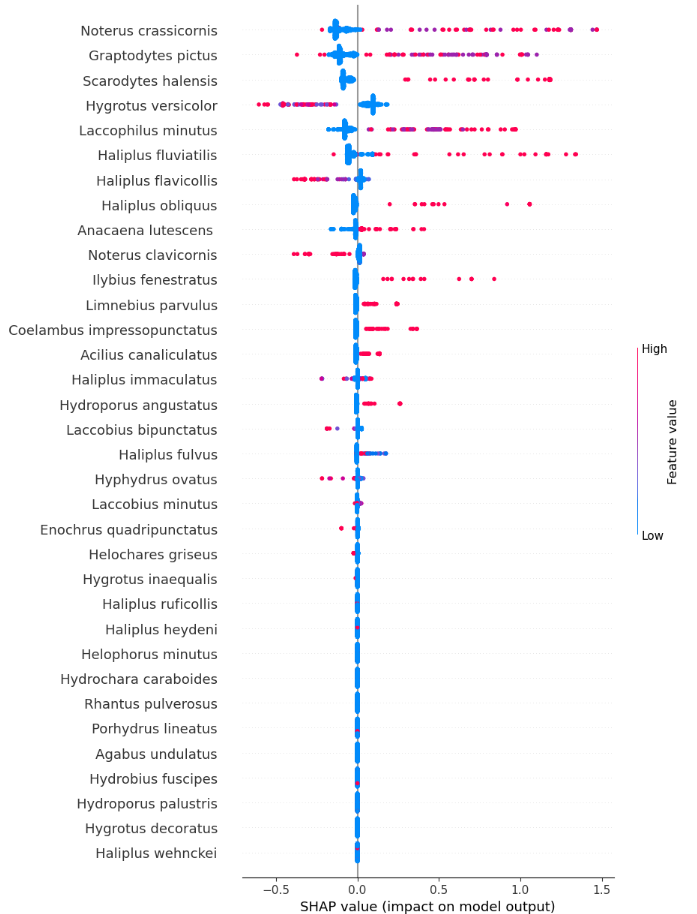

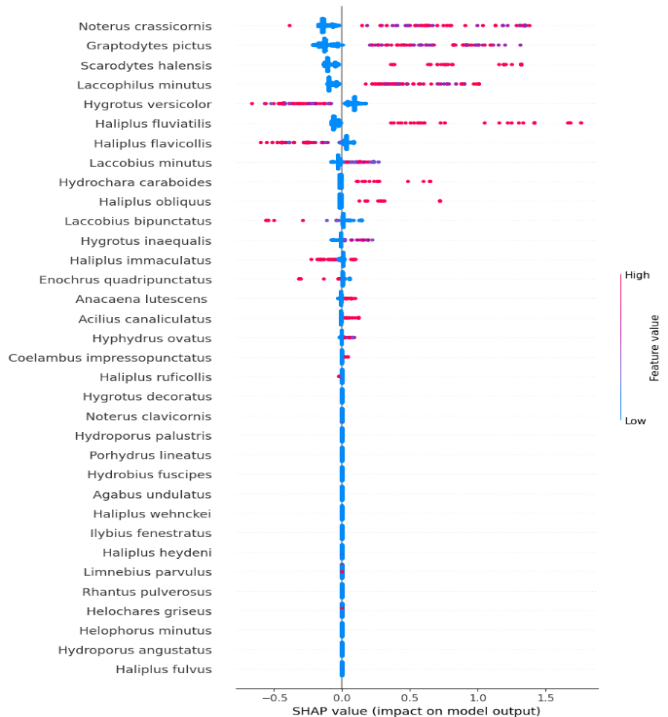


**2**

**1**


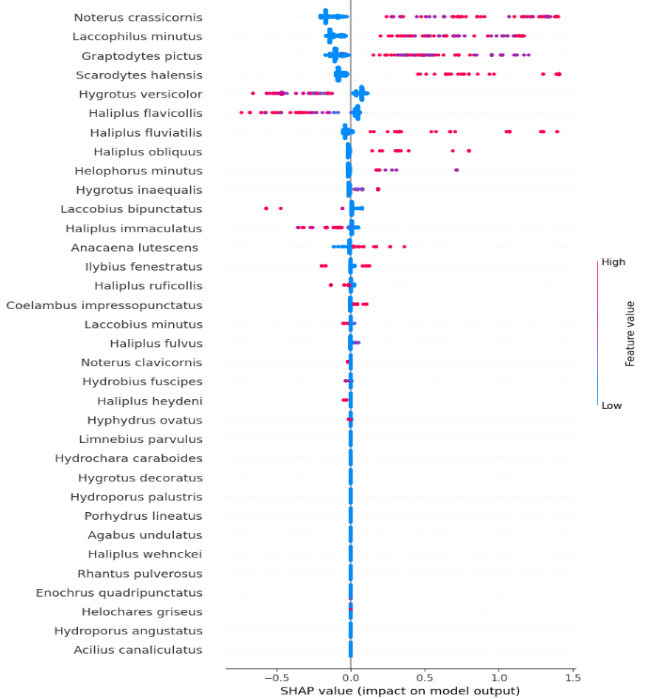

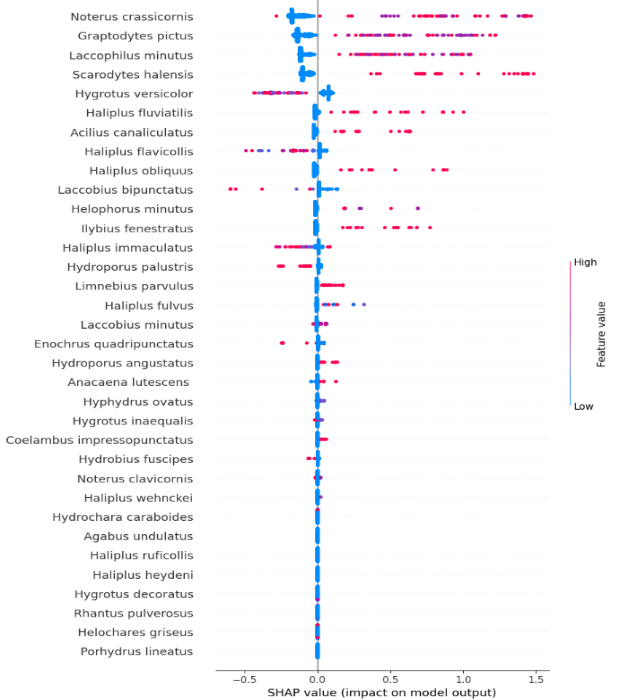


**4**

**3**


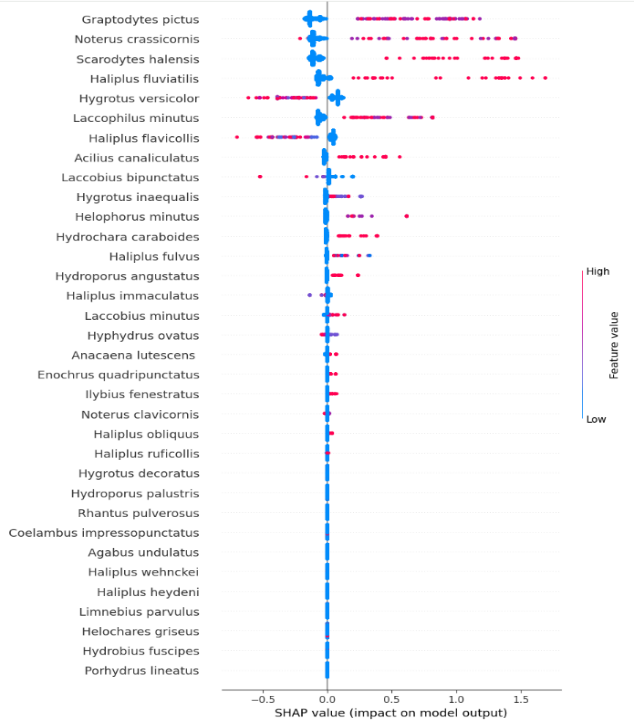


**5**

Fig. S3. Distribution of species (variables at y axis) migration between the eutrophic lakes and clay pits. Each of the five SHAP models (1 – 5) shows randomly selected individual SHAP modelling results. The values of the observations are shown: red dots indicates higher value of variable and blue dots lower value. Positive Shapley value indicate migration to pits from lakes and negative Shapley value indicate migration to lakes from pits.


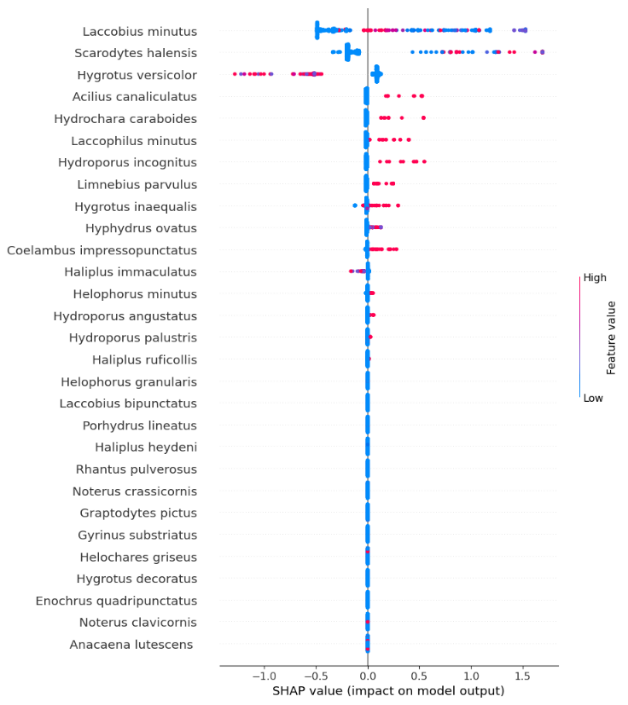

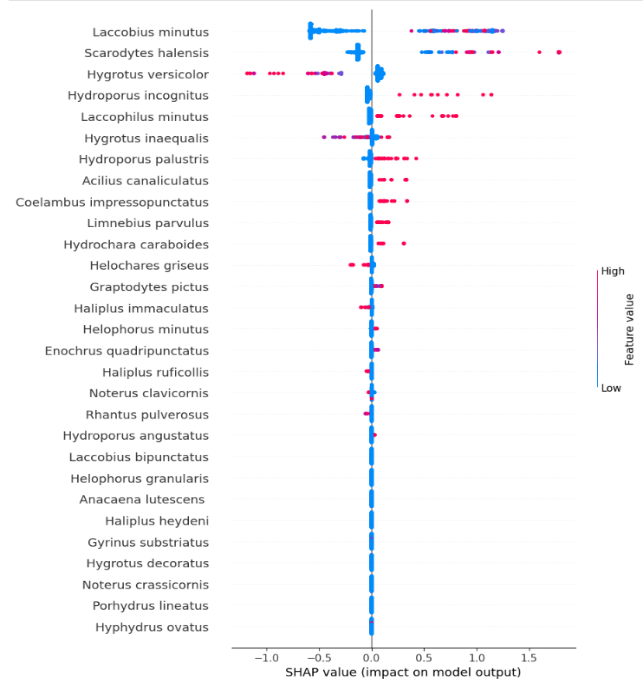

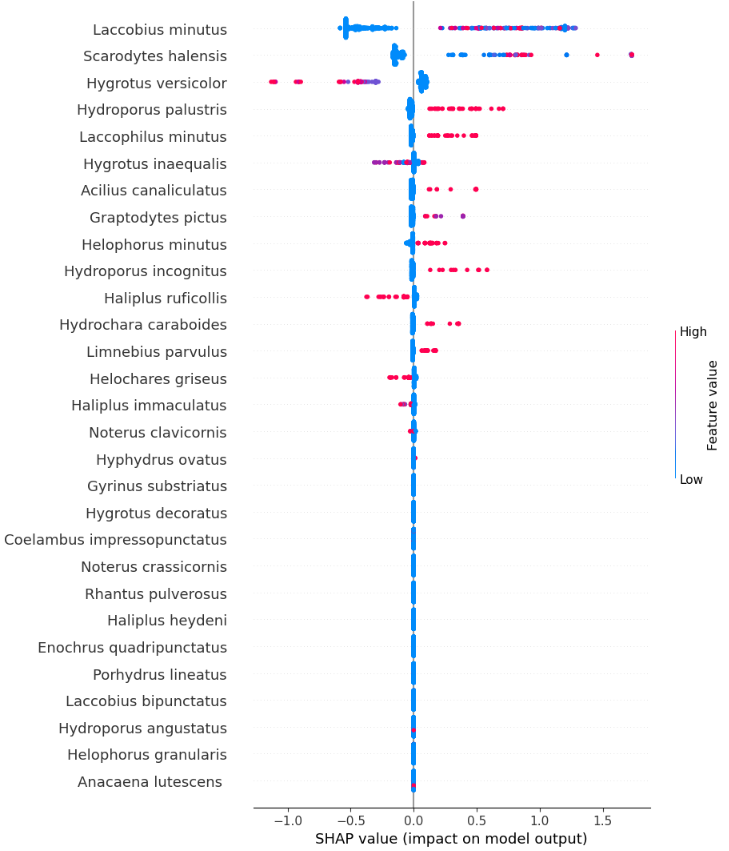

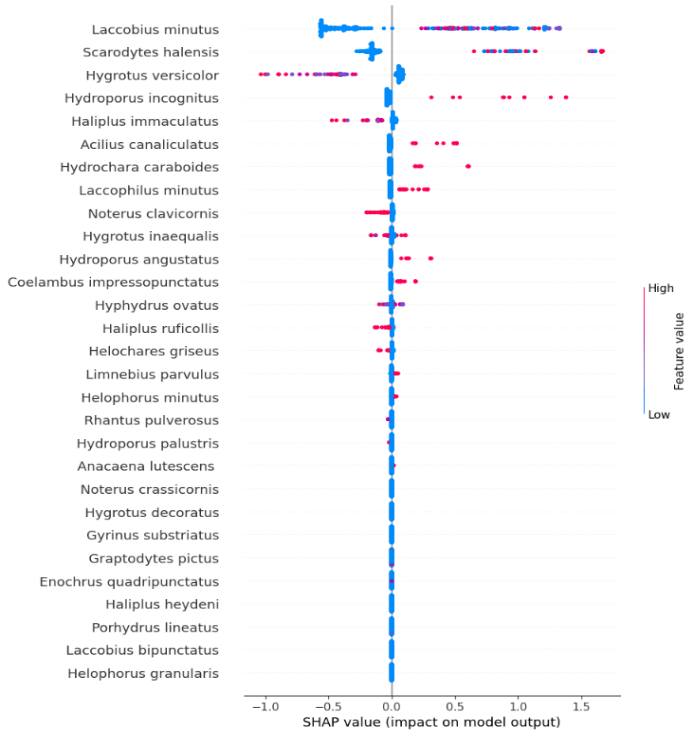

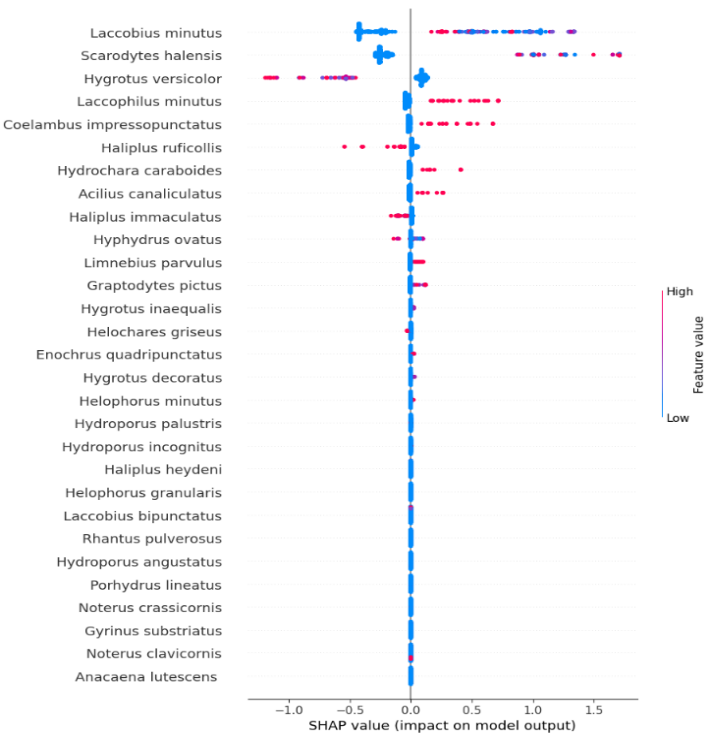


**2**

**1**

**4**

**3**

**5**

Fig. S4. Distribution of species (variables at y axis) migration between the eutrophic lakes and gravel pits. Each of the five SHAP models (1 – 5) shows randomly selected individual SHAP modelling results. The values of the observations are shown: red dots indicates higher value of variable and blue dots lower value. Positive Shapley value indicate migration to pits from lakes and negative Shapley value indicate migration to lakes from pits.


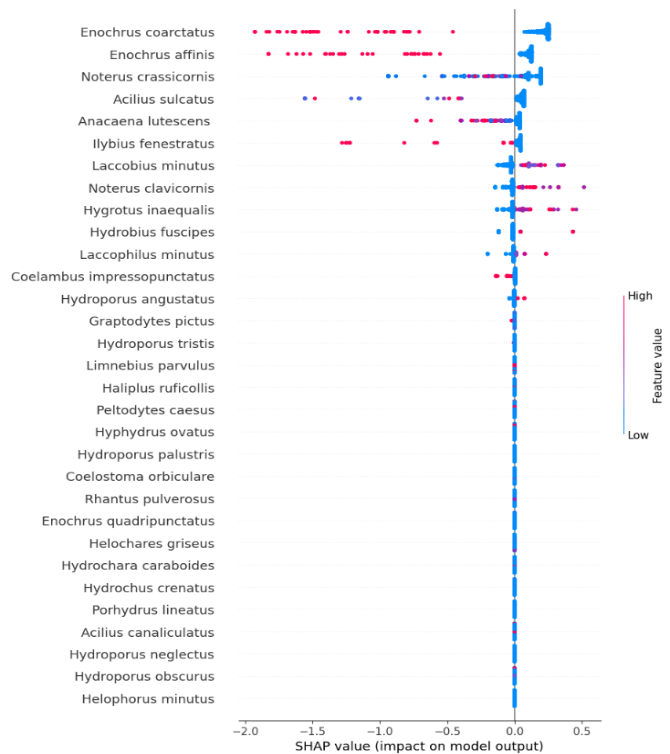

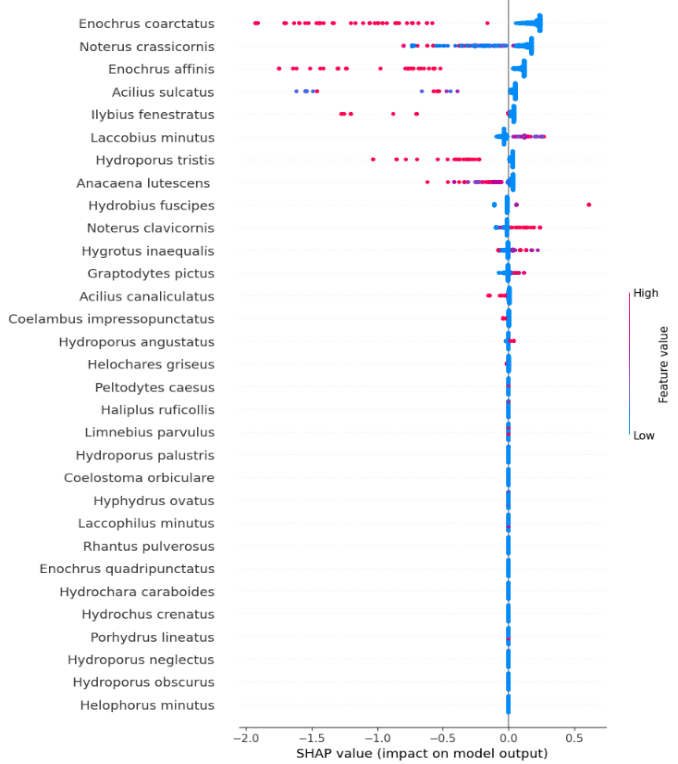


**2**

**1**


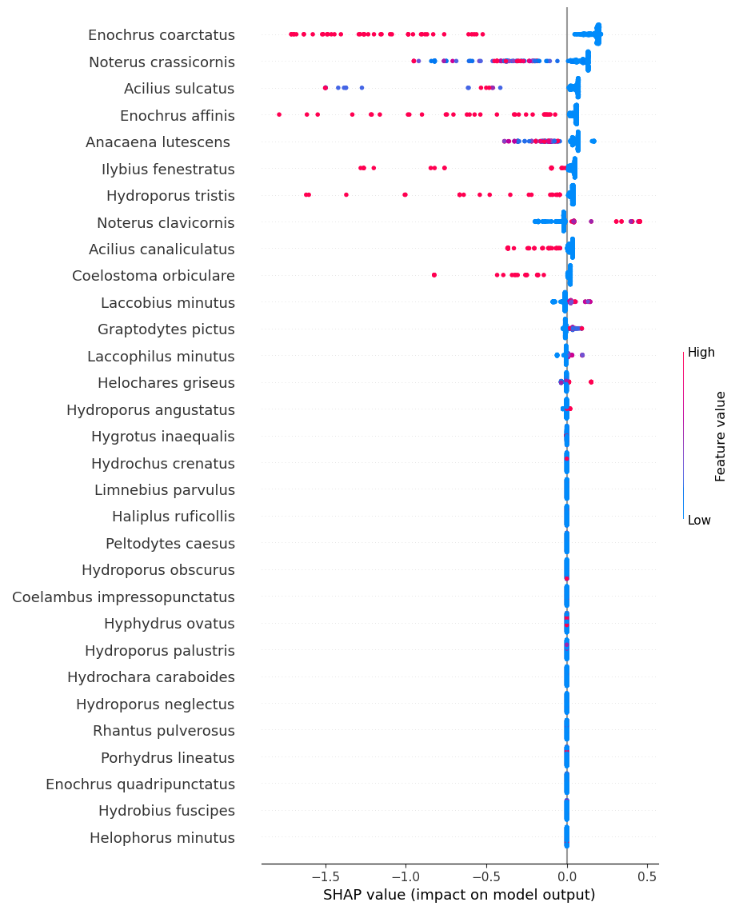

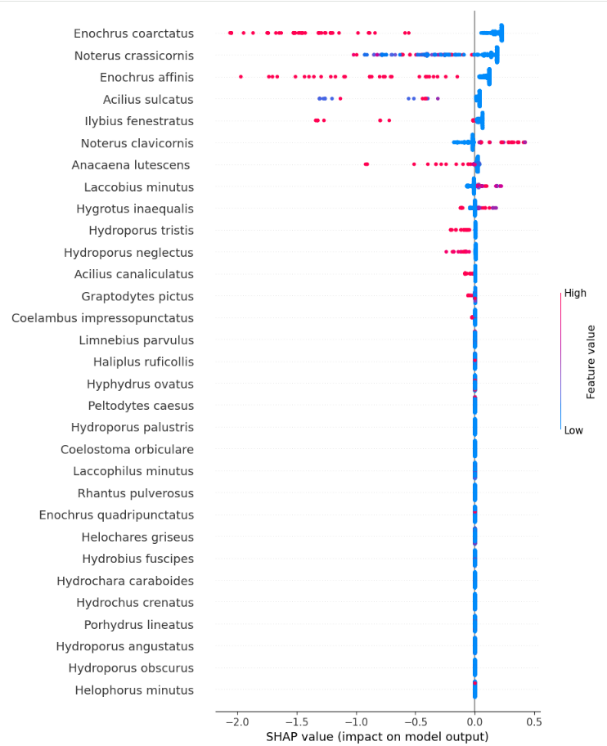


**4**

**3**


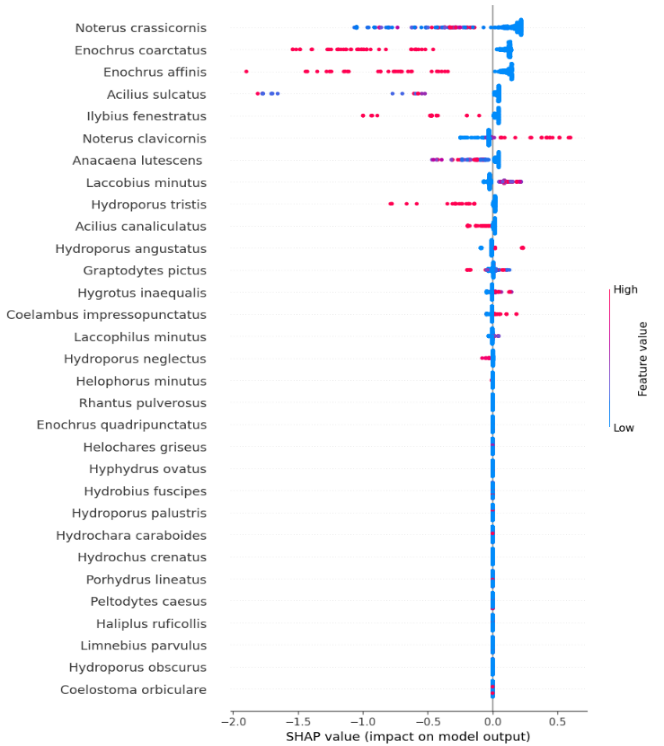


**5**

Fig. S5. Distribution of species (variables at y axis) migration between the dystrophic lakes and clay pits. Each of the five SHAP models (1 – 5) shows randomly selected individual SHAP modelling results. The values of the observations are shown: red dots indicates higher value of variable and blue dots lower value. Positive Shapley value indicate migration to pits from lakes and negative Shapley value indicate migration to lakes from pits.


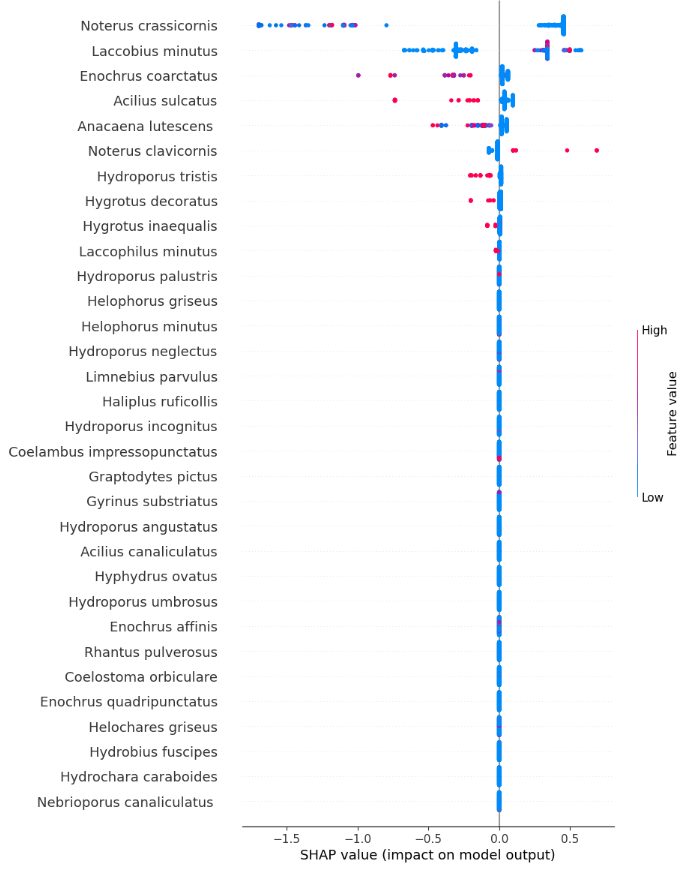

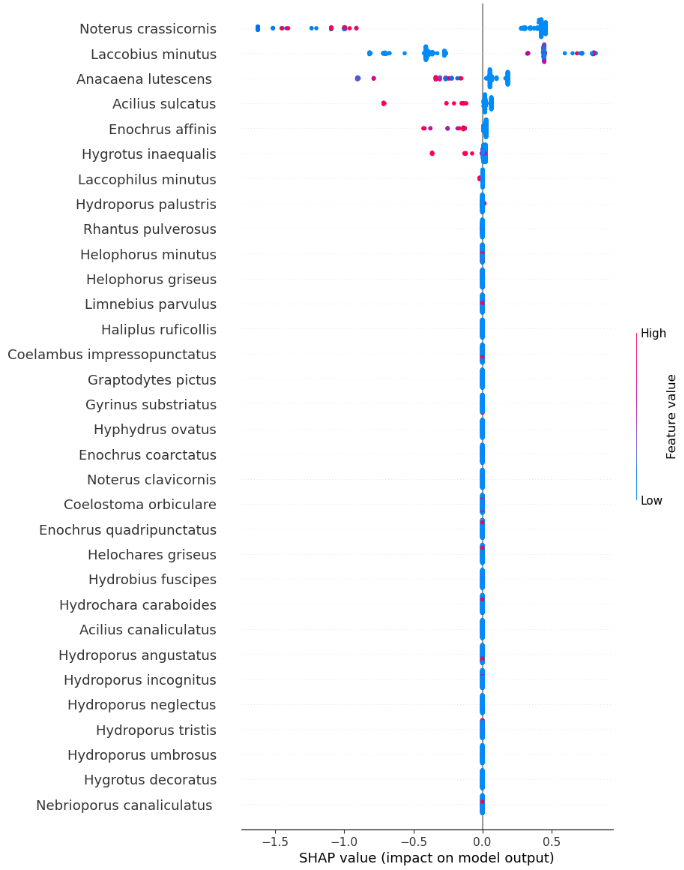

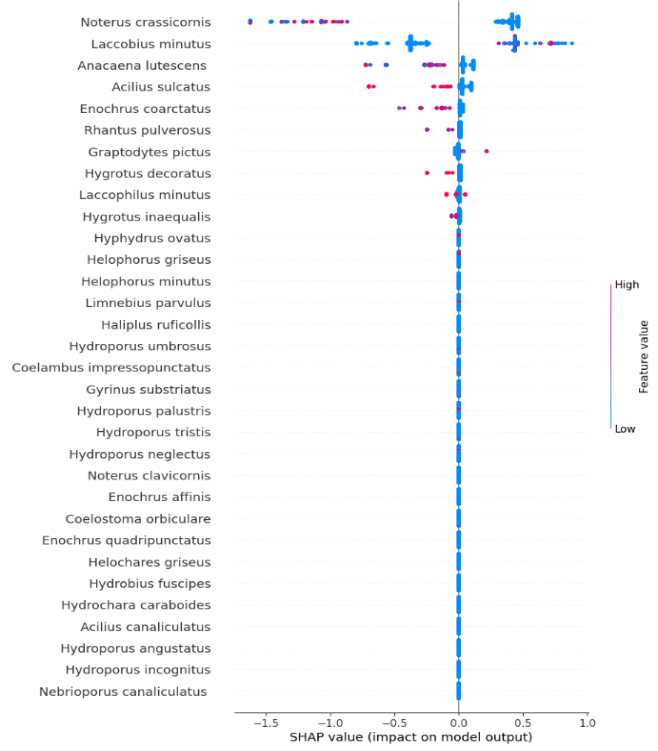

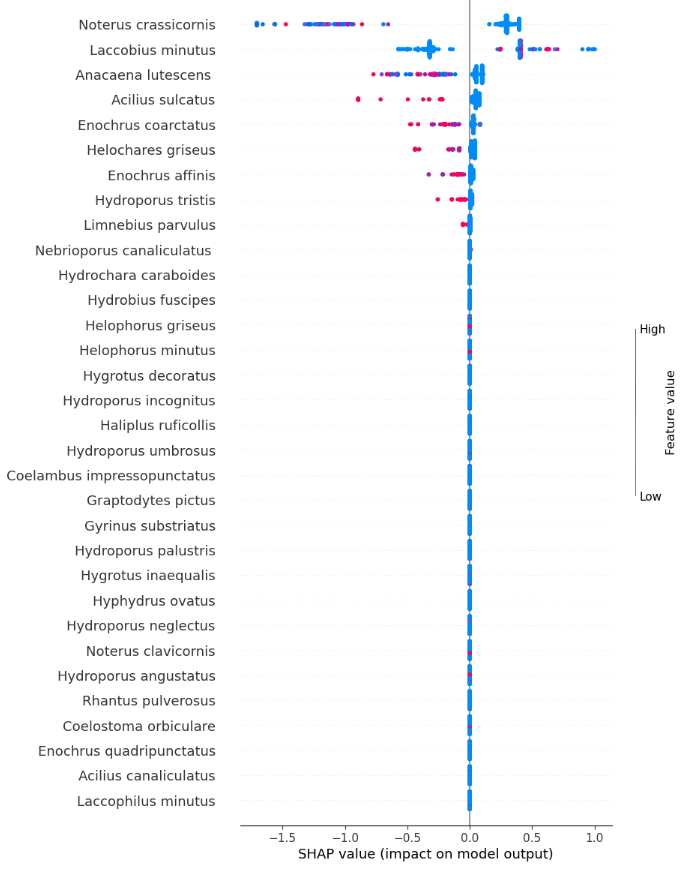


**2**

**1**

**4**

**3**


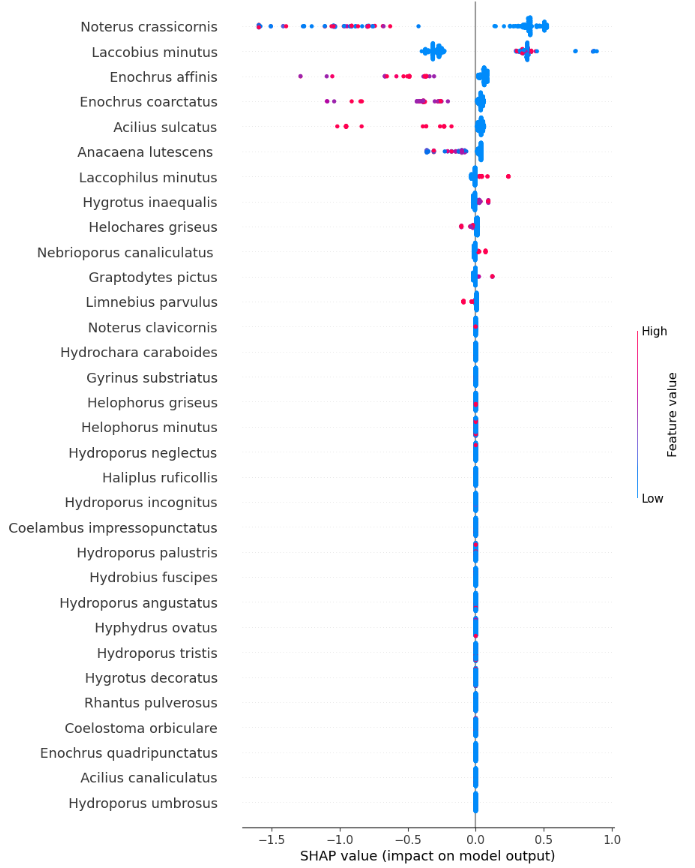


**5**

Fig. S6. Distribution of species (variables at y axis) migration between the dystrophic lakes and gravel pits. Each of the five SHAP models (1 – 5) shows randomly selected individual SHAP modelling results. The values of the observations are shown: red dots indicates higher value of variable and blue dots lower value. Positive Shapley value indicate migration to pits from lakes and negative Shapley value indicate migration to lakes from pits.

Fig. S1. Distribution of species (variables at y axis) migration between the mesotrophic lakes and clay pits. Each of the five SHAP models (1 – 5) shows randomly selected individual SHAP modelling results. The values of the observations are shown: red dots indicates higher value of variable and blue dots lower value. Positive Shapley value indicate migration to pits from lakes and negative Shapley value indicate migration to lakes from pits.


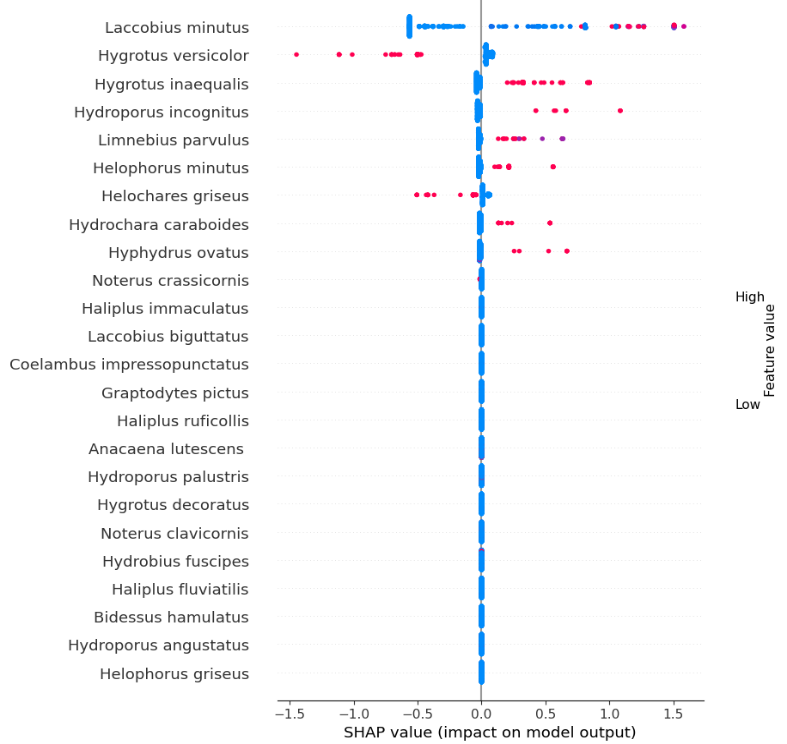

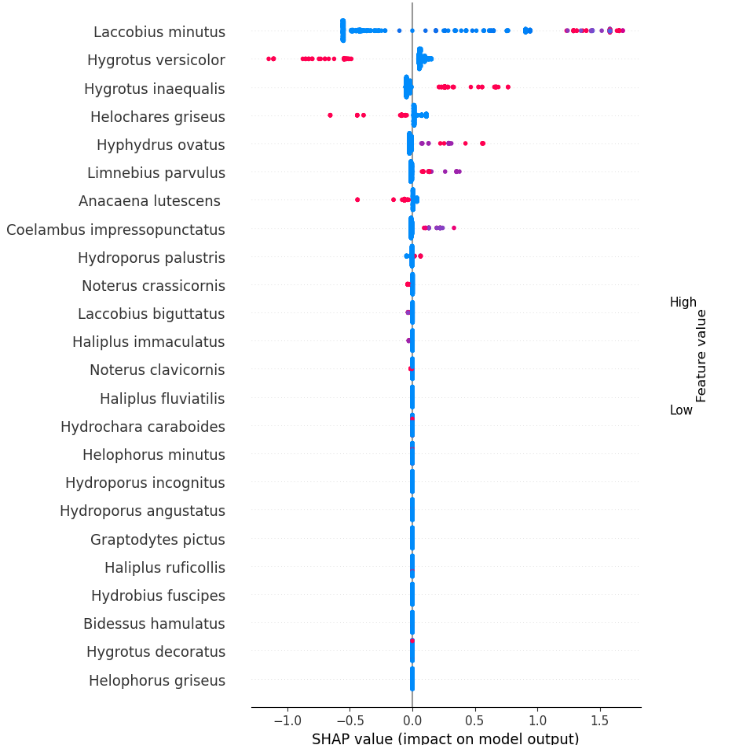

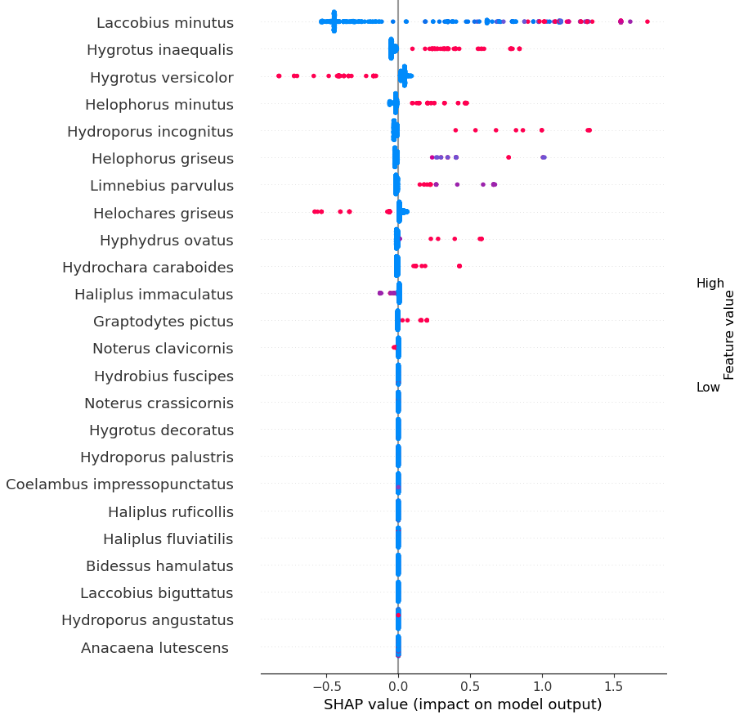

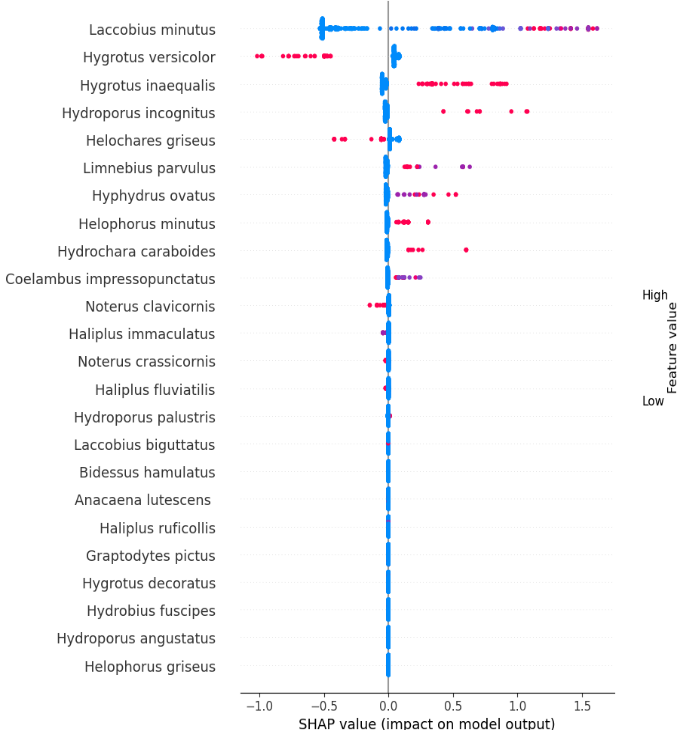


**5**

**4**

**3**

**2**

**1**


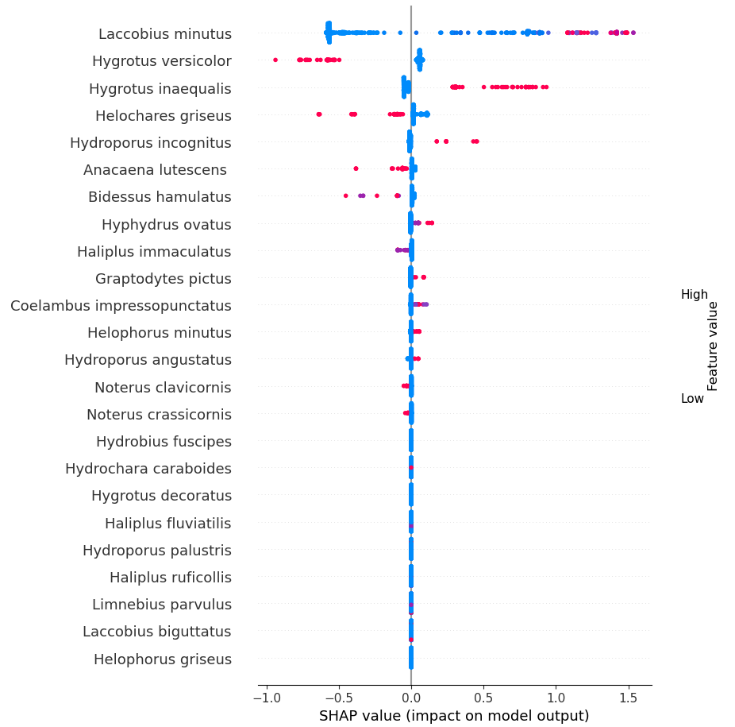


Fig. S2. Distribution of species (variables at y axis) migration between the mesotrophic lakes and gravel pits. Each of the five SHAP models (1 – 5) shows randomly selected individual SHAP modelling results. The values of the observations are shown: red dots indicates higher value of variable and blue dots lower value. Positive Shapley value indicate migration to pits from lakes and negative Shapley value indicate migration to lakes from pits.


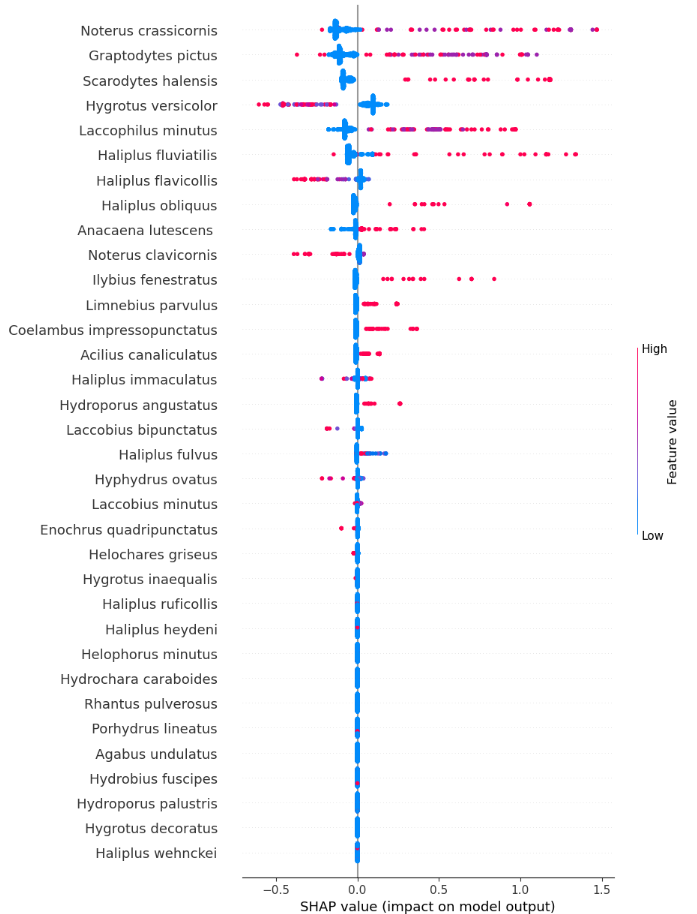

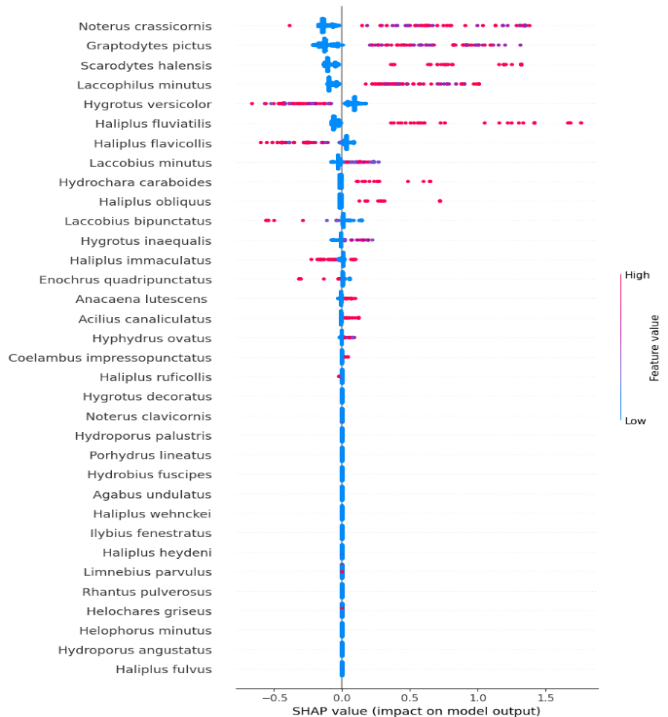


**2**

**1**


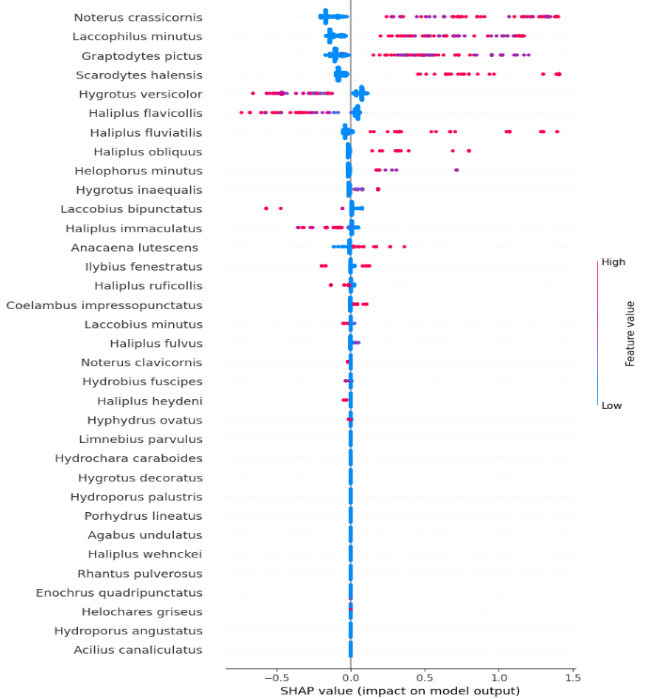

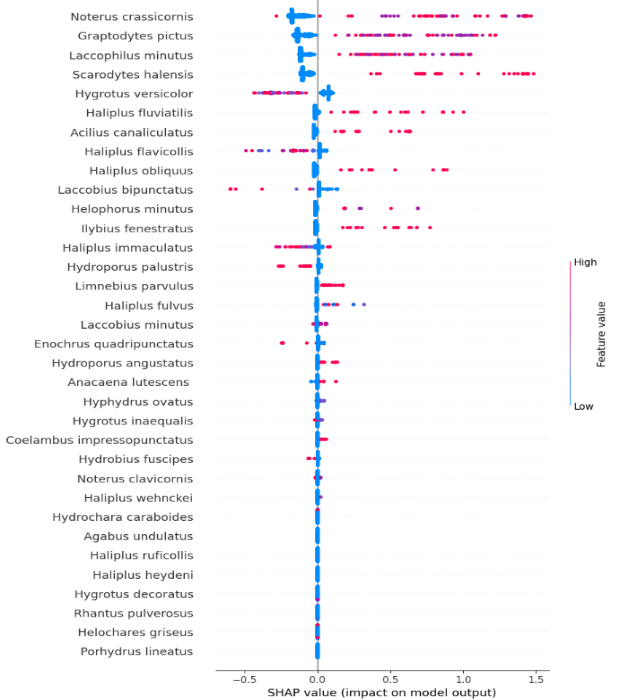


**4**

**3**


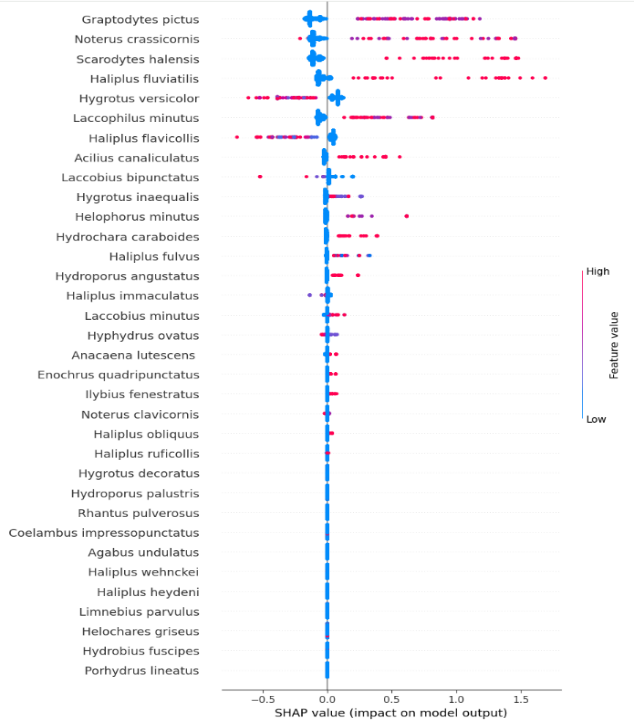


**5**

Fig. S3. Distribution of species (variables at y axis) migration between the eutrophic lakes and clay pits. Each of the five SHAP models (1 – 5) shows randomly selected individual SHAP modelling results. The values of the observations are shown: red dots indicates higher value of variable and blue dots lower value. Positive Shapley value indicate migration to pits from lakes and negative Shapley value indicate migration to lakes from pits.


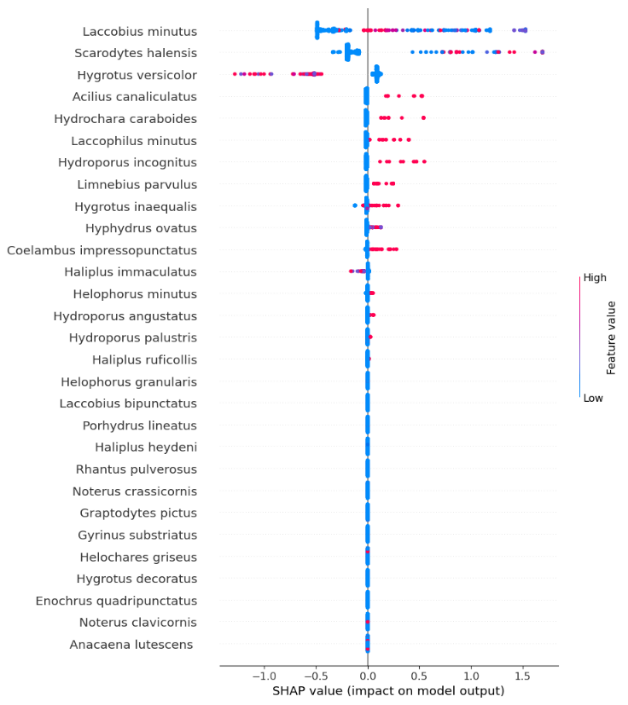

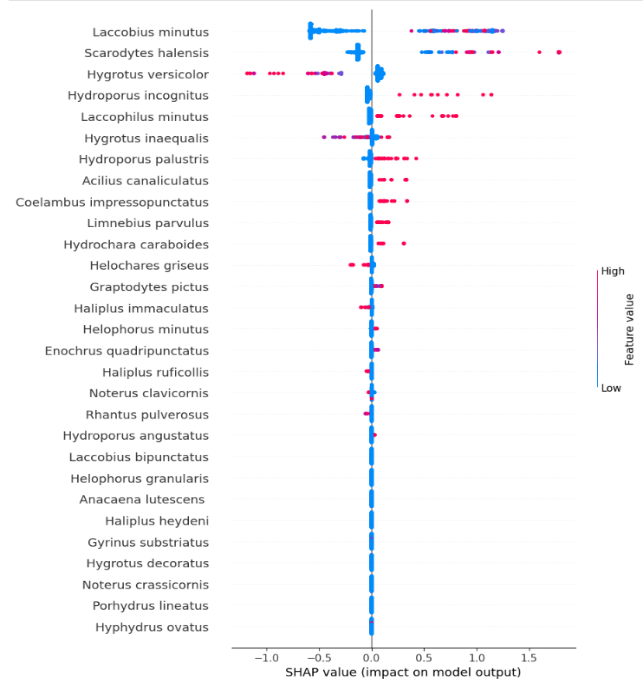

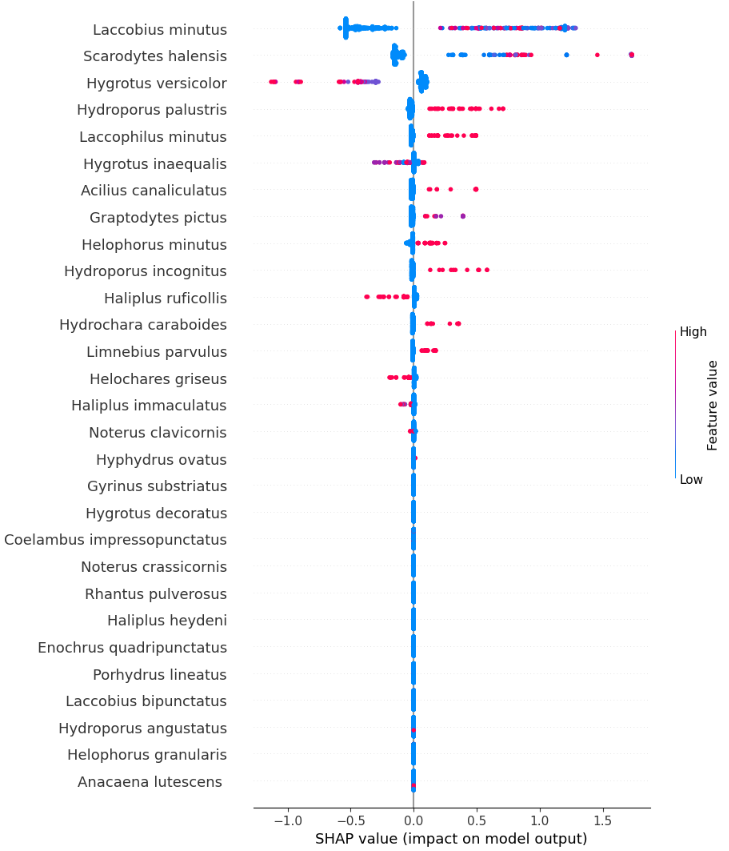

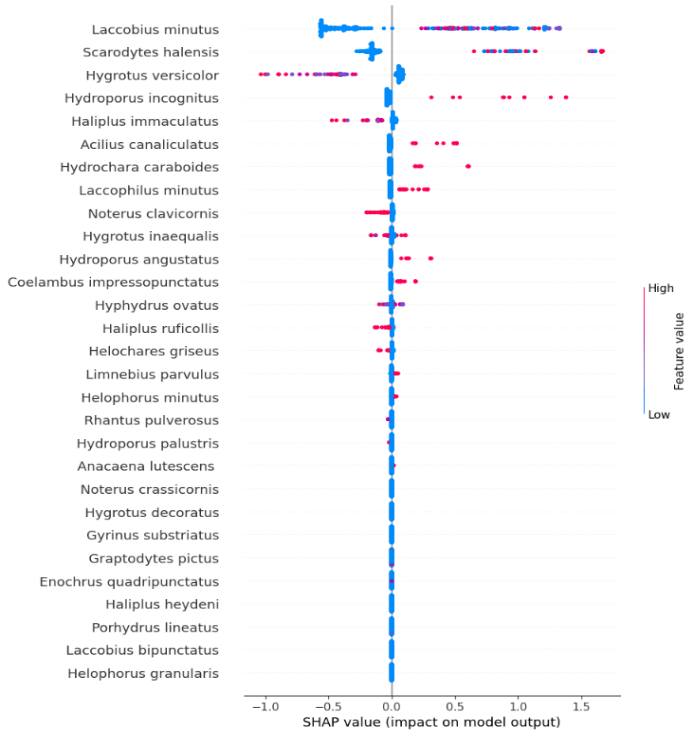

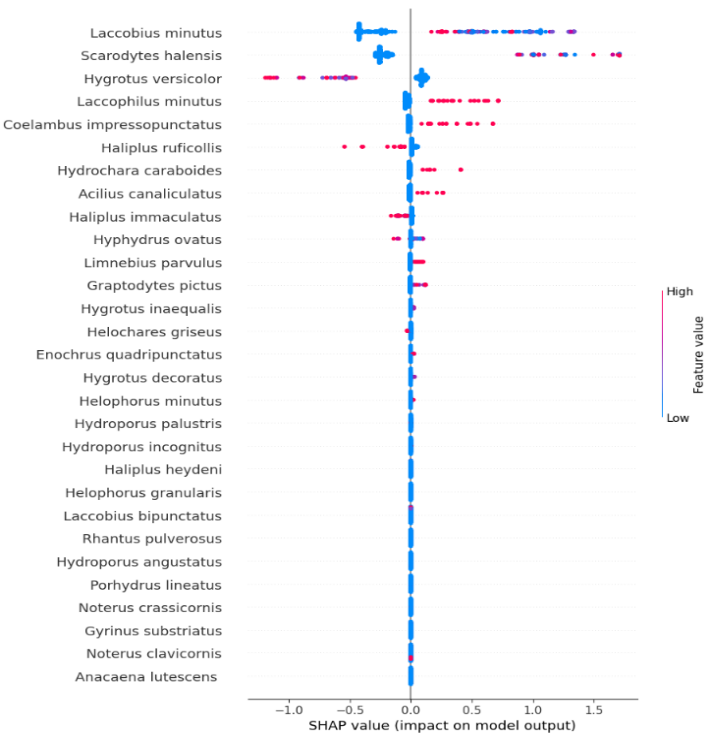


**2**

**1**

**4**

**3**

**5**

Fig. S4. Distribution of species (variables at y axis) migration between the eutrophic lakes and gravel pits. Each of the five SHAP models (1 – 5) shows randomly selected individual SHAP modelling results. The values of the observations are shown: red dots indicates higher value of variable and blue dots lower value. Positive Shapley value indicate migration to pits from lakes and negative Shapley value indicate migration to lakes from pits.


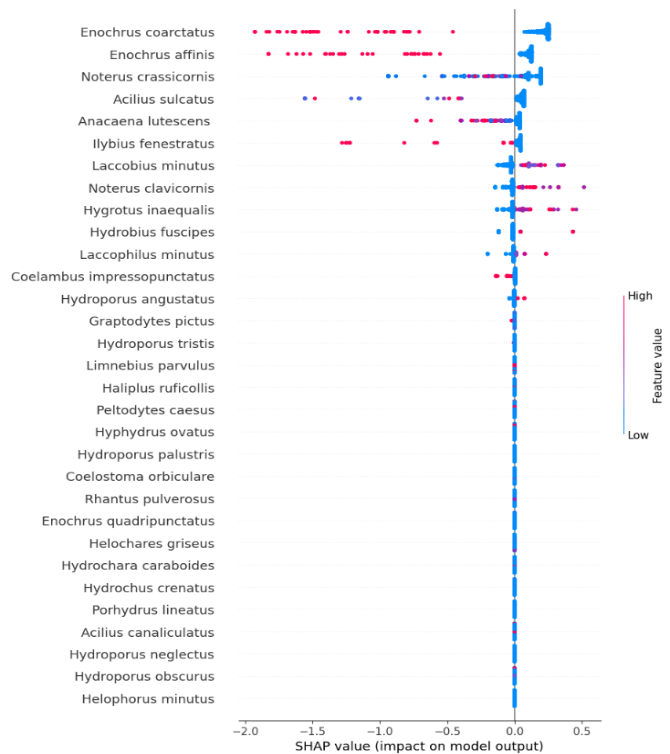

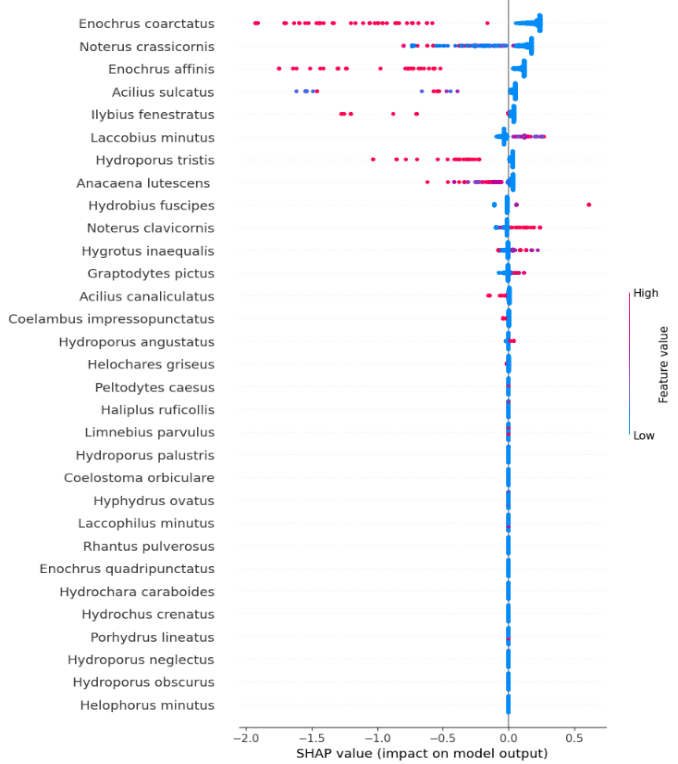


**2**

**1**


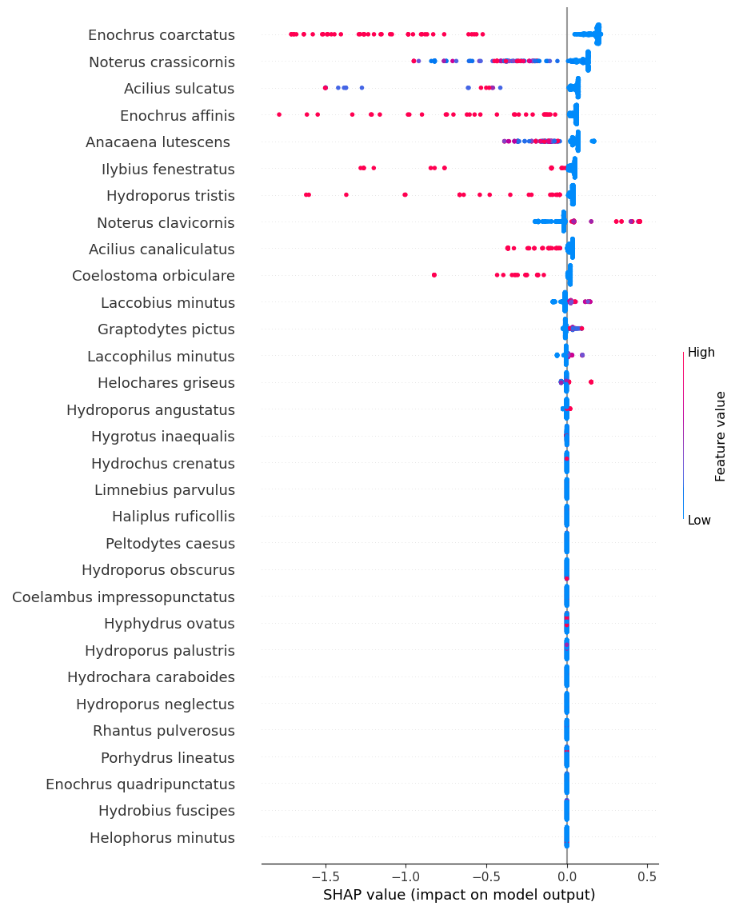

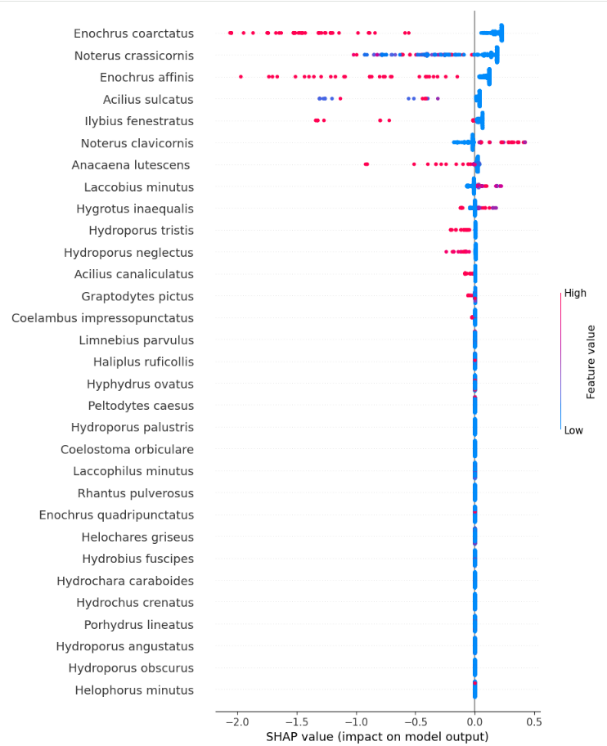


**4**

**3**


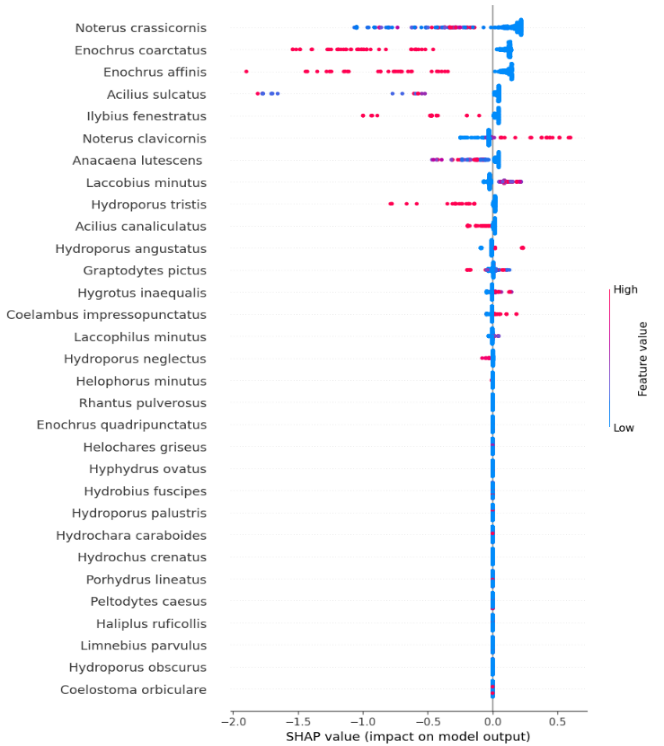


**5**

Fig. S5. Distribution of species (variables at y axis) migration between the dystrophic lakes and clay pits. Each of the five SHAP models (1 – 5) shows randomly selected individual SHAP modelling results. The values of the observations are shown: red dots indicates higher value of variable and blue dots lower value. Positive Shapley value indicate migration to pits from lakes and negative Shapley value indicate migration to lakes from pits.


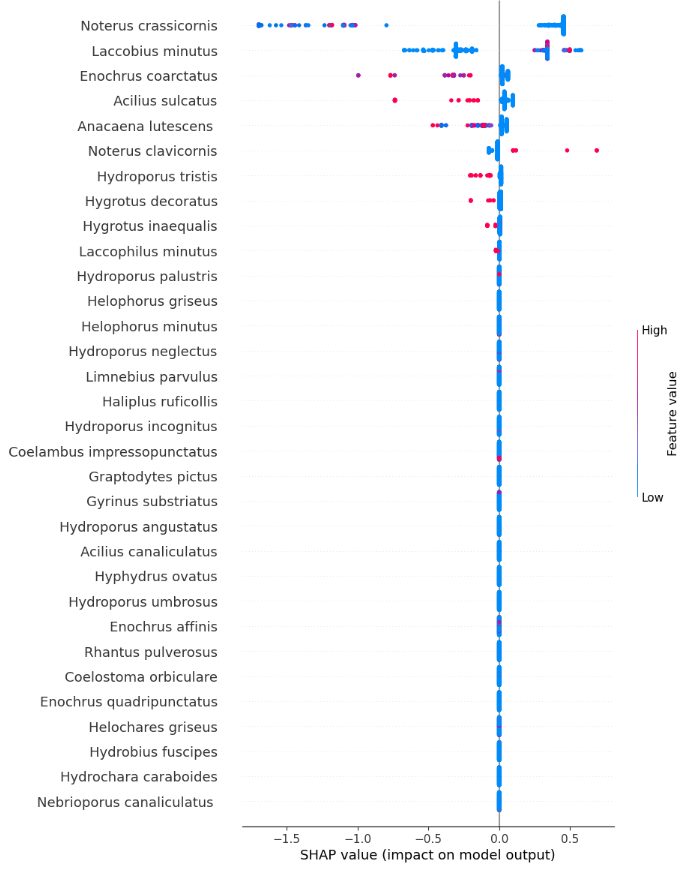

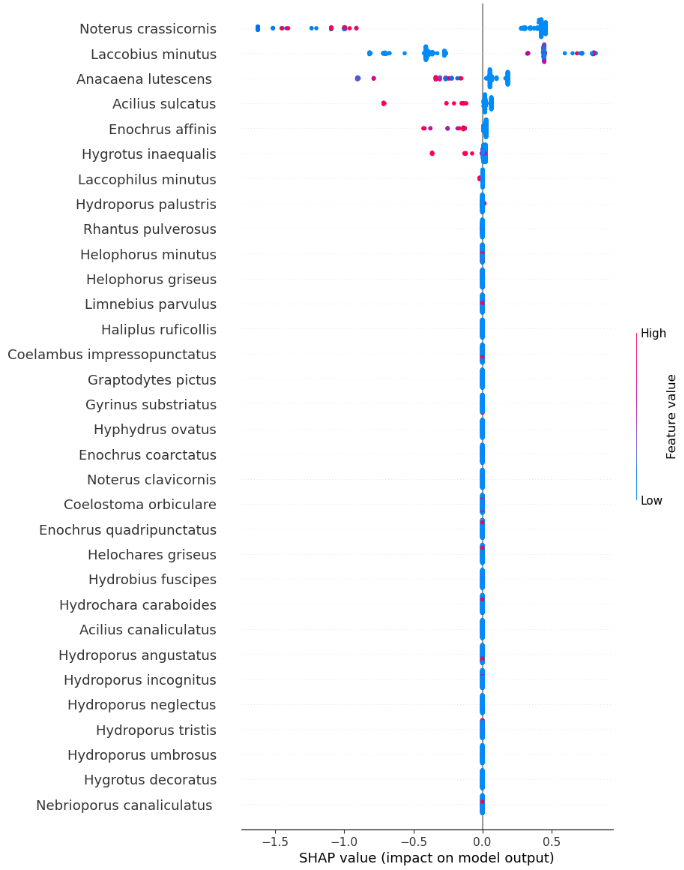

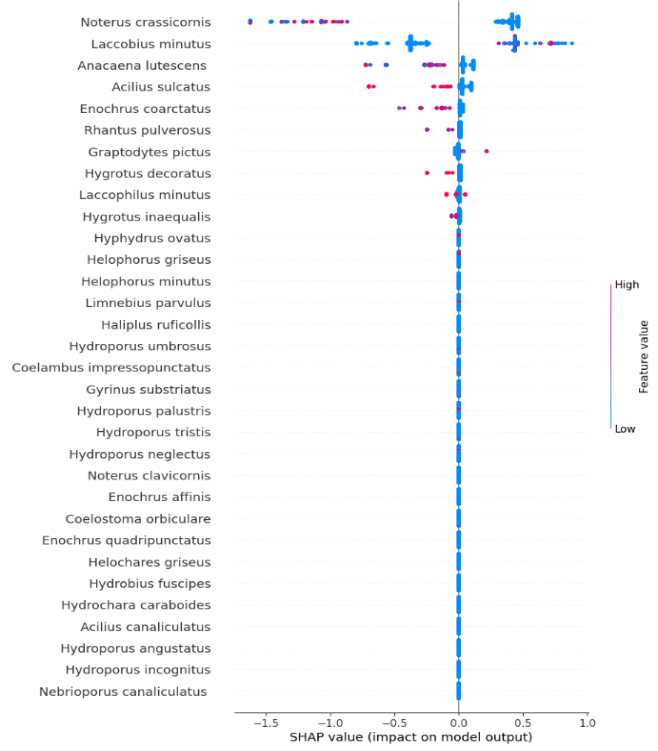

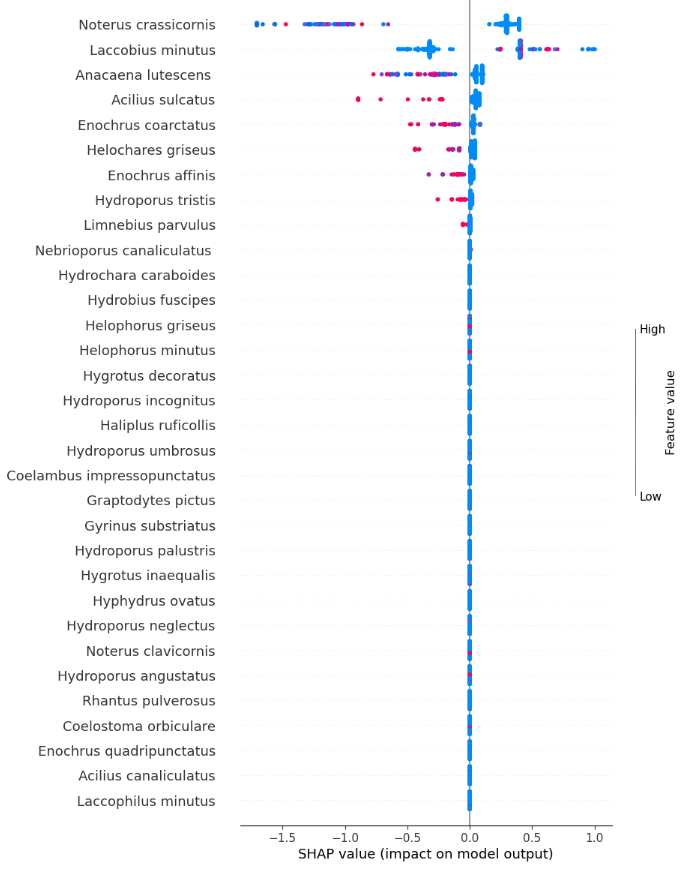


**2**

**1**

**4**

**3**


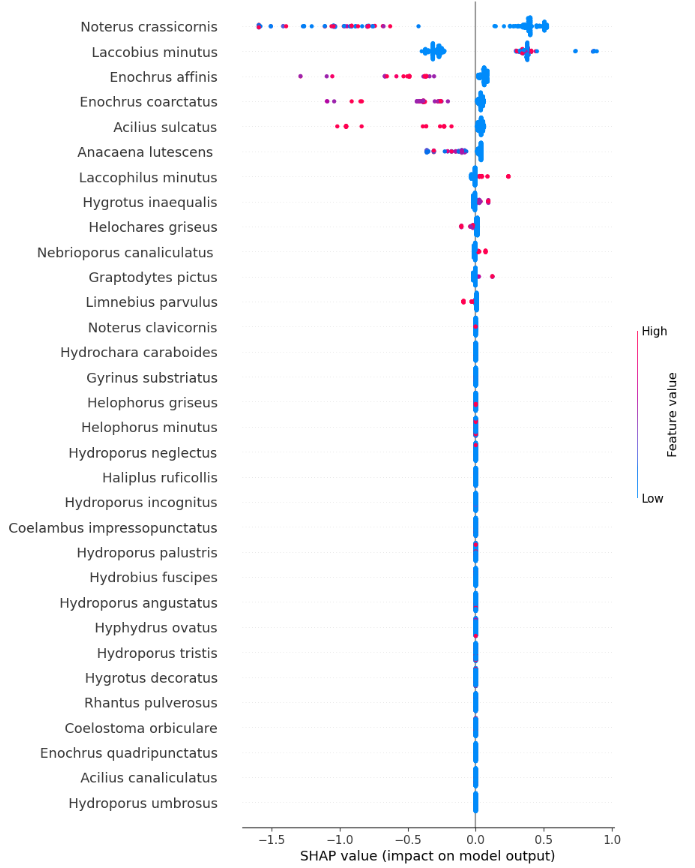


**5**

Fig. S6. Distribution of species (variables at y axis) migration between the dystrophic lakes and gravel pits. Each of the five SHAP models (1 – 5) shows randomly selected individual SHAP modelling results. The values of the observations are shown: red dots indicates higher value of variable and blue dots lower value. Positive Shapley value indicate migration to pits from lakes and negative Shapley value indicate migration to lakes from pits.
