# Supplementary material for: Water beetle networks differences and migration between natural lakes and post-exploitation water bodies
Source: Sci Rep. 2025 May 7;15:15898. doi: 10.1038/s41598-025-00525-1 (PMC12059192; doi:10.1038/s41598-025-00525-1)
Supplement: Supplementary file 2 — Supplementary Material 2 [file 41598_2025_525_MOESM2_ESM.docx]

Table S1. Quantitative occurrence of beetles in studied lake: Abbrev. – Abbreviations. Eg –ecological groups (L – lake and river species, T – tyrphophilous species, E – eurytopic species, A – psammophilous species, Rb – rheobionts). Type of water bodies: CP – clay pits, GP – gravel pits, ML – mesotrophic lakes, EL – eutrophic lakes, DL – dystrophic lakes, N – number of individuals, % – percentage share.

| **Species** | **Eg** | **Type of water bodies** | | | | | **Total** | |
| --- | --- | --- | --- | --- | --- | --- | --- | --- |
|  |  | **CP** | **GP** | **ML** | **EL** | **DL** | **N** | **%** |
| *Gyrinus aeratus* Steph. | L | – | – | 5 |  | 14 | 19 | 0.10 |
| *Gyrinus caspius* Mén. | E | – | 1 | – | – | – | 1 | 0.01 |
| *Gyrinus distinctus* Aubé | L | – |  | 58 | 28 | – | 86 | 0.43 |
| *Gyrinus marinus* Gyll. | L | 15 | 23 | 5 | – | – | 43 | 0.22 |
| *Gyrinus minutus* (Fabr.) | E | – | 4 | – | – | – | 4 | 0.02 |
| *Gyrinus natator* (L.) | E | – | 2 | – | 5 | – | 7 | 0.04 |
| *Gyrinus paykuli* Ochs. | E | – |  | 5 | 6 | 1 | 12 | 0.06 |
| *Gyrinus substriatus* Steph. | E | – | 37 |  | 7 | 4 | 48 | 0.24 |
| *Gyrinus suffriani* Scriba | E | – | 1 | 1 | – | 3 | 5 | 0.03 |
| *Orectochilus villosus* (O.F.Müll) | Rb | – | – | 2 | – | – | 2 | 0.01 |
| *Haliplus confinis* Steph. | A | 3 | – | 27 | 62 | – | 92 | 0.46 |
| *Haliplus flavicollis* Sturm | L | 34 | – | 117 | 1434 | – | 1585 | 7.96 |
| *Haliplus fluviatilis* Aubé | L | 40 | 6 | 36 | 181 | – | 263 | 1.32 |
| *Haliplus fulvicollis* Er. | T | 2 | 3 | 2 | – | 3 | 10 | 0.05 |
| *Haliplus fulvus* (Fabr.) | L | 87 | 3 | 3 | 33 |  | 126 | 0.63 |
| *Haliplus heydeni* Wehncke | E | 8 | 3 |  | 68 | – | 79 | 0.40 |
| *Haliplus immaculatus* Gerh*.* | E | 43 | 2 | 29 | 1530 | – | 1604 | 8.05 |
| *Haliplus lineatocollis* (Marsh.) | Rb | – | – | – | 3 | – | 3 | 0.02 |
| *Haliplus lineolatus* Mann. | E | 37 | 1 | 3 | – | – | 41 | 0.21 |
| *Haliplus obliquus* (Fabr.) | A | 20 | 5 | – | 2 | – | 27 | 0.14 |
| *Haliplus ruficollis* (De Geer) | E | 43 | 20 | 19 | 199 | 3 | 284 | 1.43 |
| *Haliplus wehnckei* Gerh. | E | 29 | – | – | 20 | – | 49 | 0.25 |
| *Peltodytes caesus* (Duft.) | E | 41 | 64 | 1 | – | 1 | 107 | 0.54 |
| *Noterus clavicornis* (De Geer) | E | 193 | 17 | 30 | 144 | 3 | 387 | 1.94 |
| *Noterus crassicornis* (O.F.Müll) | E | 565 | 10 | 207 | 1855 | 613 | 3250 | 16.31 |
| *Agabus bipustulatus* (L.) | E | – | 1 | – | 3 | – | 4 | 0.02 |
| *Agabus congener* (Thunb.) | E | – | – | – | 2 | 1 | 3 | 0.02 |
| *Agabus fuscipennis* (Payk.) | E | – | – | 1 | 5 | – | 6 | 0.03 |
| *Agabus guttatus* (Payk.) | Rb | – | – | – | 2 | – | 2 | 0.01 |
| *Agabus neglectus* Er. | T | – | – | – | 4 | – | 4 | 0.02 |
| *Agabus paludosus* (Fabr.) | L | – | 2 | – |  | – | 2 | 0.01 |
| *Agabus sturmii* (Gyll.) | E | – | 1 | – | 50 | – | 51 | 0.26 |
| *Agabus undulatus* (Schrank) | E | 13 | 1 | 1 | 14 | 1 | 30 | 0.15 |
| *Agabus unguicularis* (Thoms.) | T | – | – | – | 4 | – | 4 | 0.02 |
| Platambus maculatus (L.) | L | – | – | 32 | 40 | – | 72 | 0.36 |
| *Ilybius ater* (De Geer) | T | 5 | – | 1 | 5 | 2 | 13 | 0.07 |
| *Ilybius crassus* (Thoms.) | T | 3 | – | – | 1 | – | 4 | 0.02 |
| *Ilybius fenestratus* (Fabr.) | L | 18 |  | 109 | 108 | 24 | 259 | 1.30 |
| *Ilybius fuliginosus* (Fabr.) | L | – | 9 | – | 5 | 1 | 15 | 0.08 |
| *Ilybius guttiger* (Gyll.) | T | 1 | – | – | – | – | 1 | 0.01 |
| *Ilybius obscurus* (Marsh.) | T | – | 1 | 1 | 2 | 2 | 6 | 0.03 |
| *Ilybius quadriguttatus* (Lacord.) | T | – | – | – | 2 | – | 2 | 0.01 |
| *Ilybius similis* (Thoms.) | T | – | 2 | 1 | – | – | 3 | 0.02 |
| *Ilybius subaeneus* Er. | T | – | – | – | – | 2 | 2 | 0.01 |
| *Colymbetes fuscus* (L.) | E | 3 | – | – | 12 | 2 | 17 | 0.09 |
| *Colymbetes paykuli* Er. | T | 1 | 1 | – | 1 | – | 3 | 0.02 |
| *Colymbetes striatus* (L.) | T | 1 | 1 | – | 8 | 0 | 10 | 0.05 |
| *Rhantus bistriatus* (Bergstr.) | E | 1 | – | – | – | – | 1 | 0.01 |
| *Rhantus exsoletus* (Forst) | E | 2 | – | 2 | 7 | – | 11 | 0.06 |
| *Rhantus grapii* (Gyll.) | E | 1 | – | – | 3 | 2 | 6 | 0.03 |
| *Rhantus incognitus* Scholz | E | – | – | – | 1 | – | 1 | 0.01 |
| *Rhantus latitans* Sharp | E | 1 | – | – | 2 | – | 3 | 0.02 |
| *Rhantus notaticollis* Aubé | E | 3 | 2 | – |  | – | 5 | 0.03 |
| *Rhantus notatus* (Fabr.) | E | 5 | 11 | – | 2 | 1 | 19 | 0.10 |
| *Rhantus suturalis* (Macleay) | E | 7 | 39 | – | 9 | 2 | 57 | 0.29 |
| *Liopterus ruficollis* (Fabr.) | T | 3 | 1 | 2 | – | – | 6 | 0.03 |
| *Acilius canaliculatus* (Nic.) | T | 26 | 10 | – | 15 | 26 | 77 | 0.39 |
| *Acilius sulcatus* (L.) | E | 2 | 6 | – | 2 | 58 | 68 | 0.34 |
| *Graphoderus austriacus* (Sturm) | E | 1 | 1 | – | – | – | 2 | 0.01 |
| *Graphoderus bilineatus* (De Geer) | E | – | – | – | – | 1 | 1 | 0.01 |
| *Graphoderus cinereus* (L.) | E | 3 | 1 | – | 2 | 5 | 11 | 0.06 |
| *Graphoderus zonatus* Hoppe | E | – | – | – |  | 5 | 5 | 0.03 |
| *Cybister lateralimarginalis* (De Geer) | E | – | – | 4 | 1 | – | 5 | 0.03 |
| *Dytiscus circumcinctus* Ahr. | E | – | 2 | – | 1 | – | 3 | 0.02 |
| *Dytiscus dimidiatus* Bergstr. | E | 3 | 1 | 2 | 1 | – | 7 | 0.04 |
| *Dytiscus marginalis* (L.) | E | 2 | 4 | – | 1 | 5 | 12 | 0.06 |
| *Hydaticus aruspex* Clark | T | 6 | 1 | – |  | 2 | 9 | 0.05 |
| *Hydaticus seminiger* (De Geer) | E | 6 | 5 | 1 | 15 | 2 | 29 | 0.15 |
| *Hydaticus transversalis* (Pontop.) | E | 8 | – | 1 | 2 | – | 11 | 0.06 |
| *Nebrioporus canaliculatus* (Lacord.) | A | 1 | 49 | – | – | 1 | 51 | 0.26 |
| *Nebrioporus depressus* (Fabr.) | Rb | – | – | 1 | 3 | – | 4 | 0.02 |
| *Graptodytes pictus* (Fabr.) | E | 212 | 14 | 7 | 26 | 24 | 283 | 1.42 |
| *Hydroporus angustatus* Sturm | T | 16 | 11 | 11 | 18 | 12 | 68 | 0.34 |
| *Hydroporus dorsalis* (Fabr.) | E | 1 | 2 | – | 3 | – | 6 | 0.03 |
| *Hydroporus erythrocephalus* (L.) | T | 3 | 7 | – | 4 | 4 | 18 | 0.09 |
| *Hydroporus fuscipennis* Schaum | E | – | 10 | – | – | – | 10 | 0.05 |
| *Hydroporus incognitus* Sharp | T | 16 | 22 | 1 | 2 | 6 | 47 | 0.24 |
| *Hydroporus melanocephalus* (Marsh.) | T | – | – | – | 1 | – | 1 | 0.01 |
| *Hydroporus memnonius* Nic. | T | – | – | – | – | 4 | 4 | 0.02 |
| *Hydroporus neglectus* Shaum | T | 4 | 5 | – | 1 | 25 | 35 | 0.18 |
| *Hydroporus nigrita* (Fabr.) | T | – | 1 | – | – | – | 1 | 0.01 |
| *Hydroporus notatus* Sturm | T | – | – | – | – | 1 | 1 | 0.01 |
| *Hydroporus obscurus* Sturm | T | 1 | – | – | – | 53 | 54 | 0.27 |
| *Hydroporus palustris* (L.) | E | 20 | 39 | 16 | 87 | 10 | 172 | 0.86 |
| *Hydroporus planus* (Fabr.) | E | 3 | 50 | – | 5 | – | 58 | 0.29 |
| *Hydroporus pubescens* Gyll.) | T | – | 1 | – | – | – | 1 | 0.01 |
| *Hydroporus scalesianus* Steph. | T | – | – | – | – | 8 | 8 | 0.04 |
| *Hydroporus striola* (Gyll.) | E | – | 8 | – | 4 | – | 12 | 0.06 |
| *Hydroporus tristis* (Payk.) | T | 4 | 9 | 6 | – | 100 | 119 | 0.60 |
| *Hydroporus umbrosus* (Gyll.) | T | 2 | 5 | 1 | – | 19 | 27 | 0.14 |
| *Hydrovatus cuspidatus* (Kunze) | E | – | – | 2 | – | – | 2 | 0.01 |
| *Porhydrus lineatus* (Fabr.) | L | 42 | 3 | 1 | 64 | 8 | 118 | 0.59 |
| *Hygrotus confluens* (Fabr.) | A | – | 10 | – | – | – | 10 | 0.05 |
| *Hygrotus decoratus* (Gyll.) | T | 20 | 27 | 5 | 26 | 3 | 81 | 0.41 |
| *Hygrotus impressopunctatus* (Schall.) | E | 40 | 72 | 3 | 61 | 8 | 184 | 0.92 |
| *Hygrotus inaequalis* (Fabr.) | E | 105 | 85 | 28 | 266 | 17 | 501 | 2.51 |
| *Hygrotus versicolor* (Schall.) | L | 55 | 1 | 58 | 647 | – | 761 | 3.82 |
| *Scarodytes halensis* (Fabr.) | A | 84 | 1924 | – | 6 | – | 2014 | 10.11 |
| *Hyphydrus ovatus* (L.) | E | 65 | 40 | 27 | 327 | 11 | 470 | 2.36 |
| *Bidessus hamulatus* (Gyll.) | L | 86 | 1 | 40 | 1 | – | 128 | 0.64 |
| *Bidessus unistriatus* Goeze | E | – | – | – | – | 3 | 3 | 0.02 |
| *Hydroglyphus geminus* (Fabr.) | A | 12 | 190 | – | 7 | 4 | 213 | 1.07 |
| *Laccophilus hyalinus* (De Geer) | L | 29 | 2 | 8 | 55 | – | 94 | 0.47 |
| *Laccophilus minutus* (L.) | E | 182 | 101 | – | 39 | 27 | 349 | 1.75 |
| *Laccophilus poecilus* (Klug.) | T | – | – | – | – | 51 | 51 | 0.26 |
| *Laccornis oblongus* (Steph.) | T | – | – | – | 3 | – | 3 | 0.02 |
| *Helophorus aequalis* Thoms. | A | 1 | 13 | 3 | 2 | – | 19 | 0.10 |
| *Helophorus aquaticus* (L.) | A | – | – | 1 | 140 | – | 141 | 0.71 |
| *Helophorus flavipes* (Fabr.) | A | – | – | – | 10 | – | 10 | 0.05 |
| *Helophorus grandis* (Ill.) | A | – | 9 | – | – | – | 9 | 0.05 |
| *Helophorus granularis* (L.) | A | 1 | 3 | – | 7 | 1 | 12 | 0.06 |
| *Helophorus griseus* Herbst | A | 8 | 61 | 1 | – | 1 | 71 | 0.36 |
| *Helophorus minutus* (Fabr.) | A | 37 | 83 | 10 | 1 | 11 | 142 | 0.71 |
| *Helophorus nubilus* (Fabr.) | A | – | 3 | – | – | – | 3 | 0.02 |
| *Helophorus pumilio* (Fabr.) | A | – | – | – | – | 1 | 1 | 0.01 |
| *Hydrochus angustatus* Germ. | E | – | 3 | – | – | – | 3 | 0.02 |
| *Hydrochus brevis* Herbst | E | – | 2 | 2 | 6 | 1 | 11 | 0.06 |
| *Hydrochus crenatus* (Fabr.) | E | 18 | 10 | 1 | 2 | 4 | 35 | 0.18 |
| *Hydrochus elongatus* (Schall.) | E | 6 | 5 | 1 | 1 | 1 | 14 | 0.07 |
| *Hydrochus ignicollis* Motsch. | E | 31 | 7 | 1 | – | – | 39 | 0.20 |
| *Hydrochus nitidicollis* Muls. | E | – | – | 1 | – | 1 | 2 | 0.01 |
| *Anacaena limbata* (Fabr.) | E | – | – | 15 | 12 | – | 27 | 0.14 |
| *Anacaena lutescens* Steph. | T | 152 | 48 | 23 | 39 | 379 | 641 | 3.22 |
| *Cymbiodyta marginella* (Fabr.) | T | 1 | 2 | 3 | 9 | – | 15 | 0.08 |
| *Enochrus affinis* (Thunb.) | T | 2 | 9 | 2 | 3 | 84 | 100 | 0.50 |
| *Enochrus bicolor* (Fabr.) | A | – | – | – | 10 | – | 10 | 0.05 |
| *Enochrus coarctatus* (Gredl.) | T | 3 | 1 | 19 | 8 | 98 | 129 | 0.65 |
| *Enochrus fuscipennis* (Thoms.) | E | 1 | – | – | – | – | 1 | 0.01 |
| *Enochrus isotae* Sharp | E | 1 | – | – | – | – | 1 | 0.01 |
| *Enochrus melanocephalus* (Oliv.) | T | 2 | 1 | 25 | 5 | – | 33 | 0.17 |
| *Enochrus ochropterus* (Marsh.) | E | – | – | 4 | 3 | 58 | 65 | 0.33 |
| *Enochrus quadripunctatus* (Herbst) | E | 13 | 13 | 1 | 14 | 8 | 49 | 0.25 |
| *Enochrus testaceus* (Fabr.) | E | 4 | – | 53 | 57 | 1 | 115 | 0.58 |
| *Helochares griseus* (Fabr.) | E | 60 | 22 | 41 | 112 | 77 | 312 | 1.57 |
| *Helochares lividus* (Forst.) | E | – | – | – | 3 | – | 3 | 0.02 |
| *Helochares punctatus* (Forst.) | E | 4 | 1 | 7 | 32 | 1 | 45 | 0.23 |
| *Hydrobius fuscipes* (L.) | E | 20 | 11 | 10 | 34 | 6 | 81 | 0.41 |
| *Hydrochara caraboides* (L.) | E | 17 | 12 | 4 | 13 | 1 | 47 | 0.24 |
| *Hydrophilus aterrimus* Eschs. | E | 1 | – | 1 | – | – | 2 | 0.01 |
| *Laccobius atrocephalus* Reitter | A | 1 | 4 |  | – | – | 5 | 0.03 |
| *Laccobius biguttatus* (Gerh.) | A | 4 | 3 | 41 | 1 | – | 49 | 0.25 |
| *Laccobius bipunctatus* (Fabr.) | A | 6 | 3 | 3 | 39 | – | 51 | 0.26 |
| *Laccobius minutus* (L.) | A | 165 | 1711 | 57 | 338 | 14 | 2285 | 11.47 |
| *Laccobius sinuatus* Motsch. | A | – | 7 | – | 3 | – | 10 | 0.05 |
| *Laccobius striatulus* (Fabr.) | L | – | 16 | – | – | – | 16 | 0.08 |
| *Spercheus emarginatus* (Schall.) | E | – | – | – | 6 | – | 6 | 0.03 |
| *Coelostoma orbiculare* (Fabr.) | E | 3 | 2 | 11 | 8 | 42 | 66 | 0.33 |
| *Sphaeridium scarabaeoides* (L.) | E | – | – | – | 1 | – | 1 | 0.01 |
| *Chaetarthria seminulum* (Herbst) | E | 9 | 4 | – | – | – | 13 | 0.07 |
| *Cercyon convexiusculus* Steph. | E | – | – | 1 | 1 | – | 2 | 0.01 |
| *Cercyon haemorhoidalis* (Fabr.) | E | – | – | – | 1 | – | 1 | 0.01 |
| *Cercyon marinus* Thoms. | E | 1 | – | – | – | – | 1 | 0.01 |
| *Cercyon tristis* (Ill.) | E | 1 | – | 1 | 2 | – | 4 | 0.02 |
| *Cercyon ustulatus* (Preyssl.) | E | – | – | – | 4 | – | 4 | 0.02 |
| *Hydraena palustris* Er. | T | 8 | 1 | 2 | 1 | – | 12 | 0.06 |
| *Hydraena riparia* (Kugel.) | Rb | 16 | 1 | – | – | – | 17 | 0.09 |
| *Limnebius aluta* (Bedel) | E | 8 | 11 | 12 | 1 | – | 32 | 0.16 |
| *Limnebius atomus* (Duft.) | E | 7 | – | 2 | 2 | – | 11 | 0.06 |
| *Limnebius crinifer* Rey | E | 8 | 5 | 2 | 2 | – | 17 | 0.09 |
| *Limnebius nitidus* (Marsh.) | E | – | – | 1 | – | – | 1 | 0.01 |
| *Limnebius papposus* Muls. | E | 1 | 12 | 4 | – | – | 17 | 0.09 |
| *Limnebius parvulus* Herbst | E | 62 | 42 | 7 | 13 | 5 | 129 | 0.65 |
| *Limnebius stagnalis* (Guill.) | E | – | – | 2 | – | – | 2 | 0.01 |
| *Ochthebius minervius* Orch. | A | – | 2 | – | – | – | 2 | 0.01 |
| *Ochthebius minimus* (Fabr.) | E | 3 | 13 | 2 | 1 | 1 | 20 | 0.10 |
| *Megasternum obscurum* Marsch. | E | – | – | – | 1 | – | 1 | 0.01 |
| *Oulimnius tuberculatus* (O. F. Müll.) | L | – | – | 1 | 1 | – | 2 | 0.01 |
