# Supplementary material for: Water beetle networks differences and migration between natural lakes and post-exploitation water bodies
Source: Sci Rep. 2025 May 7;15:15898. doi: 10.1038/s41598-025-00525-1 (PMC12059192; doi:10.1038/s41598-025-00525-1)
Supplement: Supplementary file 3 — Supplementary Material 3 [file 41598_2025_525_MOESM3_ESM.docx]

Table S2. General characteristics of the lakes: % – contribution in the littoral zone (Sm – *Sphagnum* mat, Di – Diffuse macrophytes, De - Dense macrophytes, A – arenal zone), S – number of species.

| **Water bodies** | **Geographic N** | **Coordinates E** | **Area (ha)** | **Depth**  **mean**  **(m)** | **Cover mat**  **(%)** | **%** | | | |
| --- | --- | --- | --- | --- | --- | --- | --- | --- | --- |
|  |  |  |  |  |  | **Sm** | **Di** | **De** | **A** |
| ***Mesotrophic lakes*** | ­­ | | | | | | | | |
| Babięty | 53° 43′ 07″ | 21° 07′ 15″ | 50.7 | 1.4 | –­­ | –­­ | 0.1 | 0.8 | 0.1 |
| Majcz Wielki | 53° 46′ 44″ | 21° 27′ 29″ | 163.5 | 6.0 | –­­ | –­­ | 0.2 | 0.6 | 0.2 |
| Redykajny | 53° 45′ 55″ | 20° 25′ 04″ | 29.9 | 8.0 | –­­ | –­­ | 0.1 | 0.8 | 0.1 |
| Tyrsko | 53° 48′ 18″ | 20° 25′ 21″ | 18.6 | 9.6 | –­­ | –­­ | 0.1 | 0.7 | 0.2 |
| Wukśniki | 54° 58′ 13″ | 20° 05′ 57″ | 117.1 | 23.3 | –­­ | –­­ | 0.1 | 0.75 | 0.15 |
| ***Eutrophic lakes*** | | | | | | | | | |
| Długie | 53° 47′ 20″ | 20° 27′ 81″ | 26.8 | 5.3 | –­­ | –­­ | 0.2 | 0.7 | 0.1 |
| Dobrąg | 53° 50′ 23″ | 20° 46′ 54″ | 108.0 | 11.5 | –­­ | –­­ | 0.1 | 0.85 | 0.05 |
| Kortowskie | 53° 45′ 43″ | 20° 26′ 44″ | 89.7 | 5.9 | –­­ | –­­ | 0.2 | 0.75 | 0.05 |
| Luterskie | 53° 59′ 36″ | 20° 55′ 57″ | 691.8 | 7.2 | –­­ | –­­ | 0.05 | 0.9 | 0.05 |
| Luterskie 1 | 53° 59′ 36″ | 20° 55′ 57″ | 691.8 | 7.2 | –­­ | –­­ | 0.05 | 0.9 | 0.05 |
| Skanda | 53° 45′ 24″ | 20° 31′ 55″ | 51.1 | 5.8 | –­­ | –­­ | 0.15 | 0.8 | 0.05 |
| ***Dystrophic lakes*** | | | | | | | | | |
| Bobrówko | 53°45’02’’ | 21°31’11’’ | 1.30 | 1.1 | 40 | 0.9 | 0.1 | –­­ | – |
| Borkowskie | 53°43’29’’ | 21°32’89’’ | 2.70 | 1.6 | 37 | 0.9 | 0.1 | – | – |
| Białe | 53°53’29’’ | 20°49’21’’ | 2.88 | 1.7 | 3 | 0.8 | 0.1 | 0.1 | – |
| Gryżewskie | 53°43’39’’ | 21°32’48’’ | 4.0 | 3.6 | 42 | 0.5 | 0.1 | 0.4 | 0.1 |
| Krucze Oko | 53°39’33’’ | 21°24’11’’ | 0.4 | 1.1 | 35 | 0.9 | 0.1 | – | – |
| Kruczy Staw | 53°39’30’’ | 21°24’14’’ | 2.08 | 1.1 | 15 | 0.9 | 0.1 | – | – |
| Jonkowo | 53°48’49’’ | 20°19’05’’ | 2.95 | 1.3 | 20 | 0.8 | 0.1 | 0.1 | – |
| Klimontek | 53°42’21’’ | 21°26’07’’ | 0.40 | 1.2 | 60 | 0.9 | 0.1 | – | – |
| Kociołek | 53°54’01’’ | 20°49’11’’ | 0.56 | 1.6 | 5 | 0.9 | 0.1 | – | – |
| Kośno (Purdka) | 53°48’49’’ | 20°19’05’’ | 1.61 | 1.3 | 43 | 0.8 | 0.1 | 0.1 | – |
| Kruczek Duży | 53°39’33’’ | 21°24’11’’ | 4.24 | 1.3 | 20 | 0.9 | 0.1 | – | – |
| Kruczek Mały | 53°39’27’’ | 21°25’01’’ | 2.56 | 0.6 | 10 | 0.9 | 0.1 | – | – |
| Motylek | 53°40’16’’ | 20°05’46’’ | 3.30 | 1.6 | 39 | 0.8 | 0.2 | – | – |
| Skarp | 53°38’09’’ | 21°27’42’’ | 13.0 | 6.0 | 1 | 0.1 | 0.3 | 0.6 | – |
| Żabie | 53°50’00’’ | 15°02’00’’ | 1.78 | 1.2 | 70 | 0.8 | 0.1 | 0.1 | – |
| ***Clay pits*** | | | | | | | | |  |
| Najdymowo (CP_1, CP_2, CP_3) | 53⁰52’18” – 53⁰52’27” | 20⁰53’33” –20⁰53’35” | 0.09 – 1 | 3 | compact cornfields of  *Phragmites australis* | | | | |
| Parleza Wielka (CP_4 – CP_11) | 53⁰51’03” – 53⁰51’12” | 21⁰00’26” – 21⁰00’37” | 0.001 – 1 | 1.2 | complete absence – sparse and diverse – compact cornfields of  *Phragmites australis* | | | | |
| ***Gravel pits*** | | | | | | | | | |
| Giławy (GP_12, GP_13) | 53⁰43’37” | 20⁰48’03” | 0.5 | 1.5 | complete absence – sparse and diverse – compact cornfields of | | | | |
| Parleza Mała (GP_14 – GP_22) | 53⁰50’24” | 21⁰01’02” | 0,0001 – 1 | 0.4 |  |  |  |  |  |
| Mątki (GP_23 – GP_27 | 53⁰49’31” | 20⁰20’28” | 0,0001 – 0.01 | 0.8 | complete absence – sparse and diverse – compact cornfields of | | | | |
| Kronowo (GP_28 – GP_31) | 53⁰52’42” | 20⁰42’29” | 0,0001– 0.0006 | 0.4 |  |  |  |  |  |
